# Alkyltransferase Ribozyme for Site‐Specific *N* ^4^‐Cytidine Alkylation

**DOI:** 10.1002/anie.6447137

**Published:** 2026-04-06

**Authors:** Evgeniia Dorinova, Manisha B. Walunj, Claudia Höbartner

**Affiliations:** ^1^ Institute of Organic Chemistry Julius‐Maximilians‐Universität Würzburg Am Hubland Würzburg Germany; ^2^ Center for Nanosystems Chemistry Julius‐Maximilians‐Universität Würzburg Theodor‐Boveri‐Weg Würzburg Germany; ^3^ Cluster for Nucleic Acid Sciences and Technologies—NUCLEATE Würzburg Germany

**Keywords:** cytidine modification, fluorescent labeling, in vitro selection, ribozyme, RNA labeling

## Abstract

Ribozymes for site‐specific RNA modification provide an elegant approach for the installation of diverse functional groups, fluorophores, affinity tags, or crosslinkers at defined positions within an RNA of interest. There is increasing interest in expanding the ribozyme toolbox, since recently reported in vitro selected ribozymes have been mostly limited to labeling at adenosine sites, either by alkylation of the nucleobase or phosphodiester formation at the 2’‐OH group. Here we report a cytidine‐specific alkyltransferase ribozyme (CSAR) that uses *O* ^6^‐benzylguanines as alkyl group donors. CSAR is the first ribozyme that catalyzes direct alkylation of the exocyclic amino group of a nucleobase and generates *N* ^4^‐alkylated cytidine in a defined sequence context of a short RNA hairpin loop. In combination with tuning the electronic parameters of the transferred benzyl group, CSAR enables highly efficient cytidine alkylation for the installation of bioorthogonal functional groups.

## Graphical abstract

CSAR is the first ribozyme that catalyzes direct alkylation of an exocyclic amino group of an RNA nucleobase. The ribozyme was isolated as a self‐labeling catalyst from an in vitro selection library and engineered into a *trans*‐active ribozyme that catalyzes the transfer of a benzyl group from an *O^6^*‐modifed guanine to the *N*4 amino group of a specific cytidine in the loop of an RNA hairpin.

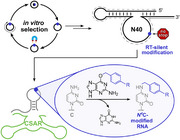

## 1 Introduction

Ribozymes found in nature catalyze a limited range of reactions, mostly involving the transformation of phosphodiester bonds, while ribozymes generated in the laboratory by in vitro selection engage in more diverse chemistry [1, 2, 3]. Since the early 1990's, numerous synthetic ribozymes have been described, including some self‐alkylating ribozymes [4, 5, 6, 7]. The research on RNA‐catalyzed RNA modification and labeling [8, 9] has received increasing attention in the past five years, since the discovery of the first methyltransferase ribozyme (MTR1) [10]. MTR1 uses *O* ^6^‐methylguanine (m^6^G) as methyl group donor and enables the methylation of a specific adenosine at position *N*1 [10], thus mimicking natural methyltransferase protein enzymes that install 1‐methyladenosine (m^1^A) in tRNA [11]. Additional ribozymes that catalyze RNA methylation include the SAM‐dependent methyltransferase ribozyme (SMRZ‐1) that generates 7‐methylguanosine [12], and the repurposed preQ1 class I riboswitch, which produces 3‐methylcytidine (m^3^C) in the presence of the synthetic cofactor *O* ^6^‐methyl prequeuosine (m^6^preQ1) [13]. Another recent addition is the SAM‐analogue‐utilizing ribozyme (SAMURI), which transfers a propargyl group to position *N*3 of adenosine [14]. Installation of the small clickable tag by SAMURI enables further functionalization for in vitro and intracellular applications. Moreover, SAMURI also catalyzes RNA methylation using natural SAM or a synthetic selenium‐based SAM analogue (MeSeDMA), resulting in 3‐methyladenosine (m^3^A) at a defined position within an RNA of interest [15].

Along the lines of site‐specific RNA labeling by alkyltransferase ribozymes, MTR1 was shown to use various *O* ^6^‐benzylguanines that are known as established SNAP‐tag [16] substrates for protein labeling, and that can now be repurposed for site‐directed modification of RNA [17]. For this purpose, MTR1 is also referred to as SNAPR, and the family of ribozymes targeting adenosines keeps expanding. A recent addition is the RNA‐alkylating catalytic RNA (RACR), which employs a different mode of target binding [18]. Besides direct alkylation of *N*1 of adenosine, SNAPR and RACR were employed in a two‐step approach to yield *N* ^6^A‐modified RNA through Dimroth rearrangement [17, 18]. Furthermore, MTR1/ SNAPR was repurposed for the synthesis of aldehyde‐functionalized RNAs and subsequent bioconjugation reactions when targeting a nebularine‐containing RNA [19].

**FIGURE 1:**
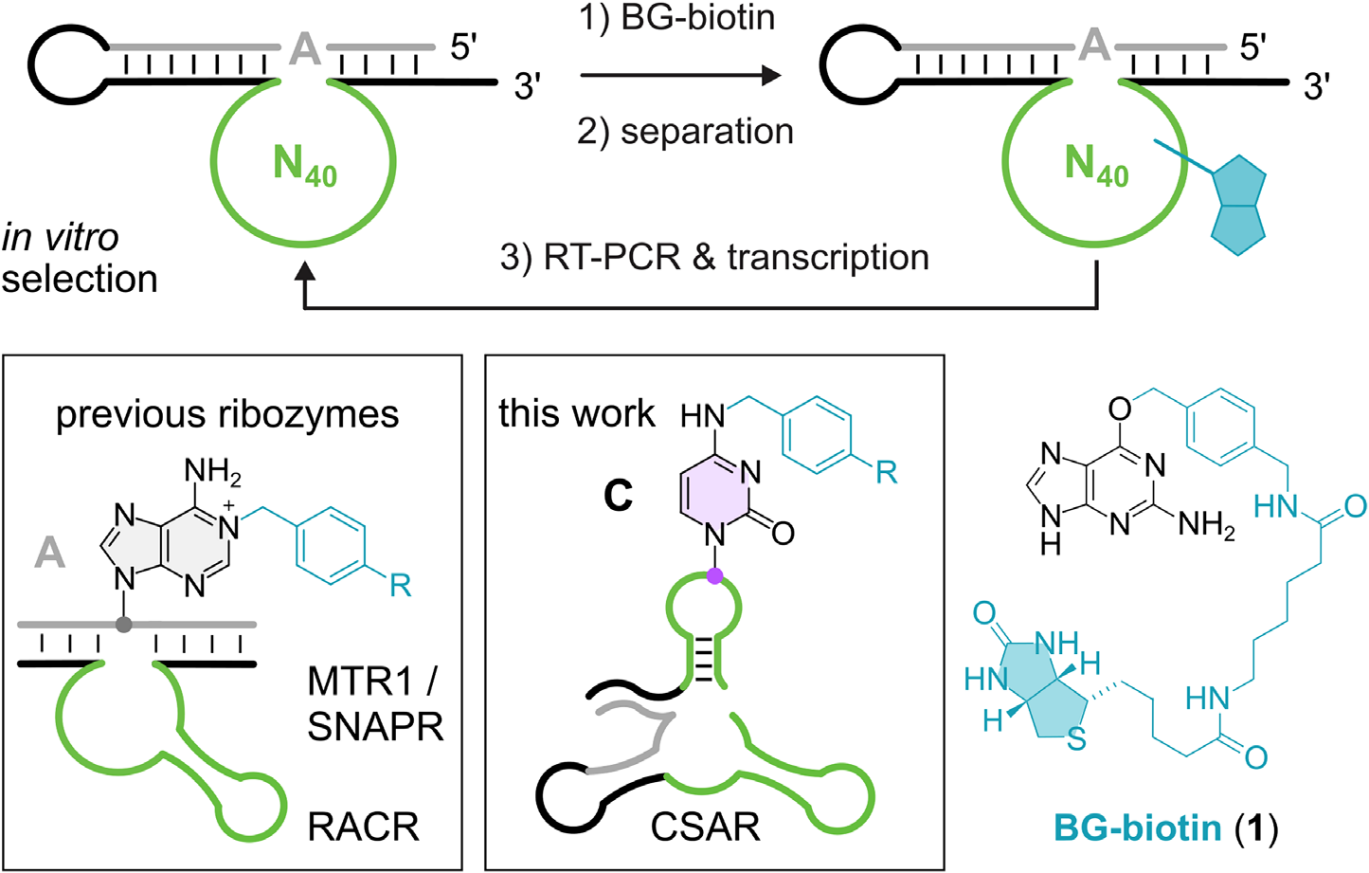
In vitro selection scheme for identification of alkyltransferase ribozymes using BG‐biotin, and schematic depiction of activity of previously reported adenosine‐alkylating ribozymes and the cytidine‐specific ribozyme identified in this work.

Despite these advances, the diversity of RNA modifications accessible by catalytic RNAs is still limited. All previously reported RNA‐alkylating ribozymes target endocyclic nitrogen atoms of nucleobases and generate a positive charge in the alkylated product [20]. In contrast, direct alkylation of exocyclic amino groups by RNA‐modifying ribozymes has not been reported so far and seems to be more challenging due to the lower nucleophilicity of exocyclic amino groups compared to ring nitrogen atoms [21]. Enzymes can easily overcome such reactivity difference, as exemplified by the diversity of methyltransferases that generate biologically highly relevant methylated nucleotides in RNA, such as m^6^A, m^4^C, or m^2^G [22, 23]. Notably, some nucleic acid enzymes have been reported to modify exocyclic amino groups, including the DNAzyme‐catalyzed reductive amination of the exocyclic amino group of cytidine [24], *N*‐acylation of DNA nucleobases by deoxyribozymes [25], as well as RNA‐catalyzed phosphorylation at *N*2 of guanosine [26]. Given these previous examples, it seems feasible that ribozymes can be found that target exocyclic amino groups by direct alkylation, for example, by nucleophilic displacement of a leaving group. Here, we report the first ribozyme that catalyzes the direct *N*‐benzylation of cytidine at the exocyclic *N*4 amino group (Figure 1). The new cytidine‐specific alkyltransferase ribozyme (CSAR) uses derivatives of *O* ^6^‐benzyl‐guanine as alkyl donor with guanine as the leaving group to generate *N* ^4^‐modified cytidine in a defined sequence context of a short RNA that hybridizes to the ribozyme *in trans* via a variable binding arm.

## 2 Results and Discussion

CSAR was found from an in vitro selection experiment that was performed in analogy to the experiments that resulted in the discovery of MTR1 and RACR [10, 18]. The initial RNA library contained 40 random nucleotides flanked by two Watson–Crick base‐paired binding arms, and an unpaired nucleotide as a potential modification site in the putative substrate RNA (gray region in Figure 1). The RNA library was incubated with biotinylated *O* ^6^‐benzylguanine (BG‐biotin, **1**) followed by separation of modified RNA from unreacted sequences by pull‐down on streptavidin or neutravidin‐coated magnetic beads. The eluted RNA was reverse transcribed, amplified by two‐step PCR, and the obtained double‐stranded DNA was in vitro transcribed by T7 RNA polymerase to generate the RNA library for the next selection round (Figure S1). After eight rounds, individual sequences were identified and tested for their activity. We found a new ribozyme family that was unrelated to any of our previously characterized ribozymes. The streptavidin gel shift assay showed good activity (Figure S1), but primer extension experiments did not reveal any obvious stop signal, neither in the putative substrate region, nor in the rest of the ribozyme sequence, including the catalytic core (Figure S2).

**FIGURE 2:**
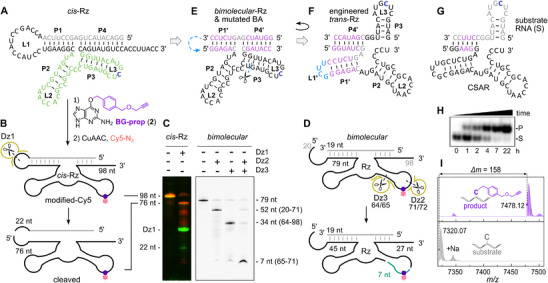
(A) Predicted secondary structure of the *cis*‐active ribozyme (*cis*‐Rz). (B) Scheme of *cis*‐Rz reaction with BG‐prop (**2**) followed by CuAAC with Cy5‐N_3_ and DNA‐catalyzed cleavage by Dz1. (C) Denaturing PAGE image. Left: overlay of Cy5 channel (red) and SYBR gold stain (green), before and after cleavage with Dz1; right: Cy5 channel image of (D) cleavage products of reacted and Cy5‐labeled bimolecular Rz construct cleaved with Dz2 and Dz3, to narrow down the labeling position to the 7‐nt stretch shown in turquoise (D). (E) Predicted secondary structure of the bimolecular ribozyme generated by removing the connecting loop (15 nt, L1) and using mutated binding arms (BA, P1', P4' pink). (F) An engineered *trans*‐active ribozyme (Rz5) modifying the blue cytidine in an external RNA substrate generated by linking the original RNA substrate and the ribozyme core with 5' UUCG 3' tetraloop (cyan) and cutting the phosphodiester bond of the respective U64 and G65 in P3 as shown by the scissors icon in (E). (G) Engineered CSAR with an alternative sequence in the binding arm (Rz6). (H, I) Representative image of PAGE analysis for the substrate RNA (Tr3) (H) and deconvoluted HR‐ESI mass spectra for the substrate RNA (R1) (I) of CSAR‐catalyzed alkyl transfer reactions using BG‐prop (**2**) as cofactor. Uncropped gel images are provided in the Supporting Information.

These results suggest that biotin was installed at a position that does not block the Watson–Crick face of any nucleobase outside of the primer binding sites. When the connecting loop was removed, we could not detect any modification of the putative shorter substrate RNA in a bimolecular (*in trans*) setup (Figure S3). However, when the intact ribozyme (*cis*‐Rz, Figure 2A) was incubated with *O* ^6^‐(4‐(propargyloxymethyl)benzyl)guanine (BG‐prop, **2**), and the product was subjected to CuAAC with Cy5‐azide, we found a Cy5‐labeled 98‐nt‐long RNA (Figure 2B). The emergence of this band shows that the ribozyme‐catalyzed reaction had occurred with **2**, indicating that biotin is dispensable and that the transferred alkyne was intact and could be conjugated to the fluorophore. Upon cleaving the resulting Cy5‐labeled 98‐nt long RNA with an 8–17 deoxyribozyme at position 22 (Dz1), that is, separating the putative substrate sequence from the ribozyme core after the RNA‐catalyzed reaction of *cis*‐Rz, a Cy5‐labeled 76‐mer and an unlabeled 22‐mer were produced (Figure 2C).

**FIGURE 3:**
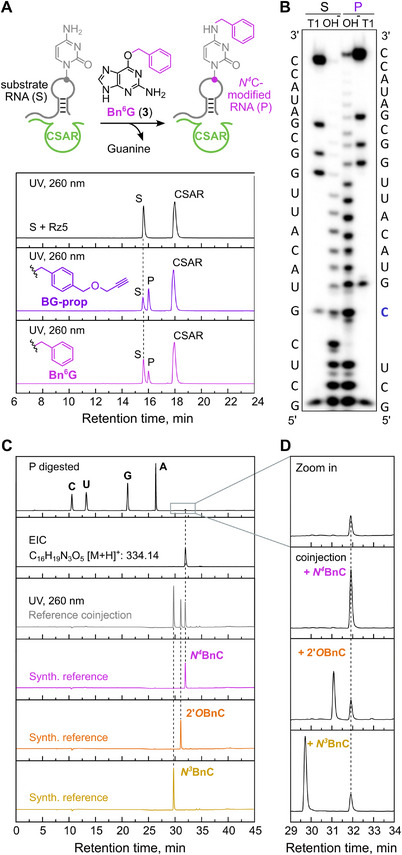
Determination of the modification site of CSAR. (A) Reaction scheme and analysis of reactions with **2** or **3** by anion exchange HPLC monitored at 260 nm. (B). Representative gel image for RNase T1 digestion and alkaline hydrolysis of 5'‐^32^P‐labeled reaction product (P) generated by CSAR using **2** in comparison to unmodified substrate RNA (S). The modification site is marked in blue. Uncropped gel image is available in the Supporting Information. (C) LC‐MS analysis of the digested product (P) resulting from reaction with **3**. UV trace at 260 nm and extracted ion chromatogram (EIC (*m/z* (M+H^+^) = 334.14 ± 0.02)). Synthetic references *N4*BnC, *2'O*‐BnC, and *N^3^* BnC (UV traces at 260 nm in magenta, orange, and yellow, respectively) along with the coinjection of the three synthetic standards (gray). (D) Zoom in (of UV traces at 260 nm) and individual coinjections of the digested modified product from **C** with each of the synthetic references.

This result indicates that the propargylated benzyl group was installed within the ribozyme core sequence. Secondary structure prediction [27] shows the architecture of the selection library with two base‐paired binding arms (P1 and P4) and the connecting loop L1, and suggests that the ribozyme core folds into two stem‐loops (P2, L2 and P3, L3) as shown in Figure 2A. Based on this prediction, we tested if the catalytic activity was maintained in a bimolecular *trans* setup (i.e. when L1 was cut). Instead of monitoring only the short substrate fragment, we evaluated the hybridized complex (19‐nt substrate strand + 79‐nt ribozyme strand) after reaction with **1** by a streptavidin gel‐shift assay (Figure S3), and by denaturing PAGE and fluorescence imaging after reaction with **2** followed by Cy5‐labeling (Figure 2C). Indeed, we found a modified (labeled) 79‐mer in both experiments. Using two additional 8–17 DNAzymes (Dz2 and Dz3) designed to cut at position 71 and 64, respectively, we narrowed down the modification site to a stretch of 7 nucleotides in the core region of the ribozyme, that is between position 65 and 71 (Figure 2D). The presence of the 19‐nt strand forming P1 and P4 was necessary for the activity, but the sequence of the binding arm duplexes P1 and P4 could be exchanged (pink nucleotides in Figure 2E), and activity was retained when the loop L1 and the 8‐nt 3’‐overhang were removed (Figure S3). Based on these results, and using the predicted secondary structure of *cis*‐Rz and the Dz3 cutting site a U64/G65, we re‐defined “substrate” and “ribozyme” regions and engineered a *trans* variant, in which the new stem P1' was closed with a stable UUCG tetraloop, and P4' constitutes the binding arm that hybridizes to the new substrate RNA containing the previous P3‐L3 stem‐loop (Figure 2F). By changing the sequence of P4', a second substrate/ribozyme combination was designed (Figure 2G and S4). Activity *in trans* of both engineered constructs (Figure 2F,G) was confirmed by annealing the ribozyme strand with the RNA substrate and incubation with cofactor **2**. Product formation was detected via polyacrylamide gel electrophoresis (PAGE) resulting in a mobility shift due to the higher molecular mass of the alkylated product compared to the unmodified substrate RNA (Figure 2H). Product formation was also detected by anion‐exchange HPLC (Figure 3A), and mass spectrometry of the isolated product confirmed that the 4‐(propargyloxymethyl)benzyl group has been transferred to the RNA (Figure 2I).

To determine the exact position of the alkylated nucleotide within the 7‐nt stretch identified by DNAzyme cleavage assays, the alkylated 23‐mer RNA product was subjected to alkaline hydrolysis and RNase T1 digestion (Figure 3B). The shifted band pattern generated from the modified RNA in comparison to an unmodified reference clearly identified cytidine C7 as the modified nucleotide. When this nucleotide C7 was mutated to any of the other three nucleotides, no product formation was detected by anion‐exchange HPLC or PAGE (Figure S3), further supporting the conclusion that the reaction happened at C7.

However, the exact reaction site, that is, which nitrogen or oxygen atom of C7 was alkylated, remained to be identified. We confirmed that *O* ^6^‐benzylguanine (Bn^6^G, **3**) was also accepted as alkyl donor (Figure 3A) and isolated the benzylated RNA product, which was then digested into mononucleosides by snake venom phosphodiesterase (SVPD) and bacterial alkaline phosphatase (BAP). The resulting nucleosides were analyzed by liquid chromatography–mass spectrometry (LC–MS) in comparison with synthetic standards of 2'‐*O*‐benzylcytidine (2'*O*BnC), *N* ^4^‐benzylcytidine (*N* ^4^BnC), and 3‐benzylcytidine (*N* ^3^BnC) (Figure 3C,D). A modified nucleoside was found in the UV trace at 260 nm and in the extracted ion chromatogram (EIC) of 334.14 *m/z*, corresponding to a benzylated cytidine. The retention time of the new benzylated product was in accordance with that of *N* ^4^BnC. Coinjection of the three references (Figure 3C), as well as individual coinjections of the digested RNA with each of the references (Figure 3D) allowed to clearly identify the product as *N* ^4^BnC. As can be seen in the zoomed‐in region of the HPLC chromatogram, supplementing the digestion mixture with 2'*O*BnC or *N* ^3^BnC, resulted in separate peaks, while the *N* ^4^BnC reference co‐eluted with the new product, resulting in a single peak of increased intensity (Figure 3D). Moreover, the same peak was found in the EIC of 202.10 *m/z*, corresponding to benzylated cytosine, which may have formed due to fragmentation of *N* ^4^‐benzylcytidine in the mass spectrometer (Figure S5), while a potential ribose‐modified fragment with EIC of 223.10 *m/z* corresponding to a plausible fragmentation of 2'*O*BnC was not detected. All together, these combined data support the conclusion that the new ribozyme installs the modification as *N* ^4^‐alkylated cytidine at C7 of the substrate RNA. For these reasons, the new ribozyme was named CSAR (Cytosine‐Specific Alkyltransferase Ribozyme).

**FIGURE 4:**
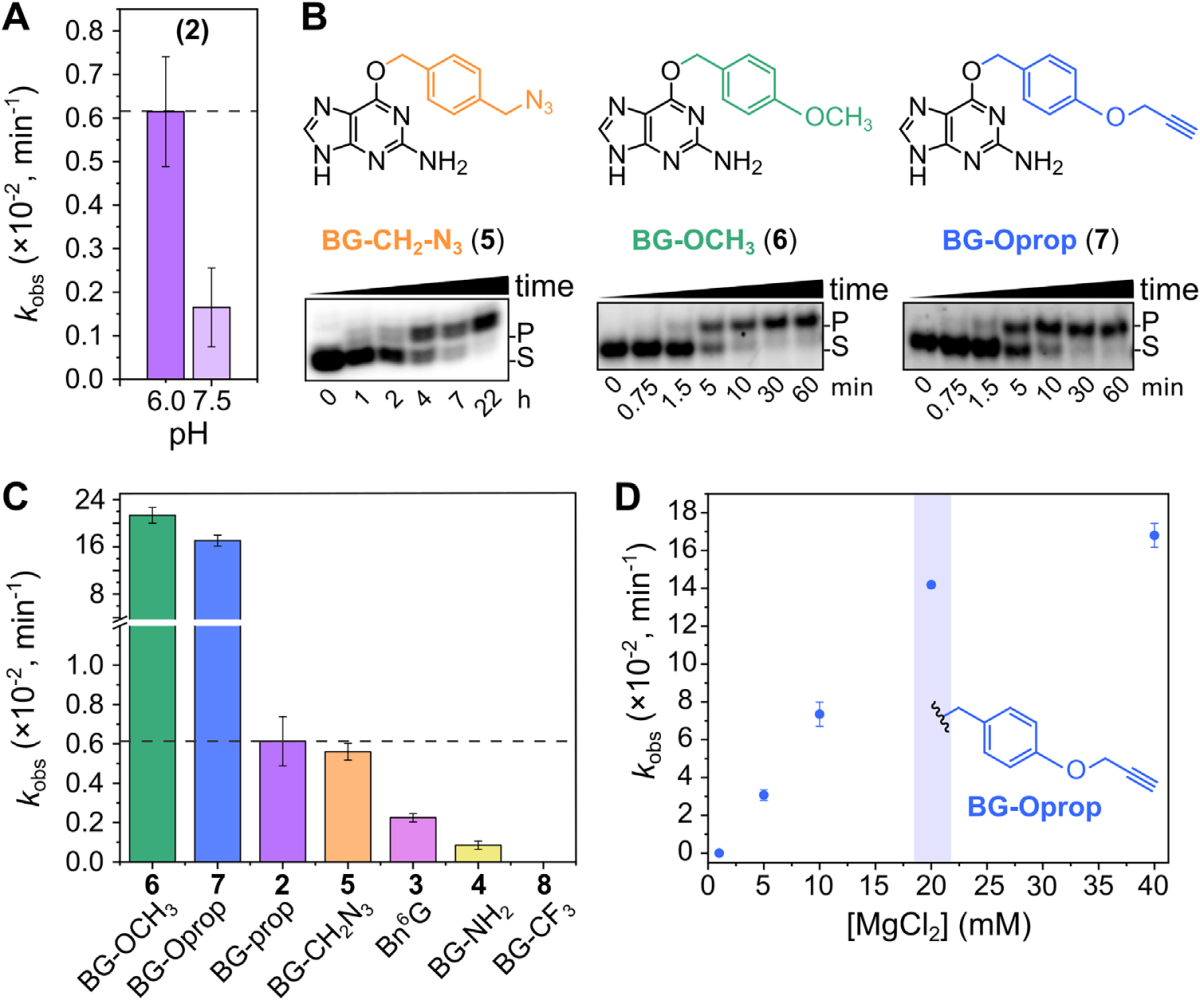
Kinetic characterization and cofactor scope of CSAR. (A) *k*_obs_ values obtained from reactions at pH 6.0 and pH 7.5 using BG‐prop (**2**) under single turnover conditions (100 µM **2**, 37°C). (B) Exemplary PAGE images and (C) *k_obs_* values from kinetic assays using different *O* ^6^‐modified guanines (1 µM substrate RNA, 10 µM CSAR, 100 µM cofactor, pH 6.0, 40 mM MgCl_2_, 37°C, 1 h (for fast reactions) or 22 h (for slow reactions)). The dotted line serves as guide for comparison to cofactor **2** as the reference. (D) MgCl_2_ dependency of CSAR with BG‐Oprop (**7**) (100 µM **7**, pH 6.0, 37°C). All experiments were performed under single turnover conditions with the 22‐mer RNA substrate and CSAR shown in Figure 2G. Data represent mean values of three independent experiments. Uncropped gel images are available in the Supporting Information.

The catalytic activity of CSAR was then characterized in more detail. Alkylation kinetics were determined under single‐turnover conditions using a 22‐mer 3'‐fluorescently or 5'‐^32^P‐labeled substrate RNA and BG‐prop (**2**) as cofactor. The reactions were run in different buffer conditions, and aliquots at various time points were resolved on PAGE, followed by quantification of the fraction modified. The alkylation reaction catalyzed by CSAR proceeds 3.8‐fold faster at pH 6.0 compared to the selection conditions at pH 7.5 (Figure 4A, Figure S6), resulting in 90% conversion after overnight incubation. Good conversion was also achieved with other *O* ^6^‐benzylguanine derivatives, including BG‐NH_2_ (**4**) and BG‐CH_2_N_3_ (**5**) (Figure 4B,C and Figure S6).

Interestingly, the reaction proceeded much faster with *O* ^6^‐(4‐(methoxy)benzyl)guanine (BG‐OCH_3_, **6**), resulting in almost quantitative alkylation in less than 30 min. This effect is similar to the recently observed rate acceleration by electron‐donating substituents on Bn^6^G for RACR [18]. To further exploit this beneficial effect, we synthesized *O* ^6^‐(4‐(propargyloxy)benzyl)guanine (BG‐Oprop, **7**) bearing a bioorthogonal alkyne handle for the subsequent attachment of clickable tags. The transfer of a 4‐(propargyloxy)benzyl (Bn‐Oprop) group achieved high yields (87% alkylation product) within 1 h. The *k*_obs_ of the alkylation reactions were enhanced by a factor of 31 and 25 for **6** and **7**, respectively, compared to the transfer of the 4‐(propargyloxymethyl)benzyl‐moiety from **2**. In contrast, when employing a cofactor with an electron‐withdrawing group, such as *O* ^6^‐(4‐(trifluoromethyl)benzyl)guanine (BG‐CF_3_, **8**), no product formation was observed (Figures 4C and S6). This lack of reactivity may also be due to weaker binding of cofactor **8**, as suggested by a competition experiment with cofactor **2** that showed only minor inhibition (Figure S7). The reaction rate of CSAR is generally dependent on cofactor concentration, as observed previously for MTR1 and RACR, and is consistent with the conditions used during in vitro selection (100 µM cofactor).

Moreover, we examined the sensitivity of CSAR to divalent metal ions, and found that the concentration of Mg^2+^ can be reduced 8‐fold from the original 40 mM to 5 mM Mg^2+^ retaining a good yield with cofactor **7** (60% over 2 h), although with ca 5‐fold slower *k*_obs_ = 0.03 min^−1^ (compared to *k*_obs_ = 0.17 min^−1^ at 40 mM Mg^2+^). The best concentration for further use is 20 mM MgCl_2_ resulting in 82% alkylated product within 1 h (*k*_obs_ = 0.14 min^−1^, Figure 4D). Using these optimized conditions, we demonstrated that the propargyloxy‐benzyl group from **7** was also faithfully transferred to the same target cytidine C7 in two substrate RNAs with different binding arms to the ribozyme (Figure S8). The transferred alkyne was similarly accessible for further labeling with FAM or Cy5 under standard CuAAC reaction conditions (Figure S9). The reactions were analyzed by PAGE, and the resulting products were isolated and their identity confirmed by HR‐ESI‐MS (Figure S9, Table S6).

## 3 Conclusion

In summary, this work described the identification and characterization of the first ribozyme for site‐specific cytidine alkylation. CSAR was engineered from a self‐modifying ribozyme that catalyzes the formation of *N* ^4^‐alkylcytidine in the originally random region of the RNA library utilizing various *O* ^6^‐benzylguanine cofactors. The resulting ribozyme can be employed *in trans* by hybridization to the tail of a short RNA hairpin, which may be extended by an RNA of interest. Thus, the application of CSAR for RNA labeling is conceptually comparable to chemoenzymatic approaches that use an anticodon stem loop or tRNA as recognition tags, as previously demonstrated, for example, via enzymatic guanosine transglycosylation, agmatidine synthetase, or methyltransferases, with their respective cofactor analogs [28, 29, 30]. In contrast to these protein‐based methods, CSAR is an RNA‐only technology that can repurpose established SNAP‐tag substrates for RNA labeling, as demonstrated by the attachment of functional moieties to the benzylated guanine.

Beyond its potential for RNA labeling, the discovery of CSAR revealed more fundamental insights about the scope of ribozyme catalysis and has implications for future in vitro selection experiments. The structure‐activity data provide first hints toward the catalytic mechanism of CSAR. The pH and Mg^2+^ dependence and the cofactor scope of CSAR may resemble some of the features previously found for the mechanism of MTR1, which involves a protonated nucleobase in the active site [31, 32, 33]. Future analyses including 3D structure determination will provide a more detailed understanding of CSAR's mechanism and reveal how the proposed hairpin of the substrate RNA interacts with the catalytic core, since current structure prediction tools cannot yet reliably reveal the architecture of the active site [34, 35]. Overall, the discovery of CSAR adds another important milestone for the fundamental understanding of the functions of RNA as a catalyst and suggests that ribozymes for alkylation of exocyclic amino groups of adenosine and guanosine may also be identified in the future.

## Conflicts of Interest

The authors declare no conflicts of interest.

## Supporting information




**Supporting File 1**: Materials and Methods, Supporting Information Tables S1–S6, Supporting Information Figures S1–S10, NMR spectra.

**Supporting File 2**: anie72108‐sup‐0002‐CSAR_Supplement_Uncropped images.pdf.

## Data Availability

The data that support the findings of this study are available in the Supporting Information of this article.

## References

[1] G. F. Joyce , “Directed Evolution of Nucleic Acid Enzymes,” Annual Review of Biochemistry 73 (2004): 791–836, 10.1146/annurev.biochem.73.011303.073717. 15189159

[2] R. Micura and C. Höbartner , “Fundamental Studies of Functional Nucleic Acids: Aptamers, Riboswitches, Ribozymes and DNAzymes,” Chemical Society Reviews 49 (2020): 7331–7353, 10.1039/d0cs00617c. 32944725

[3] J. A. Doudna and T. R. Cech , “The Chemical Repertoire of Natural Ribozymes,” Nature 418 (2002): 222–228, 10.1038/418222a. 12110898

[4] C. Wilson and J. W. Szostak , “In Vitro Evolution of a Self‐Alkylatlng Ribozyme,” Nature 374 (1995): 777–782, 10.1038/374777a0. 7723823

[5] R. I. McDonald , J. P. Guilinger , S. Mukherji , E. A. Curtis , W. I. Lee , and D. R. Liu , “Electrophilic Activity‐Based RNA Probes Reveal a Self‐Alkylating RNA for RNA Labeling,” Nature Chemical Biology 10 (2014): 1049–1054, 10.1038/nchembio.1655. 25306441 PMC4232462

[6] A. K. Sharma , J. J. Plant , A. E. Rangel , et al., “Fluorescent RNA Labeling Using Self‐alkylating Ribozymes,” ACS Chemical Biology 9 (2014): 1680–1684, 10.1021/cb5002119. 24896502

[7] S. Ameta and A. Jäschke , “An RNA Catalyst That Reacts With a Mechanistic Inhibitor of Serine Proteases,” Chemical Science 4 (2013): 957–964, 10.1039/c2sc21588h.

[8] M. G. Maghami , S. Dey , A. K. Lenz , and C. Höbartner , “Repurposing Antiviral Drugs for Orthogonal RNA‐Catalyzed Labeling of RNA,” Angewandte Chemie International Edition 59 (2020): 9335–9339, 10.1002/anie.202001300. 32162405 PMC7318677

[9] M. G. Maghami , C. P. M. Scheitl , and C. Höbartner , “Direct in Vitro Selection of Trans‐Acting Ribozymes for Posttranscriptional, Site‐Specific, and Covalent Fluorescent Labeling of RNA,” Journal of the American Chemical Society 141 (2019): 19546–19549, 10.1021/jacs.9b10531. 31778306

[10] C. P. M. Scheitl , M. Ghaem Maghami , A. K. Lenz , and C. Höbartner , “Site‐Specific RNA Methylation by a Methyltransferase Ribozyme,” Nature 587 (2020): 663–667, 10.1038/s41586-020-2854-z. 33116304 PMC7116789

[11] S. Oerum , C. Degut , and P. Barraud , “m1A Post‐Transcriptional Modification in tRNAs,” Biomolecules 7 (2017): 20, 10.3390/biom7010020. 28230814 PMC5372732

[12] H. Y. Jiang , Y. Q. Gao , L. Zhang , D. R. Chen , J. H. Gan , and A. I. H. Murchie , “The Identification and Characterization of a Selected SAM‐Dependent Methyltransferase Ribozyme That Is Present in Natural Sequences,” Nature Catalysis 4 (2021): 872–881, 10.1038/s41929-021-00685-z.

[13] L. Flemmich , S. Heel , S. Moreno , K. Breuker , and R. Micura , “A Natural Riboswitch Scaffold With Self‐Methylation Activity,” Nature Communications 12 (2021): 3877, 10.1038/s41467-021-24193-7. PMC8222354 34162884

[14] T. Okuda , A. K. Lenz , F. Seitz , J. Vogel , and C. Höbartner , “A SAM Analogue‐Utilizing Ribozyme for Site‐Specific RNA Alkylation in Living Cells,” Nature Chemistry 15 (2023): 1523–1531, 10.1038/s41557-023-01320-z. PMC10624628 37667013

[15] H. A. Chen , T. Okuda , A. K. Lenz , C. P. M. Scheitl , H. Schindelin , and C. Höbartner , “Structure and Catalytic Activity of the SAM‐utilizing Ribozyme SAMURI,” Nature Chemical Biology (2025): 1–10, 10.1038/s41589-024-01808-w. 39779902 PMC13226088

[16] A. Keppler , S. Gendreizig , T. Gronemeyer , H. Pick , H. Vogel , and K. Johnsson , “A General Method for the Covalent Labeling of Fusion Proteins With Small Molecules in Vivo,” Nature Biotechnology 21 (2003): 86–89, 10.1038/nbt765. 12469133

[17] M. B. Walunj , C. P. M. Scheitl , T. Jungnickel , and C. Höbartner , “Ribozyme‐Catalyzed Site‐Specific Labeling of RNA Using O(6)‐alkylguanine SNAP‐Tag Substrates,” Angewandte Chemie International Edition 64 (2025): e202500257, 10.1002/anie.202500257. 40231624 PMC12184302

[18] C. P. M. Scheitl , E. Dorinova , S. Christopher , et al., “An Unexpected Adenosine‐Alkylating Ribozyme Emerged by Target Site Relocation during in Vitro Selection,” Journal of the American Chemical Society 147 (2025): 44225–44235, 10.1021/jacs.5c13970. 41266282

[19] C. P. M. Scheitl , T. Okuda , J. Adelmann , and C. Höbartner , “Ribozyme‐Catalyzed Late‐Stage Functionalization and Fluorogenic Labeling of RNA,” Angewandte Chemie International Edition 62 (2023): e202305463, 10.1002/anie.202305463. 37278361

[20] C. P. M. Scheitl and C. Höbartner , “Ribozymes for RNA‐Catalyzed RNA Methylation and Labeling,” Angewandte Chemie International Edition (2025), 10.1002/anie.202522926. 41998816

[21] D. Gillingham and S. Geigle , “Properties and Reactivity of Nucleic Acids Relevant to Epigenomics, Transcriptomics, and Therapeutics,” Chemical Society Reviews 45 (2016): 2637–2655, 10.1039/c5cs00271k. 26992131

[22] T. R. Fischer , L. Meidner , M. Schwickert , et al., “Chemical Biology and Medicinal Chemistry of RNA Methyltransferases,” Nucleic acids research 50 (2022): 4216–4245, 10.1093/nar/gkac224. 35412633 PMC9071492

[23] C. Höbartner , K. E. Bohnsack , and M. T. Bohnsack , “How Natural Enzymes and Synthetic Ribozymes Generate Methylated Nucleotides in RNA,” Annual Review of Biochemistry 93 (2024): 109–137, 10.1146/annurev-biochem-030222-112310. 38598854

[24] R. D. Boyd , M. M. Kennebeck , A. A. Miranda , Z. Liu , and S. K. Silverman , “Site‐Specific N‐Alkylation of DNA Oligonucleotide Nucleobases by DNAzyme‐Catalyzed Reductive Amination,” Nucleic acids research 52 (2024): 8702–8716, 10.1093/nar/gkae639. 39051544 PMC11347174

[25] M. M. Kennebeck , C. K. Kaminsky , M. A. Massa , et al., “DNAzyme‐Catalyzed Site‐Specific N‐Acylation of DNA Oligonucleotide Nucleobases,” Angewandte Chemie International Edition 63 (2024): e202317565, 10.1002/anie.202317565. 38157448 PMC10873475

[26] R. R. Poudyal , P. D. Nguyen , M. P. Lokugamage , et al., “Nucleobase Modification by an RNA Enzyme,” Nucleic acids research 45 (2017): 1345–1354, 10.1093/nar/gkw1199. 28180302 PMC5388400

[27] A. R. Gruber , R. Lorenz , S. H. Bernhart , R. Neubock , and I. L. Hofacker , “The Vienna RNA Websuite,” Nucleic acids research 36 (2008): W70–W74, 10.1093/nar/gkn188. 18424795 PMC2447809

[28] F. Li , J. Dong , X. Hu , et al., “A Covalent Approach for Site‐Specific RNA Labeling in Mammalian Cells,” Angewandte Chemie International Edition 54 (2015): 4597–4602, 10.1002/anie.201410433. 25694369

[29] S. C. Alexander , K. N. Busby , C. M. Cole , C. Y. Zhou , and N. K. Devaraj , “Site‐Specific Covalent Labeling of RNA by Enzymatic Transglycosylation,” Journal of the American Chemical Society 137 (2015): 12756–12759, 10.1021/jacs.5b07286. 26393285

[30] Y. Motorin , J. Burhenne , R. Teimer , et al., “Expanding the Chemical Scope of RNA:Methyltransferases to Site‐Specific Alkynylation of RNA for Click Labeling,” Nucleic acids research 39 (2011): 1943–1952, 10.1093/nar/gkq825. 21037259 PMC3061074

[31] C. P. M. Scheitl , M. Mieczkowski , H. Schindelin , and C. Höbartner , “Structure and Mechanism of the Methyltransferase Ribozyme MTR1,” Nature Chemical Biology 18 (2022): 547–555, 10.1038/s41589-022-00976-x. 35301481 PMC7612680

[32] J. Deng , T. J. Wilson , J. Wang , et al., “Structure and Mechanism of a Methyltransferase Ribozyme,” Nature Chemical Biology 18 (2022): 556–564, 10.1038/s41589-022-00982-z. 35301479 PMC9050513

[33] T. J. Wilson , E. McCarthy , S. Ekesan , et al., “The Role of General Acid Catalysis in the Mechanism of an Alkyl Transferase Ribozyme,” ACS Catalysis 14 (2024): 15294–15305, 10.1021/acscatal.4c04571. 39444533 PMC11494507

[34] According to Vienna RNA Fold secondary structure prediction, it may also be possible that the hairpin is (transiently) opened by the interaction with the ribozyme (Figure S4). Preliminary mutation and covariation analyses did not give fully conclusive results.

[35] R. C. Kretsch , E. S. Andersen , J. M. Bujnicki , et al., “RNA Target Highlights in CASP15: Evaluation of Predicted Models by Structure Providers,” Proteins 91 (2023): 1600–1615, 10.1002/prot.26550. 37466021 PMC10792523

