# Supplementary material for: Alkyltransferase Ribozyme for Site‐Specific N 4‐Cytidine Alkylation
Source: Angew Chem Int Ed Engl. 2026 Apr 6;65(20):e6447137. doi: 10.1002/anie.6447137 (PMC13159434; doi:10.1002/anie.6447137)
Supplement: Supplementary file 1 — Supporting File 1: Materials and Methods, Supporting Information Tables S1–S6, Supporting Information Figures S1–S10, NMR spectra. [file ANIE-65-e6447137-s001.pdf]

Supporting information for

Alkyltransferase ribozyme for site-specific N^4-cytidine-alkylation

Evgeniia Dorinova, Manisha B. Walunj, Claudia Höbartner*

correspondence: claudia.hoebartner@uni-wuerzburg.de

Institute of Organic Chemistry, Julius-Maximilians-Universität Würzburg, Am Hubland, 97074 Würzburg, Germany

Center for Nanosystems Chemistry (CNC), Julius-Maximilians-Universität Würzburg, Theodor-Boveri-Weg, 97074 Würzburg, Germany

Table of Contents

1 General materials and methods 2

2 Solid-phase RNA synthesis and deprotection 3

3 *In vitro* transcription 3

4 Labeling of oligonucleotides 3

5 *In vitro* selection 4

6 Activity of enriched RNA library and *cis*-active ribozymes 4

7 Analysis of RNA-alkylating products 5

8 Analysis of *trans*-active ribozymes 6

9 Synthesis 7

10 Supporting Information Tables 13

11 Supporting Information Figures 15

12 NMR spectra 25

References 39

1 General materials and methods

All standard chemicals and solvents were purchased from commercial suppliers and used without further purification. Dry solvents were obtained from a solvent purification system (SPS). For column chromatography, solvents purchased in technical quality were purified by distillation prior to use. Water for *in vitro* experiments was obtained from Arium®pro ultra pure water purification system. Thin layer chromatography (TLC) was performed on aluminum plates precoated with silica gel 60 F254 (Merck) and with visualization of plates under UV light at 254 nm. For column chromatography, silica gel (Kieselgel 60, Merck, 40–63 µm) was used. NMR spectra were recorded on a Bruker Avance III HD 400 spectrometer at 400 MHz. NMR signals were assigned by the help of 2D-spectroscopy (COSY, HSQC, HMBC). High resolution (HR) electrospray ionization (ESI) mass spectra (MS) were measured on Bruker micrOTOF-Q III spectrometer.

Snake venom phosphodiesterase (SVPD) was purchased from Merck. Bacterial alkaline phosphatase (BAP) and SuperScript III reverse transcriptase (SSIII), RNase T1, Calf Intestinal Alkaline Phosphatase (CIAP), Klenow exo- DNA polymerase, DreamTaq DNA-polymerase and Phusion High-Fidelity DNA-Polymerase were purchased from Thermo-Fisher Scientific. T4 Polynucleotide kinase (PNK) was purchased from New England Biolab (NEB). The T7 RNA polymerase was expressed in house according to a published protocol.[1] Dynabeads streptavidin T1 beads and speedbead neutravidin coated-magnetic particles were purchased from Thermo Fisher scientific and Cytiva respectively. Microspin G25-columns were from Cytiva. NTPs, dNTPs, ddNTPs were purchased from Jena Bioscience and γ-^{32}P-ATP from Hartmann Analytic GmbH. Lucifer Yellow-carbohydrazide, Fluorescein-thiosemicarbazide and Azide-PEG$_3$-biotin conjugate were purchased from Sigma Aldrich. Biotinylated O^6-benzylguanine (BG-biotin/SNAP-biotin) and O^6-(4-(aminomethyl)benzyl)guanine (BG-NH$_2$) were purchased from NEB, O^6-benzylguanine (Bn^{6}G) was from Alfa Aesar. All other O^6-modified guanine cofactors were synthesized in house according to literature known procedures.[2] O^6-(4-(propargyloxy)benzyl)guanine (BG-Oprop) was synthesized in house as described below.

Unmodified DNA oligonucleotides were purchased from Microsynth and purified by denaturing PAGE (10 – 20% acrylamide). Unmodified RNA substrates and ribozymes were prepared by *in vitro* transcription with T7 RNA polymerase from synthetic DNA templates.

Fluorescence gel images were recorded using a ChemiDoc MP with epi illumination using blue LEDs (emission filter 530/28) and red LEDs (emission filter 695/55), BioRad. Gels containing radioactive oligonucleotides were exposed to phosphor storage screens and imaged using an Amersham Typhoon Phosphorimager, GE Healthcare. For measurement of fluorescence, a Jasco FP-8300 spectrofluorometer was used.

2 Solid-phase RNA synthesis and deprotection

RNA oligonucleotides were prepared by solid-phase synthesis using phosphoramidite chemistry with 2'-O-TOM-protection on controlled pore glass (CPG) solid support. RNA sequences are given in Supporting information Table S2. RNA oligonucleotides were cleaved from the solid support and deprotected with aqueous ammonia : methylamine solution = 1:1 (AMA) at 37 °C for 5 h, followed by 1 M tetrabutylammonium fluoride in anhydrous THF overnight at 37 °C, desalted and purified by denaturing PAGE. The quality of synthesized RNA oligonucleotides was analyzed by anion-exchange HPLC (Dionex DNAPac PA200, 2 × 250 mm, at 60 °C; solvent A: 25 mM Tris-HCl (pH 8.0) and 6 M urea; solvent B: 25 mM Tris-HCl (pH 8.0), 6 M urea and 0.5 M $NaClO_4$ with the gradient: linear 0–40% solvent B, with a slope of 4% solvent B per column volume, flow rate 0.5 ml min^{-1}, UV detection at 260 nm) and HR-ESI-MS (micrOTOF-Q III, negative-mode, direct injection). Measured and calculated masses are listed in Supporting Information Table S6.

3 *In vitro* transcription

In vitro transcription was performed in a total volume of 100 µL using 1 µM synthetic DNA templates, 4 mM NTPs, 40 mM Tris-HCl (pH 8.0), 30 mM $MgCl_2$, 2 mM spermidine, 10 mM DTT and 0.1 mg T7 RNA polymerase expressed in house[1]. Reactions were incubated at 37 °C for 5 h or overnight, quenched with 0.5 M EDTA (pH 8.0), and transcripts were purified by denaturing PAGE. The sequences of the transcripts are summarized in Supporting Information Table S3, Table S4.

4 Labeling of oligonucleotides

4.1 3'-Labeling of RNA using periodate glycol oxidation

For labeling of substrate RNA, 3-5 nmol was dissolved in a freshly prepared sodium phosphate buffer (250 mM, pH 7.4), mixed with a freshly prepared aqueous solution of $NaIO_4$ (20 mM final concentration) in a total volume of 40 µL and incubated at 37 °C for 10 min. A freshly prepared aqueous solution of Na_2SO_3 was added to a final concentration of 83 mM followed by the incubation for 5 min at 37 °C. Subsequently, 100 mM fluorescein-5-thiosemicarbazide solution in DMSO was added to a final concentration of 8.3 mM, and the reaction mixture was incubated for 1 h at 37 °C in the dark. The labeled RNA was purified on 20 % denaturing PAGE.

For the labeling step during *in vitro* selection cycle, 200 pmol of RNA library was labeled with Lucifer yellow carbohydrazide in a total volume of 12 µL in analogy to mentioned above, and the labeled RNA library was purified using Microspin G25-columns following the manufacturer's protocol.

4.2 5'-^{32}P-labeling of oligonucleotides

100-200 pmol of an oligonucleotide in 1 × PNK buffer A (50 mM Tris, pH 7.6, 10 mM $MgCl_2$, 5 mM DTT, 0.1 mM spermidine) was treated with 5 µCi *γ*-^{32}P-ATP and 5 U of T4 PNK in a total reaction volume of 10 µL. The reaction was incubated for 1 h at 37 °C. The labeled DNA was recovered by precipitation with EtOH to remove the excess of γ-^{32}P-ATP. In case of RNA, the reaction was quenched with loading dye and purified on 15 % or 20 % denaturing PAGE.

For RNA transcripts, a free 5’ OH was first generated via dephosphorylation with CIAP. 200 pmol of transcripts were dissolved in 10 µL of 1 × CIAP buffer (50 mM Tris-HCl, pH 8.5, 0.1 mM EDTA) and subjected to the reaction with 1 U CIAP for 1 h at 37 C followed by the extraction with phenol-chloroform-isoamyl alcohol (PCI) and EtOH precipitation.

5 *In vitro* selection

The *in vitro* selection was performed as previously published.[3] The dsDNA template for *in vitro* transcription of the initial RNA library was prepared by overlapping primer extension using two DNA oligonucleotides (D_2+D_3, N_{40}: A:C:G:T = 1:1:1:1) and Klenow fragment exo$^-$ DNA polymerase. 100 pmol of dsDNA template was used for *in vitro* transcription with T7 RNA polymerase in a final volume of 100 µL. For the first selection round, 400 pmol RNA pool (containing 25% 3'-fluorescently labeled RNA obtained by sodium periodate oxidation and labeling with Lucifer yellow carbohydrazide according to the ref.[4]) was annealed in 1 × selection buffer (120 mM KCl, 5 mM NaCl and 50 mM HEPES, pH 7.5; 3 min at 95 °C, and then 10 min at 25 °C) and subjected to incubation with 100 µM of the biotinylated *O*^{6}-benzylguanine (BG–biotin) cofactor (SNAP–biotin ®) and 40 mM $MgCl_2$ at 37 °C for 14 h.

In subsequent rounds, 200 pmol of RNA in 12 µL reaction volume was used for the selection step. The separation of active biotinylated RNA from inactive species (capture, washing and elution steps) was performed according to commercial protocols, followed by reverse transcription using SuperScript™ III reverse transcriptase and PCR amplification with Phusion™ High-Fidelity DNA Polymerase or DreamTaq™ DNA Polymerase as established previously.[5,6] The enriched RNA library for the next selection round was generated by *in vitro* transcription in a total volume of 100 µL as described above and purified via denaturing PAGE. Overall, eight rounds of *in vitro* selection were performed, afterward the RNA pool was cloned into *E.coli* via TOPO TA cloning, and eight randomly picked clones were subjected to Sanger sequencing.

6 Activity of enriched RNA library and *cis*-active ribozymes

6.1 Ribozyme-catalyzed RNA alkylation reaction

In a total volume of 10 µL, 100 pmol of the *cis*- or bimolecular constructs (RNA substrate : ribozyme = 1:1) were annealed (3 min at 95 °C, 10 min at 25 °C) in 1 × reaction buffer (5 mM NaCl, 120 mM KCl, 50 mM HEPES pH 7.5) or Bis-Tris pH 6.0) and incubated with a cofactor and $MgCl_2$ at final concentrations of 100 µM and 40 mM respectively. The reactions were kept at 37 °C for 18 h. Modified RNA products were recovered by ethanol precipitation and used without further purification for the next steps.

6.2 RNA functionalization using copper(I)-catalyzed azide-alkyne cycloaddition (CuAAC)

Modification of RNA was performed as described above in a total reaction volume of 10 µL in 1 × reaction buffer and *O*^{6}-(4-(propargyloxymethyl)benzyl)guanine (BG-prop, **2**) or BG-Oprop (**7**) as cofactors.

In a total volume of 10 µL, 50 – 100 pmol of RNA modified with a cofactor carrying a terminal alkyne group was mixed with 1 µL of 5 mM a fluorophore or biotin-PEG_3-azide in DMSO:^{t}BuOH (3:1). Subsequently, 1 µL of freshly prepared CuBr solution (10 mM in DMSO:^{t}BuOH (3:1)) was premixed with 2 µL of TBTA (10 mM in DMSO:^{t}BuOH (3:1)) and added to the reaction mixture. After incubation at 37 °C for 3 h in the dark, the reaction mixture was precipitated twice with EtOH and either coupled

with streptavidin for the PAGE analysis or used directly for the downstream purposes. For a large scale click-labeling reaction, 1 – 5 nmol of RNA modified with a cofactor carrying a terminal alkyne group in a 20 µL of the reaction mixture was used, and the labeled RNA-oligonucleotides were purified on 20% denaturing PAGE.

6.3 Streptavidin gel-shift assay

To investigate the *cis*-activity of the enriched RNA library or selected individual clones, 75 pmol of RNA was subjected to self-modification reaction in a total volume of 5 µL in the presence of 100 µM BG-biotin or DMSO (negative controls) and 40 mM $MgCl_2$ in 1 × reaction buffer (120 mM KCl, 5 mM NaCl, 50 mM HEPES pH 7.5 or Bis-Tris, pH 6.0). The annealing step was performed prior to the addition of the cofactor and $MgCl_2$ as described above to ensure the proper folding of RNA, and the reaction was incubated at 37°C for 23 h. For the activity tests of bimolecular constructs incubated with BG-prop, CuAAC reaction was performed as described in 6.2 before coupling to streptavidin. After the reaction products were recovered by ethanol precipitation, 3 pmol of RNA was mixed with 1 µL of 1 mg/mL streptavidin in 1 × TBS buffer (150 mM NaCl, 50 mM Tris-HCl, pH = 7.5) at room temperature for 10 min. As negative references, reacted RNA was incubated in the absence of streptavidin. 1 µL of loading dye was added, and the reactions were resolved on 10% native PAGE, stained with SYBR-Gold and visualized under UV trans illumination. PAGE images of activity assays using streptavidin gel-shift assay are shown in Supporting Information Figure S2.

7 Analysis of RNA-alkylating products

7.1 Ribozyme-catalyzed RNA alkylation on a preparative scale

In a total volume of 20 µL, 1 eq. of the substrate RNA (R1-R3, 50 – 100 µM) was incubated with 1.1 eq. of the ribozyme (Rz5 or Rz6, 55 – 110 µM) using BG-prop, BG-Oprop or Bn^6G (100 µM) and 40 mM $MgCl_2$ in 1 × reaction buffer (5 mM NaCl, 120 mM KCl, 50 mM Bis-Tris pH 6.0) at 37 °C for 18 h. Modified RNA products were purified by 15% denaturing PAGE and analyzed by anion-exchange HPLC (Dionex DNAPac PA200, 2 × 250 mm, at 60 °C; solvent A: 25 mM Tris-HCl (pH 8.0) and 6 M urea; solvent B: 25 mM Tris-HCl (pH 8.0), 6 M urea and 0.5 M $NaClO_4$ with the gradient: linear 0 – 40% solvent B, with a slope of 4% solvent B per column volume, flow rate 0.5 mL min^{-1} at 60 °C, UV detection at 260 nm).

7.2 Enzymatic digestion and LC-MS-analysis

For LC-MS analysis, 500 pmol unmodified and modified RNA were digested with 12.0 U of BAP and 1.0 U of SVPD in the reaction buffer (40 mM Tris-HCl, 20 mM $MgCl_2$, pH 7.5) for 18 h at 37 °C. 200 pmol of each synthetic standards (N^3-benzyl-cytidine, N^4-benzylcytidine and *2'O*-benzylcytidine were used as references. After extracting the digested nucleoside mixture with 100 µL of chloroform, the aqueous layer was concentrated by lyophilization, and the residue was dissolved in 70 µL of 10 mM NH_4OAc (pH 5.3) and analyzed by LC-MS, using a Synergi Fusion RP column (Phenomenex, 4 µm, 250 × 2 mm). The analysis was run with a gradient of 0–5% (0–15 min) and 5–72.5% (15–45 min) of solvent B. Solvent A: 10 mM NH_4OAc (pH 5.3), solvent B: MeCN. The flow rate was 0.2 mL min^{-1} at 25 °C with UV detection at 260 nm and online MS in a microTOF-Q III system in positive-ion mode. Data were analyzed with Data Analysis software DA 4.2 (Bruker Daltonics) and plotted using OriginPro (2023b).

7.3 RNAse T1 digestion and alkaline hydrolysis

For the digestion by RNAse T1, 75 IPS of 5'-^{32}P-labeled unmodified and modified RNA were treated with 0.5 U of RNase T1 in 5 µL of the reaction volume (50 mM Tris-HCl, pH 7.5) for 25 s at 37 °C. For alkaline hydrolysis, 250 IPS of 5'-^{32}P-RNA was digested in 10 µl of alkaline hydrolysis solution (20 mM NaOH) for 4 min at 95 °C. The reactions were quenched immediately by addition of loading dye, stored on ice, resolved on 15% denaturing PAGE and visualized by autoradiography.

7.4 Reverse transcription stop assay

Reverse transcription was performed to analyze the activity of *cis*-reacting ribozyme and determine the position of the modification site by the ribozyme. 10 pmol the *cis*-active ribozyme was first annealed (3 min at 95 °C, 10 min at 25 °C) in 1 × reaction buffer (120 mM KCl, 5 mM NaCl, 50 mM HEPES, pH 7.5) in a final volume of 10 µL and incubated with 100 µM BG-biotin cofactor and 40 mM $MgCl_2$ at 37°C for 18-23 h, followed by EtOH precipitation.

For RT primer extension reaction, 10 pmol of the modified and unmodified RNA and 100 IPS of the corresponding 5'-^{32}P-labeled primer were annealed in the annealing buffer (5 mM Tris-HCl pH 7.5, 0.1 mM EDTA), cooled down to the room temperature followed by the addition of 5 mM DTT, 0.5 mM of each dNTP and 50 U of SuperScript III RT in 10 µL of 1 × first strand buffer (50 mM Tris-HCl, pH 8.3, 75 mM KCl, 3 mM $MgCl_2$). The reaction mixture was incubated at 55 °C for 1 h, quenched with 1 µL 2 N NaOH and incubated for 5 min at 95 °C to degrade the RNA template. Following precipitation with EtOH, the reaction products were dissolved in loading dye, analyzed on denaturing PAGE and visualized by autoradiography. For sequencing ladders, unmodified RNA and suitable dNTP/ddNTP mixtures (0.5 mM ddNTP, 0.05 mM corresponding dNTP and 0.5 mM each of the other three dNTPs) instead of an equal mix of dNTPs were used.

7.5 DNA-catalyzed ribozyme cleavage using 8-17 DNAzymes

To assess the localization of the modification site, *cis*- (Rz2) and bimolecular constructs (Tr1+Rz3, Tr2+Rz4) of the ribozyme were subjected to cleavage reactions by 8-17 DNAzymes. First, ribozymes in *cis*- or bimolecular setup were modified with BG-prop or BG-Oprop and coupled with sulfo-Cyanine5 azide ($Cy5N_3$) as described above. The reaction products were recovered by ethanol precipitation and used directly for the cleavage reaction with DNAzymes.

In a total volume of 10 µL, 10 pmol of each reacted ribozyme constructs were mixed in 1 × reaction buffer (50 mM HEPES, pH 7.5, 400 KCl, 100 mM NaCl) with 100 pmol of the corresponding 8-17 DNAzymes designed to cut a specific NG junction within the catalytic core or the connecting loop of the ribozyme[7]. After the annealing step was performed (3 min at 95 °C, 10 min at 25 °C), 10 mM $MgCl_2$ and 10 mM $MnCl_2$ were added, and reactions were incubated at 37 °C for 16 h, quenched with 10 µL of stop solution, and aliquots corresponding to 1 pmol of each reaction were resolved on 15% denaturing PAGE, stained with SYBR-Gold and subjected to fluorescence imaging.

8 Analysis of *trans*-active ribozymes

8.1 Kinetic assays

Kinetic assays were performed under single turnover conditions in 10 µL of 1 × reaction buffer (5 mM NaCl, 120 mM KCl, 50 mM Bis-Tris pH 6.0 or HEPES pH 7.5) at 37 °C using 500 IPS of a ^{32}P-labeled- or 10 pmol of 3'-fluoresently labeled substrate RNA (Tr3-Tr6) and 100 pmol of the corresponding ribozyme (Rz6). An annealing step (3 min at 95 °C, 10 min at

25 °C) was performed to ensure proper binding of the ribozyme with the substrate RNA followed by the addition of 100 µM cofactor and 40 mM $MgCl_2$. An aliquot of 1 µL of the reaction was taken out at a certain time point and quenched immediately by the addition of 4 µL loading dye. The time points of the reactions were resolved on 15% denaturing PAGE and visualized either by autoradiography or using blue epi illumination and a 530/28 nm emission filter respectively. For studying of the Mg^{2+-} dependency, 100 µM cofactor (BG-Oprop) at pH 6.0 and $MgCl_2$ at final concentration of 5 mM, 10 mM, 20 mM, 40 mM were used. The fraction modified was plotted versus time data and fit to pseudo-first order kinetics $Y = Y_{max}(1 - e^{- kobs*t})$ to obtain the observed reaction rate k_{obs} and the final yield Y_{max} using OriginPro (2023b).

8.2 Analysis via anion-exchange HPLC

For kinetic assay analyzed by anion-exchange HPLC, 100 pmol of a substrate RNA (R1 or R2) was annealed (3 min at 95 °C, 10 min at 25 °C) with 110 pmol of the corresponding ribozyme (Rz5 or Rz6) in 5 µL of 1 × reaction buffer (5 mM NaCl, 120 mM KCl, 50 mM Bis-TrispH 6.0). Afterward, a cofactor and $MgCl_2$ were added to final concentration of 100 µM and 40 mM respectively, and incubated at 37 °C. The reactions corresponding to time points 0 and 19 h were quenched with 0.5 M EDTA (pH 8.0) and analyzed by anion-exchange HPLC (Dionex DNAPac PA200, 2 × 250 mm, at 60 °C; solvent A: 25 mM Tris-HCl (pH 8.0) and 6 M urea; solvent B: 25 mM Tris-HCl (pH 8.0), 6 M urea and 0.5 M $NaClO_4$ with the gradient: linear 0–40% solvent B, with a slope of 4% solvent B per column volume, flow rate 0.5 mL min−1 at 60 °C, UV detection at 260 nm).

9 Synthesis

9.1 Synthesis of BG-Oprop (7)

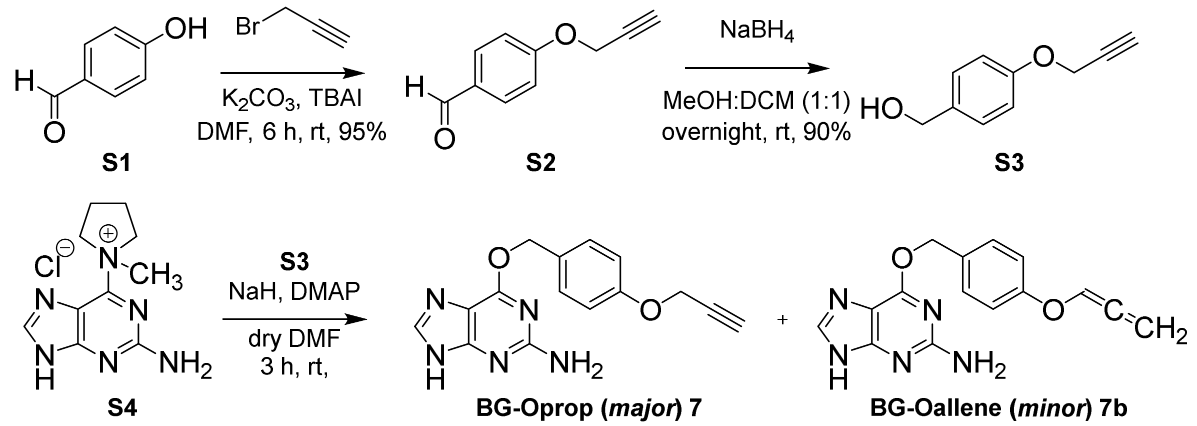

4-(prop-2-yn-1-yloxy)benzaldehyde (S2): Potassium carbonate (2.31 g, 16.72 mmol, 2.04 eq.) and tetrabutylammoniumiodide (TBAI, 3.77 mg) were added to 4-(hydroxy)-benzyladehyde **S1** (1.00 g**,** 8.19 mmol, 1.00 eq.) in dry DMF (5 mL). Further, propargyl bromide (0.95 mL, 12.30 mmol, 1.50 eq.) was added to the above solution, and the reaction mixture was stirred at room temperature for 6 h. 50 mL of distilled water was added to the reaction, and the aqueous layer was washed with diethyl ether (2 × 50 mL). The combined organic layers were dried over anhydrous Na_2SO_4, and the solvent was removed in vacuo to furnish product **S2** as a colorless oil (1.44 g, 95%). Analytical data are in agreement with the previous report.[8]

TLC (EtOAc/n-hexane = 50:50); $R_{f\,=}$ 0.82.

^{1}H NMR (400 MHz, chloroform-*d*) δ (ppm) = 9.90 (s, 1H, -CHO), 7.88 – 7.84 (m, 2H, Ar-H), 7.11 – 7.07 (m, 2H, Ar-H), 4.78 (d, *J* = 2.4 Hz, 2H, -OCH_2), 2.57 (t, *J* = 2.4 Hz, 1H, -C≡C-H).

^{13}C {^{1}H} NMR (101 MHz, chloroform-*d*) δ (ppm) = 190.94 (-CHO), 162.49 (Ar-C), 132.04 (2C, Ar- C), 130.71 (Ar-C), 115.30 (2C, Ar-C), 77.66 (C≡C-H), 76.51 (C≡C-H), 56.07 (OCH_2).

(4-(prop-2-yn-1-yloxy)phenyl)methanol (S3): Sodium borohydride (586 mg, 15.48 mmol, 2.00 eq.) was added to the solution of 4-(prop-2-yn-1-yloxy)benzaldehyde **S2** (1.24 g, 7.74 mmol, 1.00 eq.) in equal amount of MeOH and DCM (110 mL) at 0°C. The reaction mixture was warmed to room temperature and stirred overnight. After completion, the crude reaction mixture was quenched with 150 mL of water, and the aqueous layer was extracted with diethyl ether (2 × 150 mL). Further, the organic layer was dried over anhydrous Na_2SO_4 and concentrated under vacuum to afford pure product **S3** as a colorless oil (1.12 g, 90%). Analytical data are in agreement with the previous report.[8]

TLC (EtOAc/n-hexane = 50:50); $R_{f\,=}$ 0.52.

^{1}H NMR (400 MHz, chloroform-*d*) δ (ppm) = 7.32 – 7.29 (m, 2H, Ar-H), 6.99 – 6.95 (m, 2H, Ar-H), 4.69 (d, *J* = 2.4 Hz, 2H, -OCH_2-C≡C), 4.62 (s, -CH_2OH), 2.52 (t, *J* = 2.4 Hz, 1H, -C≡C-H).

^{13}C {^{1}H} NMR (101 MHz, chloroform-*d*): δ (ppm) = 157.22 (Ar-C), 134.16 (Ar-C), 128.73 (2C, Ar-C), 115.10 (2C, Ar-C), 78.61 (C≡C-H), 75.70, C≡C-H), 65.07(-CH_2OH), 55.95 (-OCH_2).

6-((4-(prop-2-yn-1-yloxy)benzyl)oxy)-9H-purin-2-amine (BG-Oprop, 7)

(4-(prop-2-yn-1-yloxy)phenyl)methanol (**S3**, 192 mg, 1,18 mmol, 1.50 eq.) was dissolved in 3 mL of dry DMF. Sodium hydride (60% dispersion in mineral oil) (75.84 mg, 3.16 mmol, 4.00 eq.) was added in portions to the above solution over 5 min. Afterwards, the reaction mixture was stirred for 10-15 min at 0 °C. 1-(2-Amino-9H-purin-6-yl)methylpyrrolidin-1-ium chloride (**S4**, 200 mg, 0.79 mmol, 1.00 eq.) and 4-dimethylaminopyridine (19.3 mg, 0.16 mmol, 0.25 eq.) were added. Further, the reaction mixture was stirred at room temperature for 3 h. Distilled water was added to quench the excess sodium hydride. Solvents were removed under reduced pressure, and the crude mixture was purified by silica gel column chromatography using gradient based on MeOH:CH_2Cl_2 (5 % MeOH) to afford 6-((4-(prop-2-yn-1-yloxy)benzyl)oxy)-9H-purin-2-amine (BG-Oprop, **7**) as a white solid (167 mg, 72%), containing 14% of a byproduct, BG-Oallene **7b** (as determined by integration of the allene H-18 and alkyne H-20 protons in the ^{1}H NMR spectrum).[8] Both compounds exhibit identical R_f values and could not be separated by silica gel column chromatography.

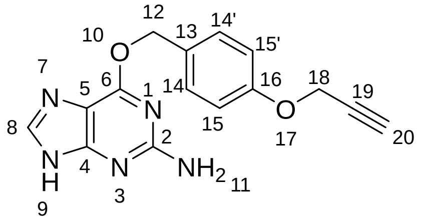

BG-Oprop

TLC (MeOH/CH_2Cl_2 = 10:90); $R_{f\,=}$ 0.58.

^{1}H NMR (400 MHz, DMSO-d_6) δ (ppm) = 12.42 (s, 1 H, H-9), 7.80 (s, 1H, H-8), 7.49 – 7.46 (m, 2H, H-14, H-14'), 7.02 – 6.98 (m, 2H, H-15, H-15'), 6.31 (s, 2H, H-11), 5.40 (s, 2H, H-12), 4.80 (d, *J* = 2.4 Hz, 2H, H-18), 3.56 (t, *J* = 2.4 Hz, H-20).

^{13}C {^{1}H} NMR (101 MHz, DMSO-d_6): δ (ppm) = 159.81 (C-6), 159.57 (C-2), 156.97 (C-16), 156.37 (C-4), 137.67 (C-8), 130.31 (2C, C-14, C-14'), 129.38 (C-13), 114.64 (2C, C-15, C-15'), 113.41 (C-5), 79.13 (C-19), 78.20 (C-20), 66.36 (C-12), 55.31 (C-18).

HR-MS (ESI$^+$): Exact mass calculated for $C_{15}H_{13}N_5NaO_2$ $[M+Na]^+$: 318.09615, found: 318.09566.

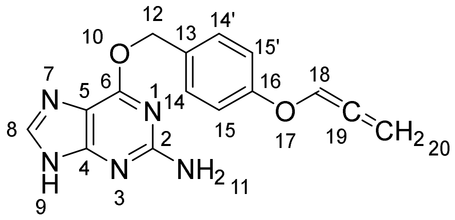

BG-Oallene

TLC (MeOH/CH_2Cl_2 = 10:90); R_f = 0.58.

^{1}H NMR (400 MHz, DMSO-d_6) δ (ppm) = 12.42 (s, 1 H, H-9), 7.80 (s, 1H, H-8), 7.51– 7.49 (m, 2H, H-14, H-14'), 7.23 (t, J = 6 Hz, 1H, H-18), 7.12 – 7.10 (m, 2H, H-15, H-15'), 6.31 (s, 2H, H-11), 5.58 (d, J = 6 Hz, 2H, H-20), 5.43 (s, 2H, H-12).

^{13}C {^{1}H} NMR (101 MHz, DMSO-d_6): δ (ppm) = 201.95 (C-19), 159.81 (C-6), 159.57 (C-2), 155.06 (C-16), 137.67 (C-8), 130.31 (2C, C-14, C-14'), 129.38 (C-13), 117.54 (C-18), 116.24 (2C, C-15, C-15'), 113.41 (C-5), 90.41 (C-20), 66.36 (C-12).

HR-MS (ESI$^+$): Exact mass calculated for $C_{15}H_{13}N_5NaO_2$ $[M+Na]^+$: 318.09615, found: 318.09566.

9.2 Synthesis of synthetic standards

9.2.1 *N*^{4}-benzylcytidine (*N*^{4}BnC)

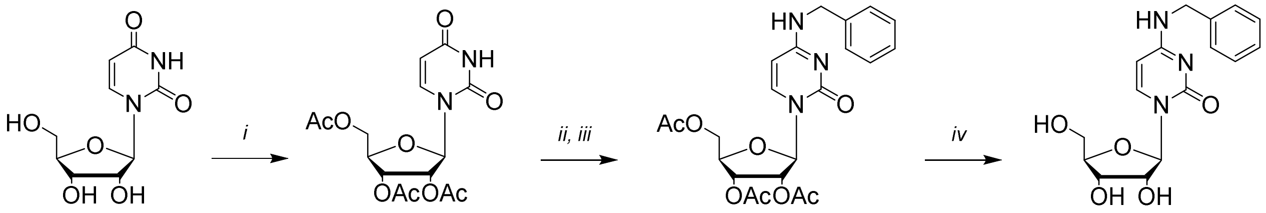

S5 S6 S7 *N*^{4}BnC

Scheme S1. *i*) acetic anhydride, DMAP in pyridine, 18 h, r.t, 81%. *ii*) 2,4,6-triisopropylbenzensulfonyl chloride, DMAP, Et_3N in 1,2-dichloroethane, 2 h, r.t, 27%. *iii*) benzylamine, Et_3N in ethanol, 17.5 h, r.t. *iv*) 33% w/w methylamine in ethanol, 17 h, r.t..

2', 3', 5′-tri-*O*-acetyluridine (S6): Starting from uridine (**S5**), compound **S6** was synthesized according to the published procedure.[9] Analytical data are in agreement with the previous report.[10]

***N*^{4}-benzylcytidine (*N*^{4}BnC)**: To 75.2 mg of 2',3'-5'-tri-*O*-acetyluridine (**S6**, 203 µmol, 1.00 eq.) in 1,2-dichloroethane (5 mL), DMAP (2.47 mg, 20.3 µmol, 0.10 eq.) and triethylamine (283 µL, 2.03 mmol, 10.00 eq.) were added, and the reaction mixture was allowed to stir at room temperature under N_2 atmosphere for 10 min. Afterwards, 2, 4, 6-triisopropylbenzensulfonyl chloride (92.4 mg, 304.5 µmol, 1.50 eq.) was added, and the reaction mixture was stirred at room temperature for additional 2 h. The reaction mixture was diluted with CH_2Cl_2 and extracted with sat. aq. $NaHCO_3$ solution (3 × 10 mL). The organic layer was collected, dried over anhydrous Na_2SO_4 and filtered off. The filtrate was evaporated, and the residue was dried

under high vacuum. The crude product triisopropylbenzensulfonyl-activated **S6** was used for the next step without purification. The crude compound (72.0 mg, 113 µmol, 1.00 eq.) was resuspend in ethanol (5 mL). Triethylamine (10.8 µL, 78.0 µmol, 0.70 eq.) and benzylamine (17.1 µL, 157 µmol, 1.40 eq.) were added, and the reaction mixture was stirred at room temperature under N_2 atmosphere for 17.5 h. The crude product was purified by silica gel column chromatography (CH_2Cl_2:MeOH = 97.5 : 2.5 to CH_2Cl_2:MeOH = 95 : 5) to yield the compound **S7** (15.3 mg, 27%) as a white solid (R_f=0.57, CH_2Cl_2:MeOH = 5 : 1). **S7** was resuspended in 33% (w/w) methylamine solution in ethanol (5 mL) and stirred at room temperature for 17 h. The solvent was evaporated, and the crude product was purified by silica gel column chromatography (CH_2Cl_2:MeOH = 85 : 15) to afford the desired product ***N*^{4}BnC** as a white powder (15.3 mg) in a quantitative yield.

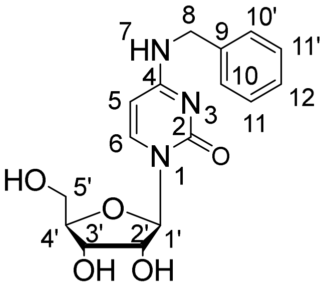

***N*^{4}BnC**

^{1}H NMR (400 MHz, DMSO-*d6*): δ (ppm) = 8.16 (t, *J* = 5.8 Hz, 1H, H-7), 7.85 (d, *J* = 7.4 Hz, 1H, H-6), 7.38 – 7.23 (m, 5H, H-9, H-10, H-10', H-11, H-11'), 5.81 (d, *J* = 7.4 Hz, 1H, H-5), 5.77 (d, *J* = 3.8 Hz, 1H, H-1'), 5.31 (d, *J* = 5.3 Hz, 1H, 2'-OH), 5.05 (dd, *J* = 5.2 Hz, 1H, 5'-OH), 4.98 (d, *J* = 5.3 Hz, 1H, 3'-OH), 4.50 (d, *J* = 5.8 Hz, 2H, H-8), 3.98 – 3.90 (m, 2H, H-2', H-3'), 3.85 – 3.79 (m, 1H, H-4'), 3.70 – 3.61 (m, 1H, H-5'a), 3.59 – 3.50 (m, 1H, H-5'b).

^{13}C {^{1}H} NMR (100 MHz, DMSO-*d6*): δ (ppm) = 163.79 (C-4), 155.77 (C-2), 141.11 (C-6), 139.46 (C-9), 128.84 (2C, C-11, C-11'), 127.88 (2C, C-10, C-10'), 127.39 (C-12), 94.86 (C-5), 89.62 (C-1'), 84.49 (C-4'), 74.42 (C-2'), 69.87 (C-3'), 61.07 (C-5'), 43.54 (C-8).

HR-MS (ESI$^+$): Exact mass calculated for $C_{16}H_{19}N_3NaO_5$ [M+Na]$^+$: 356.12169, found: 356.12079.

9.2.2 *2'O*-benzylcytidine (2'*O*BnC)

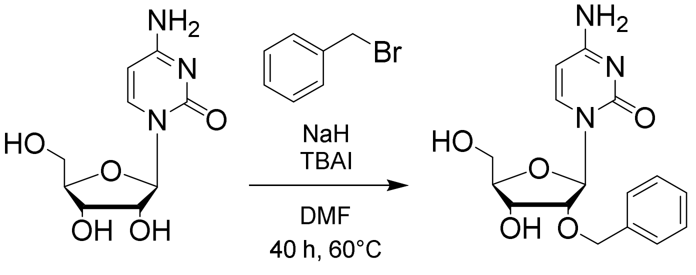

S8 **2'*O*BnC**

Starting from cytidine (**S8**), **2'*O*BnC** was synthesized in analogy to the published procedures[11].

200 mg of **S8** (824 µmol, 1.00 eq.) was suspended in anhydrous DMF (800 µL) while heating to 50 °C followed by cooling down the reaction on an ice bath. NaH (60% dispersion in mineral oil: 39.9 mg, 998 µmol, 1.21 eq.) was added, and the mixture was stirred on ice until no further gas evolution was observed. 66.5 mg of TBAI (180 µmol, 0.22 eq.) and benzyl

bromide (107 µL, 1.10 eq.) were added, and the mixture was stirred at 60 °C for 39 h. The solvent was removed under reduced pressure, and the crude product was purified on silica gel column chromatography using 10% MeOH in CH_2Cl_2 to yield the desired product **2'*O*BnC** (28.4 mg, 10%) as a yellow powder.

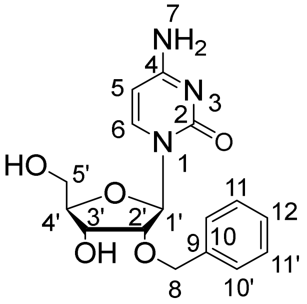

2'*O*BnC

^{1}H NMR (400 MHz, DMSO-*d6*): δ (ppm) = 7.89 (d, *J* = 7.4 Hz, 1H, H-6), 7.38 – 7.26 (m, 5H, H-9, H-10, H-10', H-11, H-11'), 7.20 (br s, 1H, H-7), 7.15 (br s, 1H, H-7), 5.94 (d, *J* = 3.6 Hz, 1H, H-1'), 5.69 (d, *J* = 7.4 Hz, 1H, H-5), 5.14 (d, *J* = 6.4 Hz, 1H, 3'-OH), 5.11 (dd, *J* = 5.1 Hz, 1H, 5'-OH), 4.73 (d, *J* = -12.2 Hz, 1H, H-8a), 4.68 (d, *J* = -12.2 Hz, 1H, H-8b), 4.12 – 4.05 (m, 1H, H-3'), 3.89 (dt, *J* = 6.1 Hz, 2.9 Hz, 1H, H-4'), 3.83 (dd, *J* = 5.0 Hz, 3.6 Hz, 1H, H-2'), 3.70 (ddd, *J* = 12.2 Hz, 5.1 Hz, 2.9 Hz, 1H, H-5'a), 3.58 (ddd, *J* = 12.2 Hz, 5.1 Hz, 3.1 Hz, 1H, H-5'b).

^{13}C {^{1}H} NMR (101 MHz, DMSO-*d6*): δ (ppm) = 166.06 (C-4), 155.58 (C-2), 141.46 (C-6), 138.81 (C-9), 128.61 (C-11, C-11'), 127.86 (C-10, C-10'), 127.82 (C-12), 94.31 (C-5), 87.88 (C-1'), 84.58 (C- 4'), 81.78 (C-2'), 71.22 (C-8), 68.45 (C-3'), 60.50 (C-5').

HR-MS (ESI$^+$): Exact mass calculated for $C_{16}H_{20}N_3O_5$ $[M+H]^+$: 334.13975, found: 334.13899, calculated for $C_{16}H_{19}N_3NaO_5$ $[M+Na]^+$: 356.12169, found: 356.12129.

9.2.3 *N*^{3}-benzylcytidine (*N*^{3}BnC)

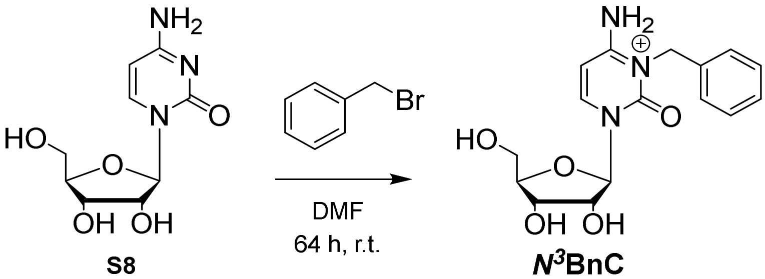

Starting from cytidine (**S8**), ***N*^{3}BnC** was synthesized in analogy to the published procedures[12].

To 101.6 mg of **S8** (418 µmol, 1.00 eq.) dissolved in anhydrous DMF (2 mL), benzyl bromide (97.8 µL, 822 µmol, 1.97 eq.) was added. The reaction mixture was kept stirring at room temperature for 19.5 h. The crude product was purified by silica gel column chromatography using 15% MeOH in CH_2Cl_2 to yield the desired compound ***N*^{3}BnC** (38.9 mg, 28%) as a yellowish powder.

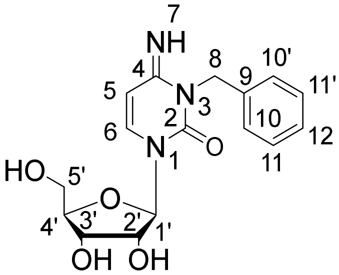

***N*[3]BnC**

^{1}H NMR (400 MHz, DMSO-*d6*): δ (ppm) = 7.97 (d, *J* = 8.0 Hz, 1H, H-6), 7.41 – 7.22 (m, 5H, H-9, H-10, H-10', H-11, H-11'), 6.13 (d, *J* = 8.2 Hz, 1H, H-5), 5.74 (d, *J* = 4.2 Hz, 1H, H-1'), 5.48 (d, *J* = 5.5 Hz, 1H, 2'-OH), 5.22 – 5.13 (m, 3H, 5'-OH, 8), 5.12 (d, *J* = 5.6 Hz, 1H, 3'-OH), 4.08 – 3.98 (m, 1H, H-2'), 3.97 (q, *J* = 5.5 Hz, 1H, H-3'), 3.87 (dt, *J* = 5.5 Hz, 3.0 Hz, 1H, H-4'), 3.69 (ddd, *J* = 12.2 Hz, 5.2 Hz, 3.0 Hz, 1H, H-5'a), 3.58 (ddd, *J* = 12.2 Hz, 5.1 Hz, 3.2 Hz, 1H, H-5'b).

^{13}C {^{1}H} NMR (101 MHz, DMSO-*d6*): δ (ppm) = 158.33 (C-4), 149.47 (C-2), 138.18 (C-6), 135.72 (C-9), 128.88 (C-11, C-11'), 127.83 (C-12), 127.51 (C-10, C-10'), 97.77 (C-5), 90.11 (C-1'), 85.07 (C-4'), 74.04 (C-2'), 69.57(C-3'), 60.69 (C-5'), 45.77 (C-8).

HR-MS (ESI^{+}): Exact mass calculated for $C_{16}H_{20}N_3O_5$ [M+H]$^{+}$: 334.13975, found: 334.13835, calculated for $C_{16}H_{19}N_3NaO_5$ [M+Na]$^{+}$: 356.12169, found: 356.12068.

10 Supporting Information Tables

Supporting Information Table S1. DNA sequences (prepared by solid-phase synthesis).

No	5'-Sequence-3'	Description
D1	CTGTAATACGACTCACTATA	T7 promotor
D2	GGTAAGGTGGACATACTG-N40-GCCTTCAAGGATGGTAGGCTGG	Rv primer of the N40 pool
D3	CTGTAATACGACTCACTATAGGACATACTGAGCCTTCAACCAGCCTAC-CATCC	Fw primer of N40 pool
D4	CTTCAACCAGCCTACCATCC	Fw primer 1PCR
D5	GGTAAGGTGGACATACTG	Rv primer RT&1st and 2nd PCR (P2)
D6	TAAATAAAATAACTGTAATACGACTCACTATAGGACATACTGAGC	Fw primer for cloning
D7	CAAGGATGGTAGGCTGGT	P1 – Primer for RT
D8	AAGGATGGTAGGTGTCAGCGACTCGAAGGTTGAAGGC	DNAzyme 1 (Dz1)
D9	ACTGCCAATGTATGTCAGCGACTCGAAAGCATCAGGG	DNAzyme 2 (Dz2)
D10	ATGTACGAGCATTGTCAGCGACTCGAAGGGGACGGCG	DNAzyme 3 (Dz3)

Supporting Information Table S2. RNA sequences (prepared by solid-phase synthesis). Modification site is depicted in blue, mutations are shown in purple, binding arms are underlined.

No	5'-Sequence-3'	Description
R1	GAUGCUCGUACAUUGGCGAUACC	Parent RNA (23) engineered in trans
R2	GAUGCUCGUACAUUGGCCUUCC-NH_2	Parent RNA (22) 3'-NH_2

Supporting Information Table S3. RNA sequences (prepared by *in vitro* transcription). Modification site is depicted in blue, mutations are shown in red.

No	5'-Sequence-3'	Description
Tr1	GGACAUACUGAGCCUUCAA	Parent RNA (19)
Tr2	GGUAUCGAGUCUCC	Parent RNA (14) mutated binding arms
Tr3	GAUGCUCGUACAUUGGCCUUCC	Parent RNA (22) WT
Tr4	GGAUGCUAGUACAUUGGCCUUCC	Sub seq C8A
Tr5	GGAUGCUGGUACAUUGGCCUUCC	Sub seq C8G
Tr6	GGAUGCUUGUACAUUGGCCUUCC	Sub seq C8U

Supporting Information Table S4. Ribozymes (prepared by *in-vitro* transcription). Binding arms are underlined, connecting loop is depicted in yellow, sub seq is shown in grey).

No	5'-Sequence-3'	Description
Pool	GGACAUACUGAGCCUUCAACCAGCCUACCAUCCUUGAAGGC-N40-CAGUAUGUCCACCUUACC	Selection library
Rz1	GGACAUACUGAGCCUUCAACCAGCCUACCAUCCUGAAGGCAUGAUGCU-AACCACGCCGUCCCCUGAUGCUCGUACAUAGGCAGUAUGUCCACCUUACC	Clone 1 EB3
Rz2	GGACAUACUGAGCCUUCAACCAGCCUACCAUCCUGAAGGCAUGAUGCU-AACCACGCCGUCCCCUGAUGCUCGUACAUUGGCAGUAUGUCCACCUUACC	Clone 2 EB15
Rz2_ΔL1	GGUUGAAGGCAUGAUGCUAACCACGCCGUCCCCUGAUGCUCGUACAU-UGGCAGUAUGUCCACCUUACC	EB15_trans
Rz3	GGCCAGCCUACCAUCCUGAAGGCAUGAUGCUAACCAC-GCCGUCCCCUGAUGCUCGUACAUUGGCAGUAUGUCCACCUUACC	EB15 trans_5' con-necting loop
Rz4	GGAGACAUGAUGCUAACCACGCCGUCCCCUGAUGCUCGUACAU-UGGCGAUACC	EB15 bimolecular construct_mutated binding arms
Rz5	GGUAUCGAGUCUCC UUCG GGAGACAUGAUGCUAACCACGCCGUCCCCU	CSAR engineered *in trans*

Rz6	GGAAGGAGUCUCGUUCGCGAGACAUGAUGCUAACCACGCCGUCCCCU	CSAR engineered *in trans*_mutated binding arm

Supporting Information Table S5. Enrichment level and selection conditions. ON = overnight (i.e., 15-17 h); (-) denotes undetectable enrichment level.

***In vitro* selection**			
**round**	**eluted fraction, %**	**incubation time**	**[MgCl$_2$]**
1-2	-	ON	40 mM
3	0.047		
4	-		
5	5.028		
6	3.259		
7	4.467		
8	4.542	4 h	

Supporting Information Table S6. ESI-MS of synthetic RNAs and modified products.

No	Description	Chemical formula	Mass calculated, Da	Mass found, Da
R1	Parent RNA (23) engineered *in trans*	$C_{218}H_{271}N_{85}O_{160}P_{22}$	7319.99152	7320.07188
R1_Bn-prop	R1 modified by Rz5 with BG-prop	$C_{229}H_{281}N_{85}O_{161}P_{22}$	7478.06468	7478.12376
R1_Bn-Oprop	R1 modified by Rz5 with BG-Oprop	$C_{228}H_{279}N_{85}O_{161}P_{22}$	7464.04903	7464.15322
R1_Bn-Oprop_6FAMN$_3$	R1 modified by Rz5 with BG-Oprop and clicked with 6FAMN$_3$	$C_{252}H_{297}N_{89}O_{167}P_{22}$	7922.17167	7922.26939
R2	Parent RNA (22) 3'-NH$_2$	$C_{212}H_{272}N_{76}O_{159}P_{22}$	7106.97676	7107.09571
R2_Bn-prop	R2 modified by Rz6 with BG-prop	$C_{223}H_{282}N_{76}O_{160}P_{22}$	7265.04993	7265.16261
R2_Bn	R2 modified by Rz6 with Bn^{6}G	$C_{219}H_{278}N_{76}O_{159}P_{22}$	7197.02371	7197.11417
R2_Bn-Oprop	R2 modified by Rz6 with BG-Oprop	$C_{222}H_{280}N_{76}O_{160}P_{22}$	7251.03373	7250.98269
R2_Bn-Oprop_Cy5N$_3$	R2 modified by Rz6 with BG-Oprop and clicked with Cy5N$_3$	$C_{257}H_{324}N_{82}O_{167}P_{22}S_2$	7975.30502	7975.24063

11 Supporting Information Figures

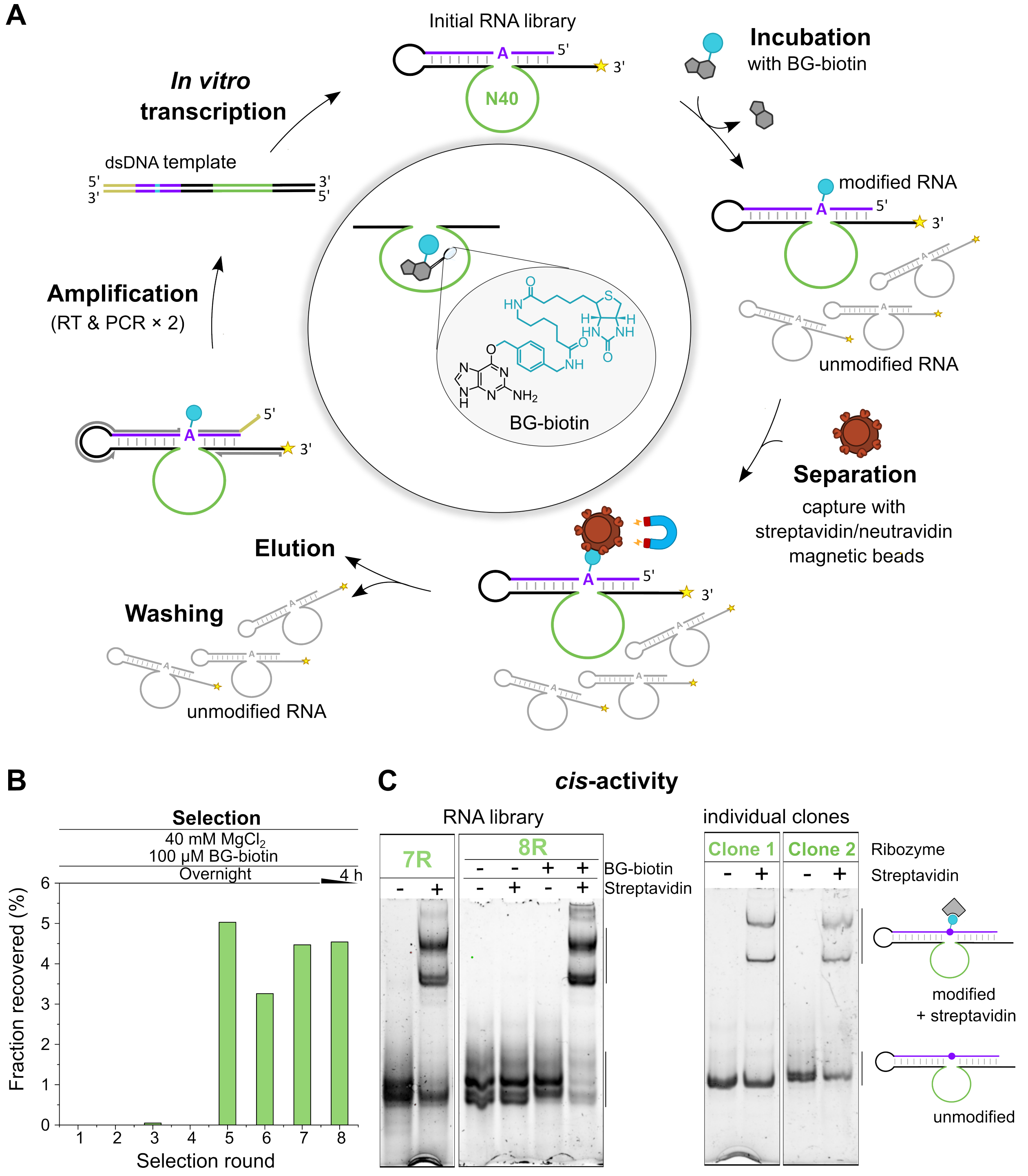

Figure S1. *In vitro* selection of alkyltransferase ribozymes. **A** *In vitro* selection scheme. The initial RNA library contained a N40 random region flanked by binding arms which are hybridized to a target substrate sequence bearing unpaired adenosine (A). After incubation with the cofactor BG-biotin, the active species are separated by pulling them down with streptavidin/neutravidin coated magnetic beads followed by their elution, amplification and *in vitro* transcription. **B** Overview of the selection progress including enrichment level at each round and selection conditions. The percentage of fraction recovered in each round was determined based on fluorescence spectra of initial and eluted 3'-lucifer yellow labeled RNA libraries. **C** *Cis*-activity of the enriched RNA library of round 7 and round 8 and individual clones using streptavidin gel-based assay. RNAs of the respective round were incubated with BG-biotin (cyan) under selection conditions followed by the coupling with streptavidin (gray). Purple sphere schematically represents the expected modification site.

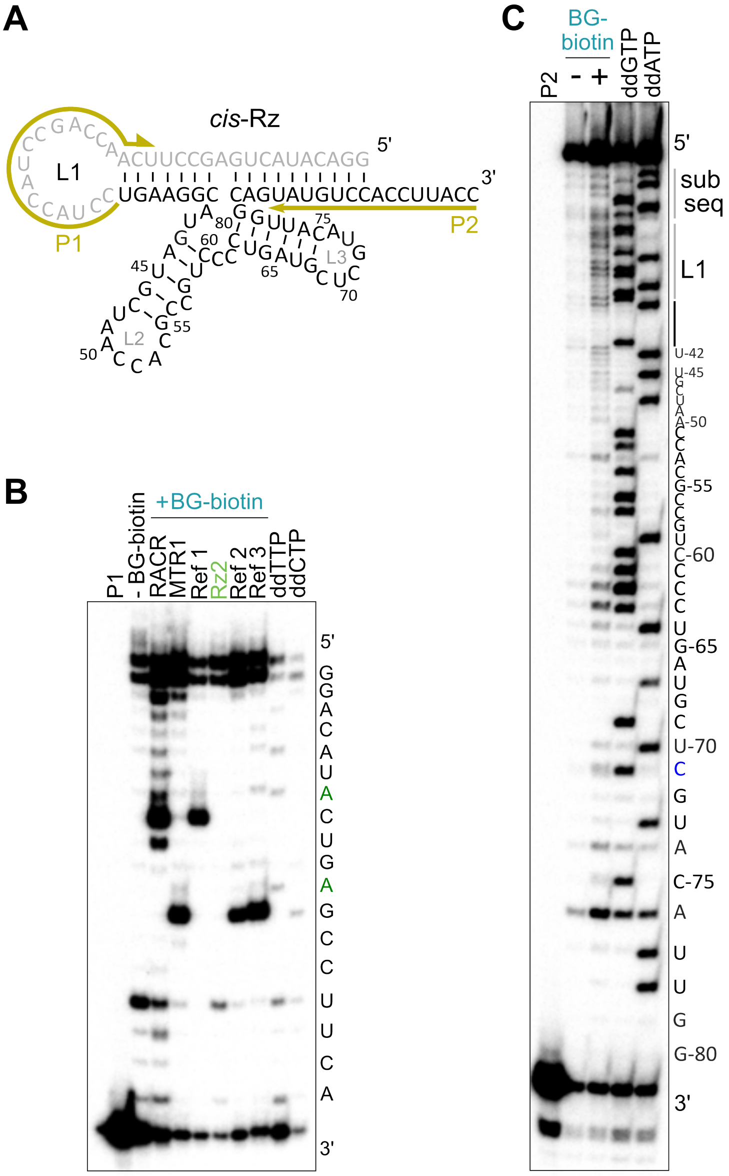

Figure S2. Determination of the modification site in alkylation by the evolved ribozyme. **A** Predicted secondary structure[13] of the selected ribozyme with schematically represented primers P1 and P2 used for RT primer extension assay. **B, C** RT primer extension assay on *cis*-active ribozyme (Rz2) using primers P1 and P2 annealed as depicted in **A**. Reactions were incubated overnight (0.4 µM RNA, 100 IPS 5'-^{32}P-labeled P1 or P2, 100 µM BG-biotin, 40 mM $MgCl_2$, 50 mM HEPES, pH 7.5, 37 °C) with the selection cofactor BG-biotin. No activity was detected by RT primer extension experiments compared to those using MTR1, RACR and additional selected alkyltransferase ribozymes modifying substrate RNAs (Ref 1, Ref 2, Ref 3). In case of MTR1, RACR and Ref 1 – 3, prominent stopping bands indicate the modification sites at a defined nucleotide highlighted in green (shown as references).

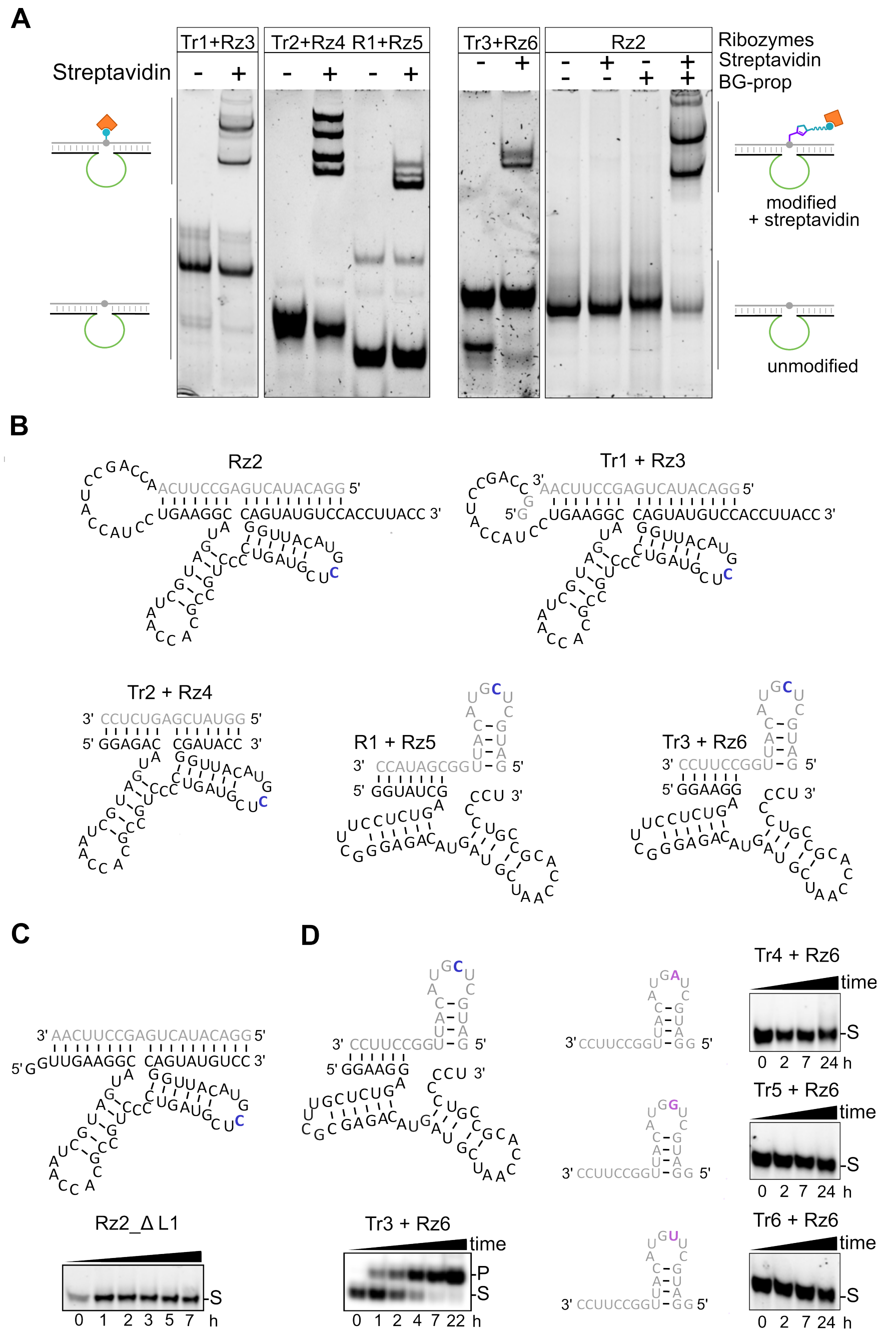

Figure S3. *Cis* and *trans*-activity of ribozymes. **A** Streptavidin gel-shift assay of *cis*- and engineered bimolecular ribozyme constructs. RNAs were first incubated with 100 µM BG-prop (violet) and 40 mM $MgCl_2$ at pH 6.0 followed by the coupling with biotin-PEG_3-azide (cyan) or directly incubated with 100 µM BG-biotin (cyan). After incubation, modified RNAs were captured with streptavidin (orange). **B** Predicted secondary structures of the ribozyme constructs tested in **A**. **C** Activity assay of the bimolecular construct generated by removing the connecting loop L1. The ribozyme was annealed to the substrate RNA (Tr1) and reacted with BG-prop (**2**) under single turnover conditions (1 µM substrate RNA, 10 µM CSAR, 100 µM BG-prop (**2**), pH 6.0, 40 mM $MgCl_2$, 37 °C, 7 h). No product formation was detected when resolving the substrate RNA (*trans*-setup). D Testing mutation of C7 in kinetic assays for *trans* constructs under single turnover conditions (1 µM substrate RNA, 10 µM CSAR, 100 µM BG-prop (**2**), pH 6.0, 40 mM $MgCl_2$, 37 °C, 22 – 24 h). Modification site C7 is marked in blue, corresponding mutated nucleotides at C7 are depicted in pink. Representative PAGE images for n=2 independent experiments are shown.

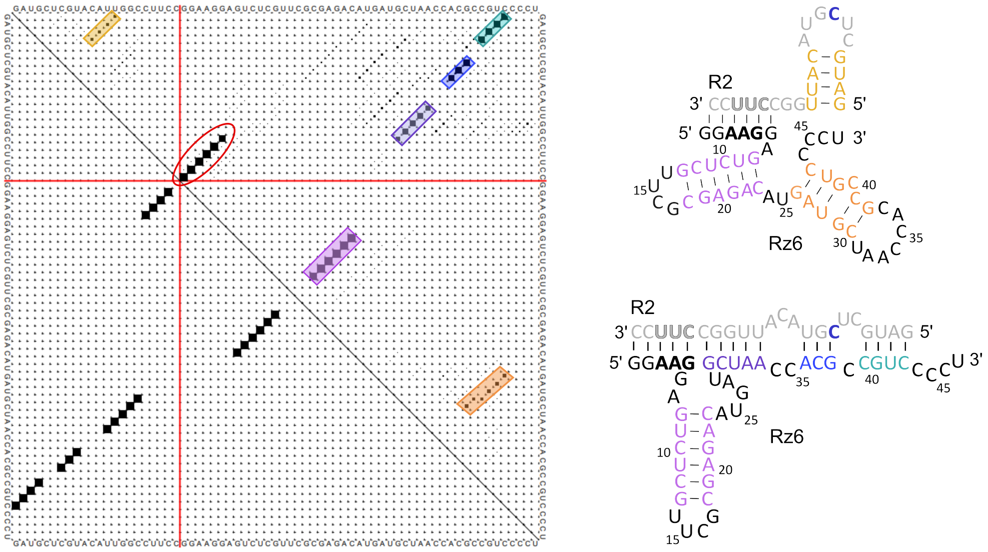

Figure S4. Predicted secondary structure of CSAR. RNA dot-plot of the *trans*-active ribozyme construct R2 + Rz6 predicted by Vienna RNAfold.[13] Formation of the new binding arm (BA) between the RNA substrate and the ribozyme is shown in red circle. Two main alternative structures of the complex R2 + Rz6 extracted from the dot plot are shown on the right. Watson-Crick base-paired stems are color coded.

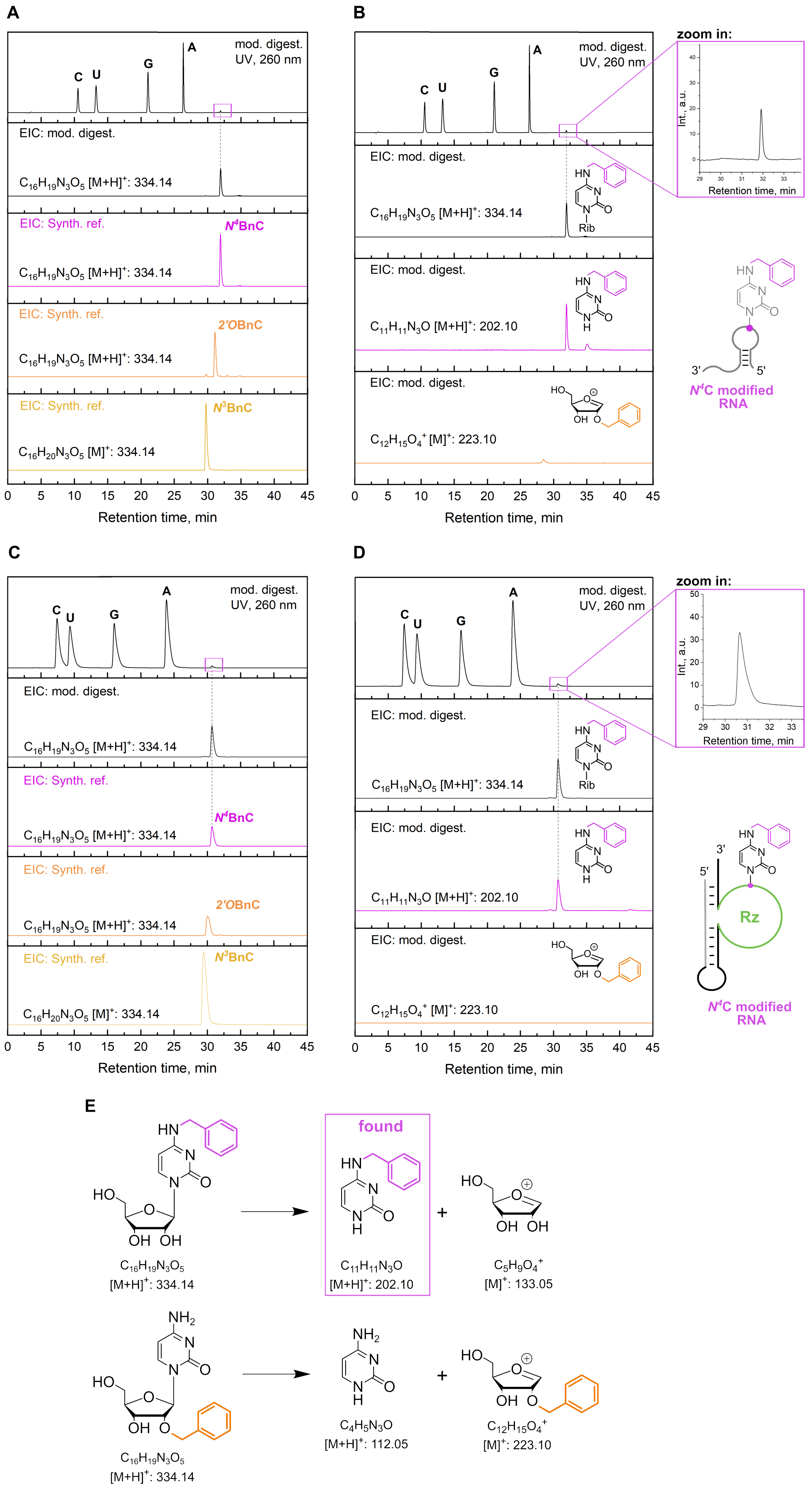
A
B
C
D
E
mod. digest.
UV, 260 nm
C
U
G
A
EIC: mod. digest.
C16H19N3O5 [M+H]+: 334.14
EIC: Synth. ref.
N4BnC
2'OBnC
N3BnC
C16H20N3O5 [M]+: 334.14
C11H11N3O [M+H]+: 202.10
C12H15O4+ [M]+: 223.10
Rib
zoom in:
Int., a.u.
Retention time, min
N4C modified
RNA
Rz
3'
5'
found
C16H19N3O5
[M+H]+: 334.14
C11H11N3O
[M+H]+: 202.10
C5H9O4+
[M]+: 133.05
C4H5N3O
[M+H]+: 112.05
C12H15O4+
[M]+: 223.10

Figure S5. LC-MS analysis of digested benzylated product obtained in alkylation by *trans*- and *cis*-active CSAR. **A, C** Extracted ion chromatograms (EIC (*m/z* (M+H$^+$) = 334.14 ± 0.02)) of the modified RNA (black) using either *trans*-active or *cis*-active ribozymes and extracted ion chromatograms of synthetic references N^4BnC, 2'*O*-BnC and N^3BnC (magenta, orange and yellow respectively) indicate the N^4BnC as the modification site. **B, D** Extracted ion chromatograms (EIC (*m/z* (M+H$^+$) = 334.14 ± 0.02)) of the modified RNA (black) using either *trans*-active or *cis*-active CSAR and extracted ion chromatograms (EIC (*m/z* (M+H$^+$) = 202.10 ± 0.02) and EIC (*m/z* (M$^+$) = 223.10 ± 0.02)) calculated for the fragmented ions formed as a result of fragmentation of either N^4-BnC or *2'O*-BnC. The new peak emerged in EIC (*m/z* (M+H$^+$) = 202.10 ± 0.02)) calculated for the modified base supports N^4BnC as the modification site. Digestion of the benzylated product for LC-MS was performed in (n=2) independent experiments for both *trans*- and *cis*-ribozymes. **E** Reaction schemes of fragmentation for N^4BnC and 2'*O*-BnC.

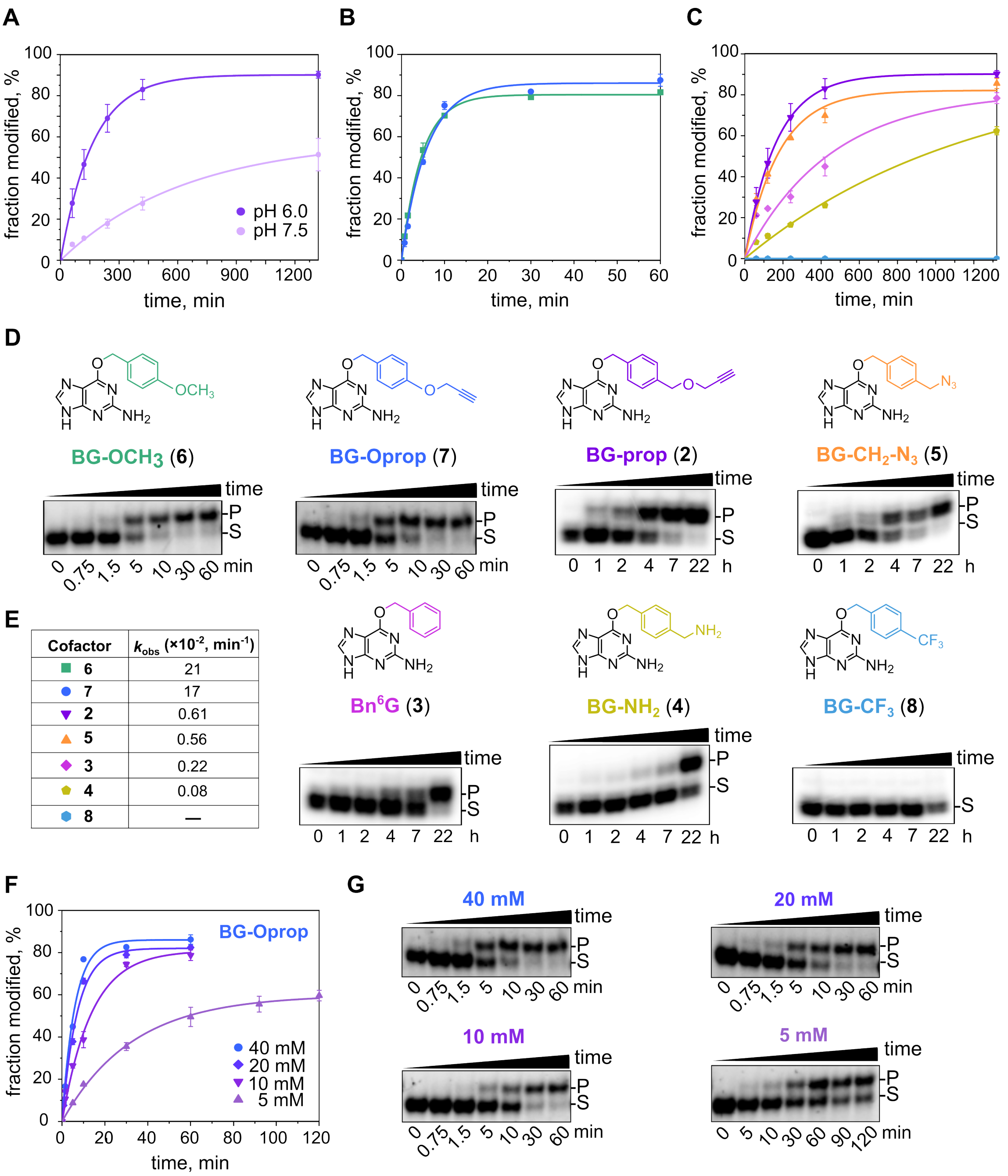

Cofactor	k_{obs} (×10^{-2}, min^{-1})
6	21
7	17
2	0.61
5	0.56
3	0.22
4	0.08
8	—

Figure S6. Kinetic characterization and cofactor scope of CSAR. **A** Comparison of alkylation reaction rate of a 22-mer RNA substrate (Tr3) by the ribozyme (Rz6) at pH 6.0 over pH 7.5 under single turnover conditions (100 µM **2**, 40 mM $MgCl_2$, 37 °C for 22 h). Data represent mean values of (n=3) independent experiments. **B, C** Comparison of fraction modified (%) for the alkylation reaction by *trans*- Rz6 in the complex with Tr3 using various O^6-modified guanine cofactors under single turnover conditions (1 µM Tr3, 10 µM Rz6, 100 µM cofactor, pH 6.0, 40 mM $MgCl_2$, 37 °C, 1 h (for fast reactions) or 22 h (for slow reactions)). Data represent mean values of (n=3) independent experiments for BG-prop and (n=2) independent experiments for all other cofactors. **D, G** Representative PAGE images of kinetic assays obtained from alkylation reactions of a 22-mer RNA substrate (Tr3) by CSAR using different O^6-modified guanines (D) and various $MgCl_2$ concentrations (G) under single turnover conditions (1 µM Tr3, 10 µM Rz6, 100 µM cofactor (D) / **7** (G), pH 6.0, 37 °C, 1 h (for fast reactions) or 22 h (for slow reactions) in D / 1 h (for reactions with 40-10 mM $MgCl_2$) or 2 h (for the reaction with 5 mM $MgCl_2$)) in G. $MgCl_2$ at 40 mM (D) or at different concentration (G) was used. Data represent mean values of (n=3) independent experiments for G and for cofactor **7** in D and those of (n=2) for all other cofactors in D. **E** Overview of k_{obs} values obtained from **B** and **C**. **F** Comparison of fraction modified (%) for alkylation reactions by *trans*-CSAR in the complex with Tr3 using different concentrations of $MgCl_2$: 40 mM, 20 mM, 10 mM, 5 mM under single turnover conditions (1 µM Tr3, 10 µM Rz6, 100 µM cofactor, pH 6.0, 37 °C, 1 h (for reactions with 40 mM, 20 mM, 10 mM of $MgCl_2$) or 2 h (for the reaction with 5 mM $MgCl_2$). Data represent mean values of (n=3) independent experiments for all $MgCl_2$ concentrations.

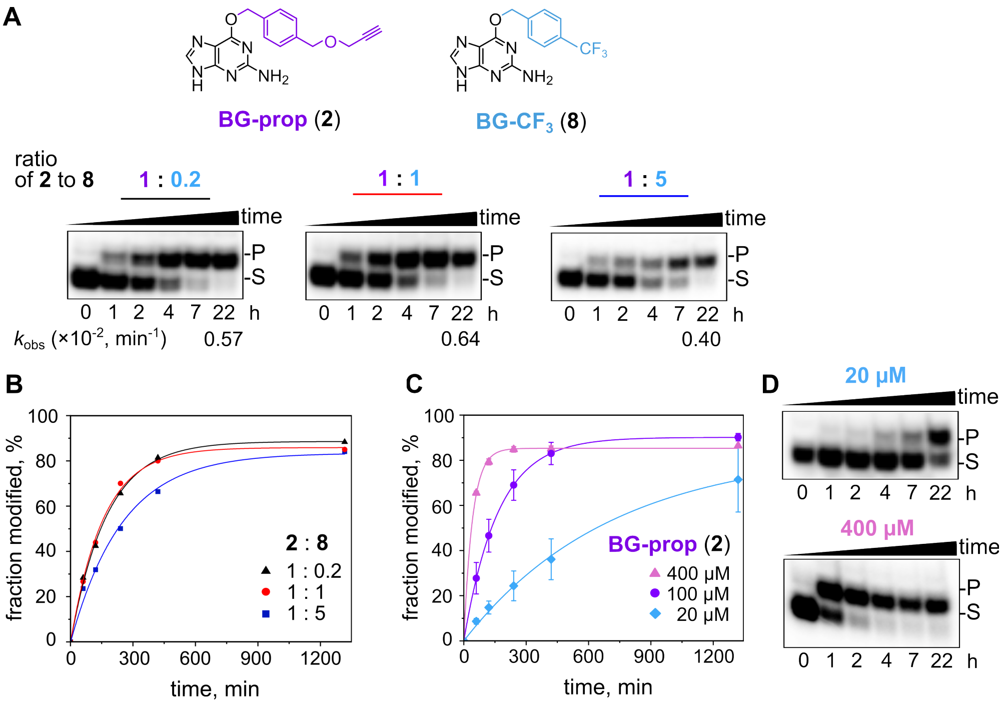

Figure S7. Concentration-dependence and competition of cofactor 2 and 8. **A** PAGE images of the alkylation reaction by CSAR (Rz6) using Tr3 and the cofactor BG-prop (**2**) in the presence of BG-CF_3 (**8**) under single turnover conditions (1 µM RNA, 10 µM CSAR, 100 µM **2**, 20 µM / 100 µM/ 500 µM **8**, pH 6.0, 40 mM $MgCl_2$, 37 °C, 22 h). **B** Fraction modified (%) over time from the conditions in A. **C** Fraction modified (%) over time for the alkylation reaction by CSAR (Rz6) using 22-mer substrate RNA (Tr3) and the cofactor **2** at different concentrations under single turnover conditions (1 µM RNA, 10 µM CSAR, 20/100/400 µM (**2**), pH 6.0, 40 mM $MgCl_2$, 37 °C, 22 h). **D**. Representative PAGE images for data shown in C. The PAGE image for the reaction at 100 µM of **2** is shown in Figure S6D.

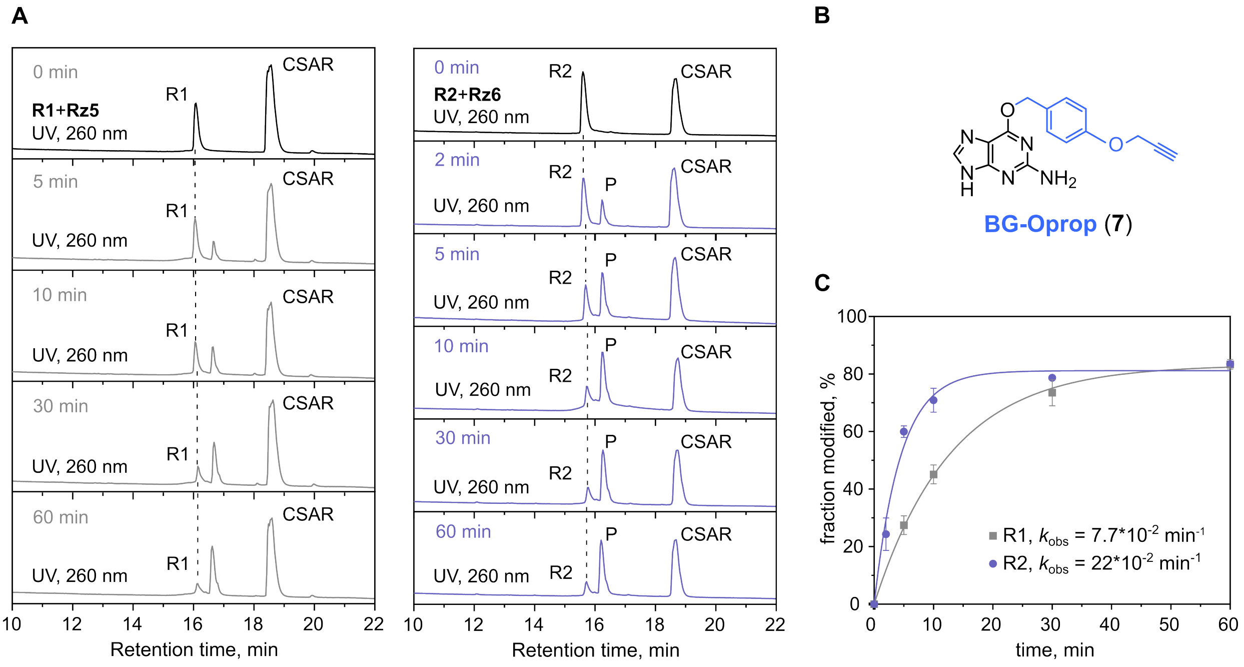

Figure S8. Comparison of the reaction rate of CSAR-catalyzed RNA alkylation using substrate RNAs with different binding arms. **A** Kinetic assay of the alkylation reaction by CSAR (Rz5 and Rz6) using R1 (left, gray) and R2 (right, lilac), respectively, analyzed on anion-exchange HPLC at 260 nm (10 µM RNA, 11 µM CSAR, 100 µM (**7**), pH 6.0, 40 mM $MgCl_2$, 37 °C). **B** Chemical formula of the cofactor used in A. **C** Comparison of fraction modified (%) for the alkylation reaction shown in A. Data represent mean values of (n=2) independent experiments.

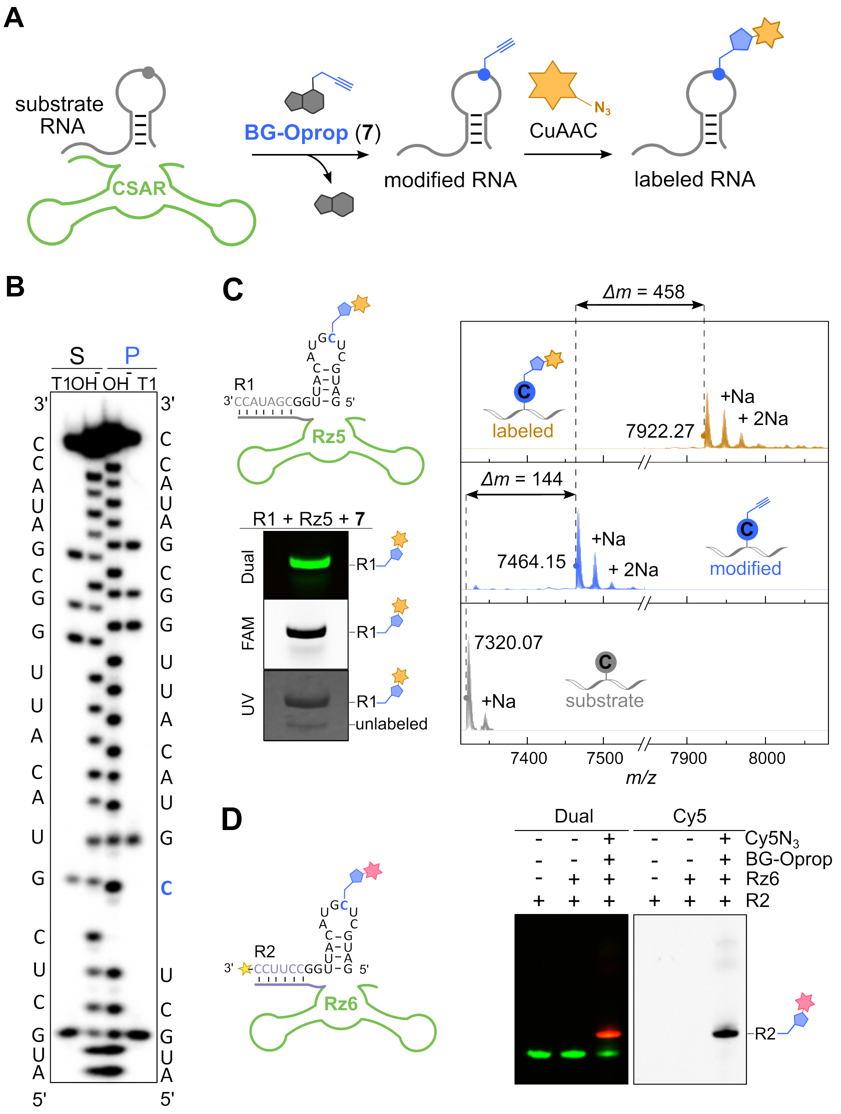

Figure S9. Installation of clickable moiety using BG-Oprop by CSAR for Cu-catalyzed click reaction. **A** Schematic representation of the RNA modification and labeling mediated by CSAR. The modification site, cytidine, is shown in a blue sphere and a fluorophore is depicted with an ocher star. **B** Representative gel image for RNase T1 digestion and alkaline hydrolysis of 5'-^{32}P-labeled reaction product (P) generated by the engineered *trans*-ribozyme using BG-Oprop (**7**) and those of unmodified substrate RNA (S) shown in comparison. The identified modification site, cytidine, is marked bold blue. **C** Schematic representation of the 23-mer substrate RNA (R1) annealed to the corresponding CSAR variant (Rz5) and modified with **7** (100 µM **7**, 40 mM $MgCl_2$, 16 h, 37 °C) and subsequently labeled with the 6-FAM-N_3 via CuAAC. Variable substrate binding arm is depicted in gray (R1) Representative PAGE images of the labeling reaction indicating the labeled product and deconvoluted HR-ESI-MS of the 23-mer substrate RNA (R1, gray), isolated modified product (blue) and product labeled with 6-FAM-N_3 (ocher) are shown. **D**. Schematic representation of the 22-mer substrate RNA (R2) annealed to the corresponding CSAR variant (Rz6) and modified with **7** (100 µM **7**, 40 mM $MgCl_2$, 1 h, 37 °C) and further labeled with the Cy5N_3 via CuAAC (pink star). Variable substrate binding arm is depicted in lilac (R2). Representative PAGE images (overlay of Cy5 (red) and FAM (green) channels) of the labeling reaction indicating the labeled product (in red) are shown.

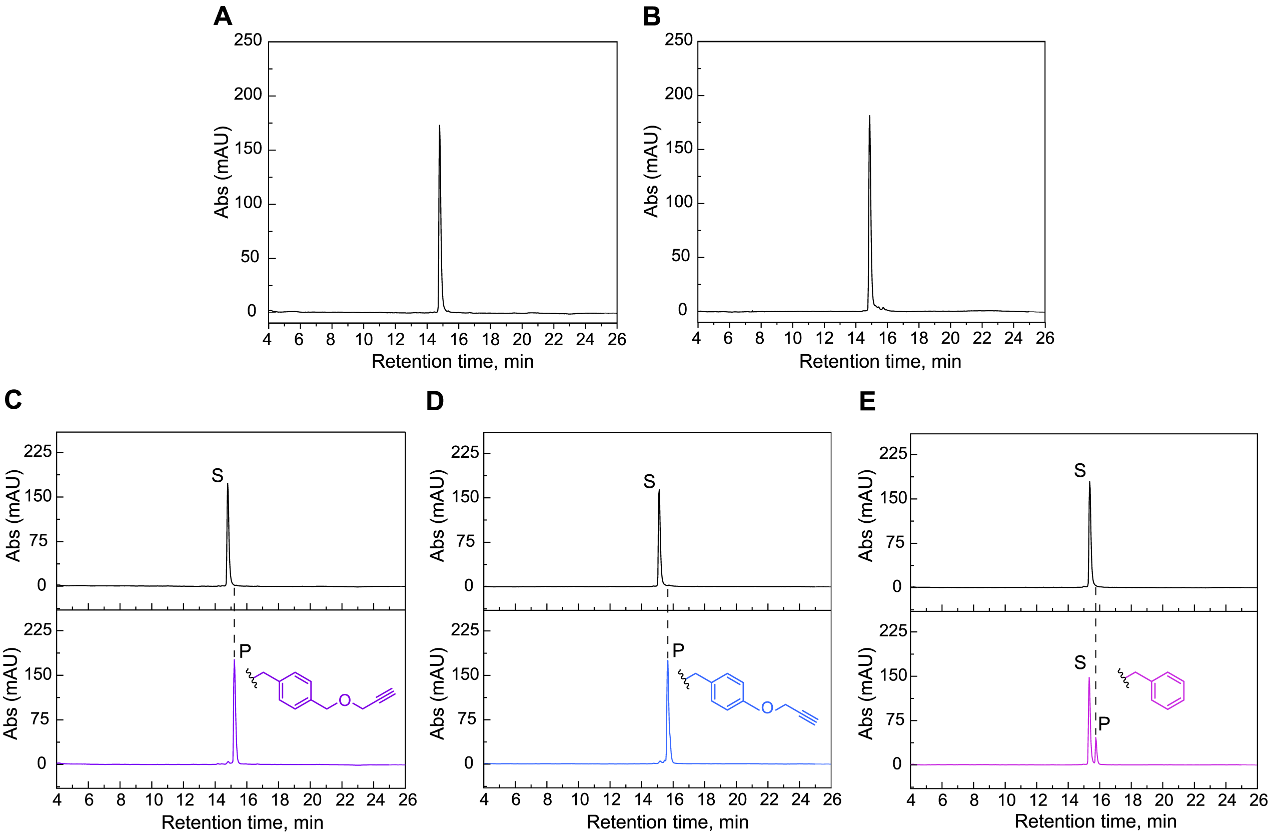

Figure S10. Anion-Exchange HPLC analysis of purified RNAs prepared by solid phase synthesis and isolated RNA products prepared by CSAR-catalyzed alkylation reaction. **A – B** UV traces (260 nm) of unmodified substrate RNAs: R1 (**A**), R2 (**B**). **C – E.** UV traces (260 nm) of unmodified substrate RNA (R1) and isolated products modified with CSAR (Rz5) using BG-prop (**C**), BG-Oprop (**D**), and Bn^{6}G (**E**) as cofactors. For R1 modified with Bn^{6}G (**E**), reaction was incomplete and modified and unmodified RNAs were not separable by 15% PAGE, hence a mixture of modified and unmodified RNAs was isolated and injected into an anion-exchange HPLC.

12 NMR spectra

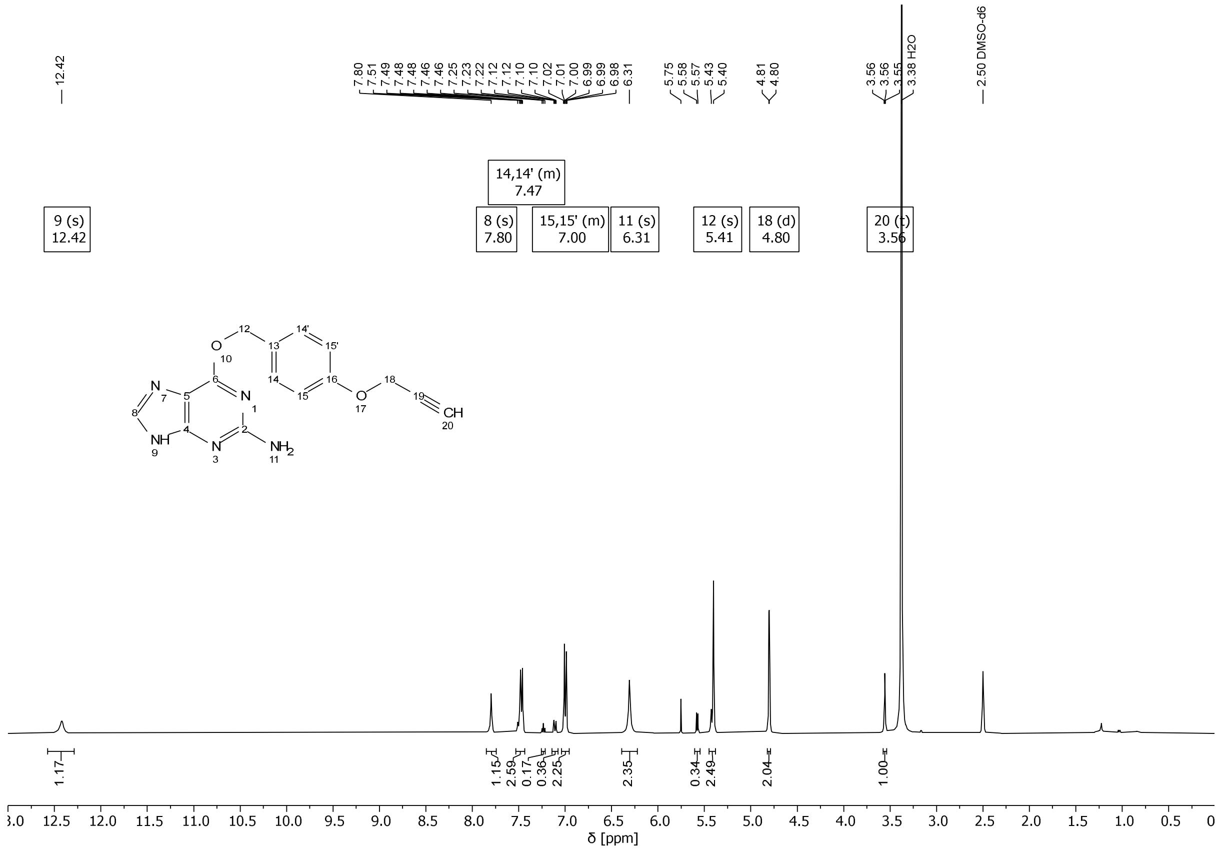

^{1}H NMR (400 MHz, DMSO-*d6*) of compound BG-Oprop (**7**) (14% of isomerized BG-Oallene (**7b**) was observed based on the NMR).

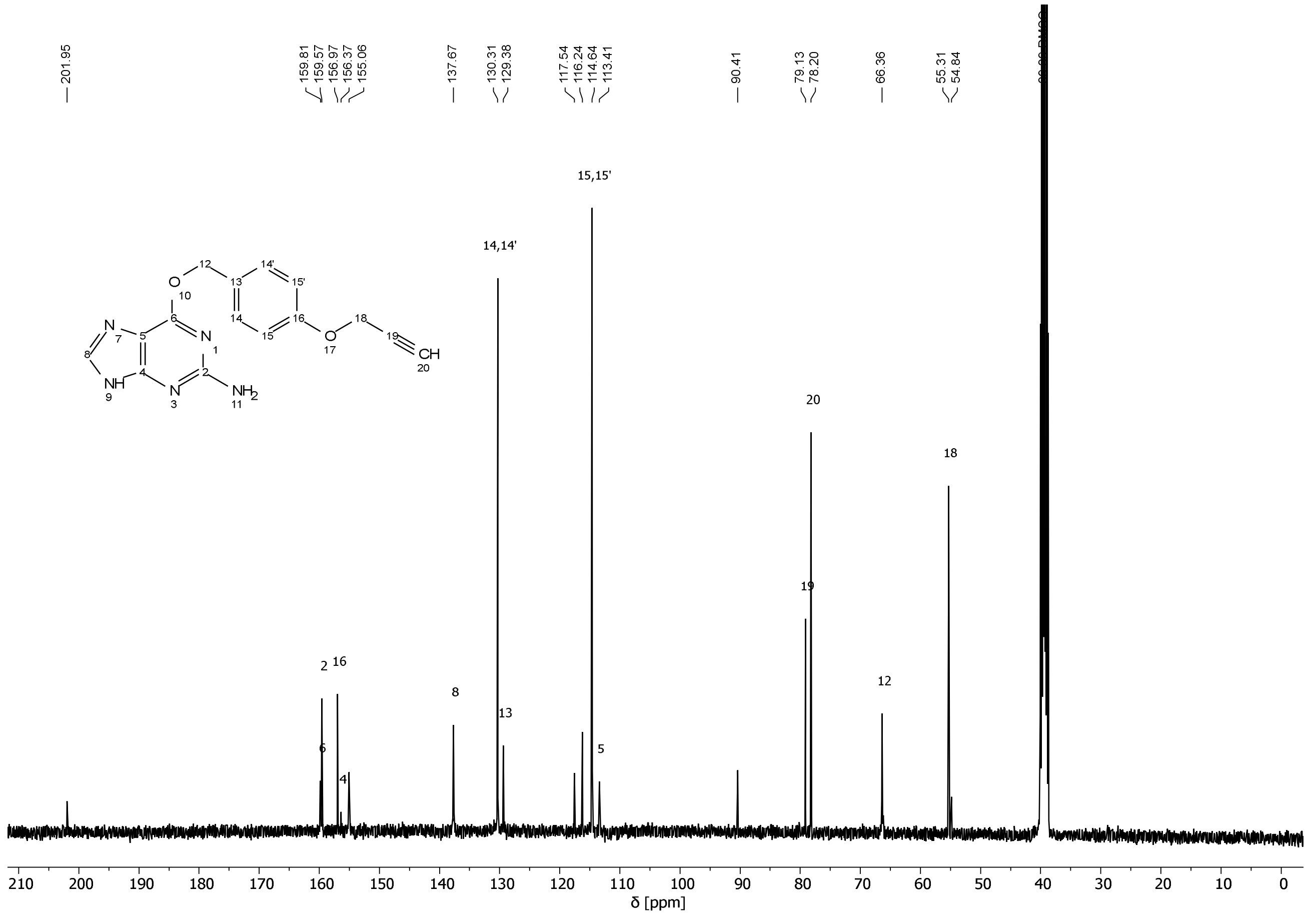

^{13}C NMR (101 MHz, DMSO-*d6*) of compound BG-Oprop (**7**). The NMR signal at 201.95 ppm is characteristic for quaternary carbon C19 in BG-Oallene (**7b**).

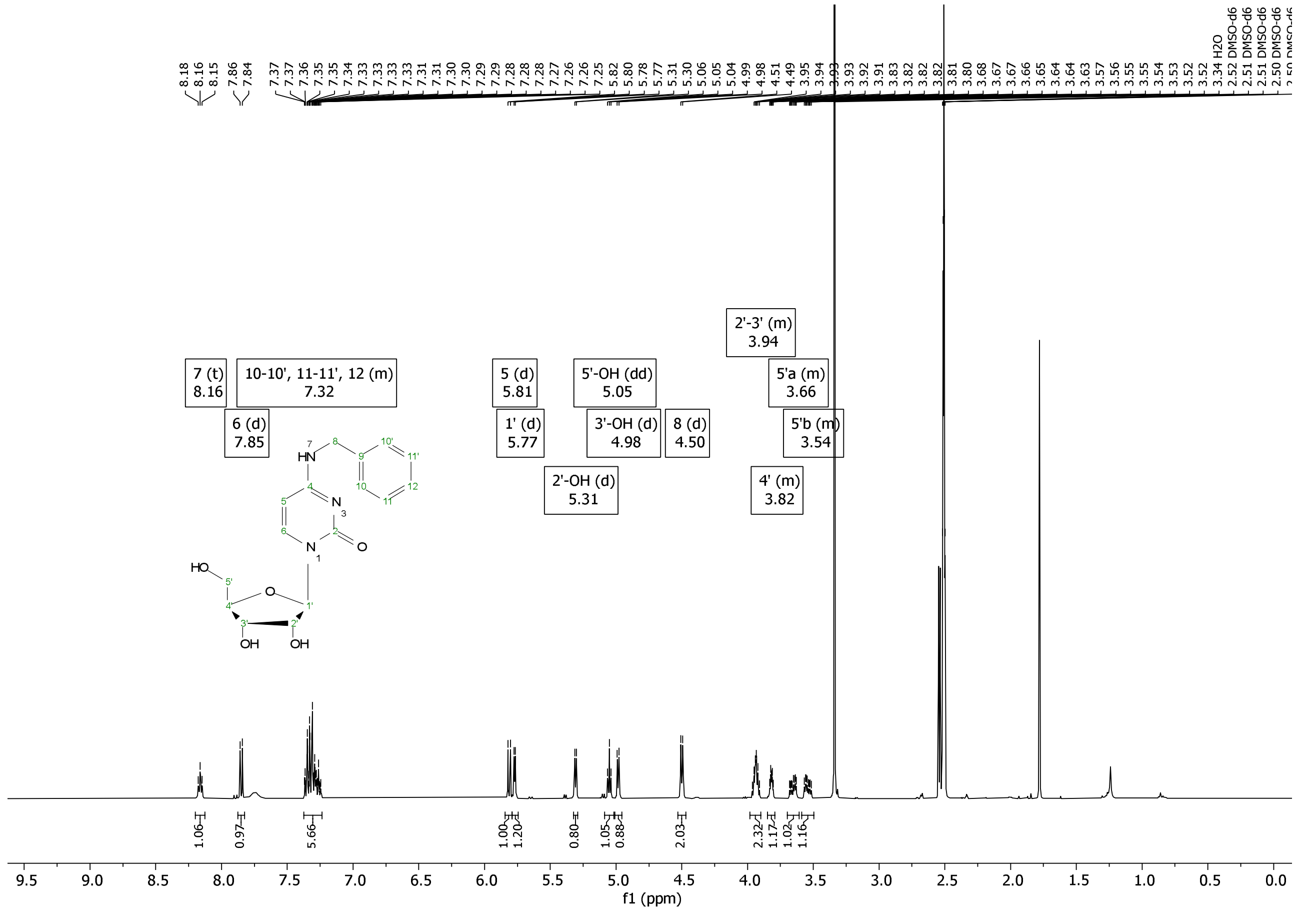

^{1}H NMR (400 MHz, DMSO-*d6*) of compound ***N*^{4}BnC**. The NMR signals at 7.75 ppm (s), 2.54 ppm (d) and 1.78 ppm (s) correspond to NH- proton and protons of methyl and acetyl groups from *N*-methylacetamide respectively that was formed during deprotection of **7**.

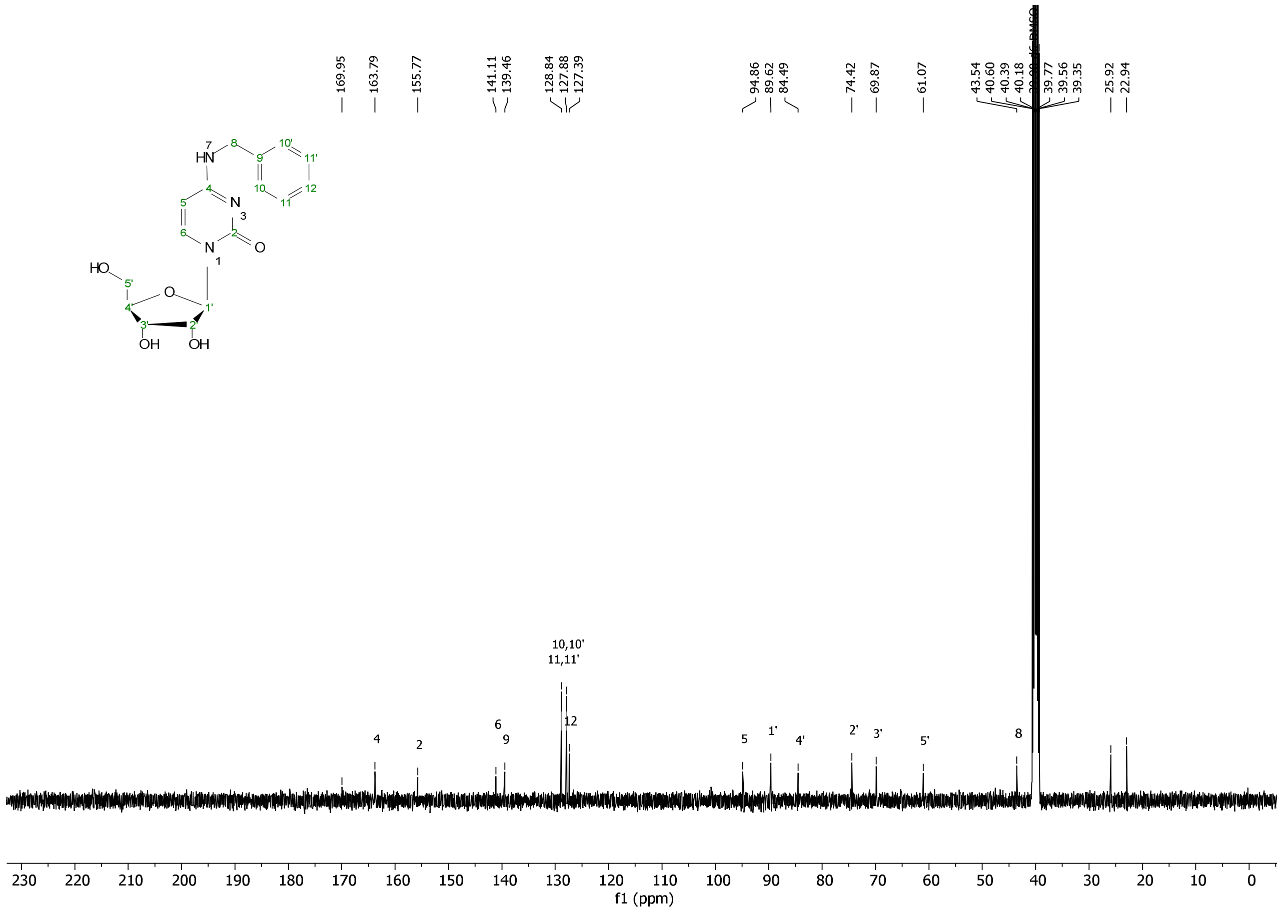

^{13}C {^{1}H} NMR (101 MHz, DMSO-*d6*) of compound ***N*^4^BnC**. The NMR signals at 169.95 ppm, 25.92 ppm and 22.94 ppm correspond to carbons of carbonyl, (<u>C</u>H_3-NH-) and (<u>C</u>H_3-C(O)-) groups from *N*-methylacetamide that was formed during deprotection of **7**.

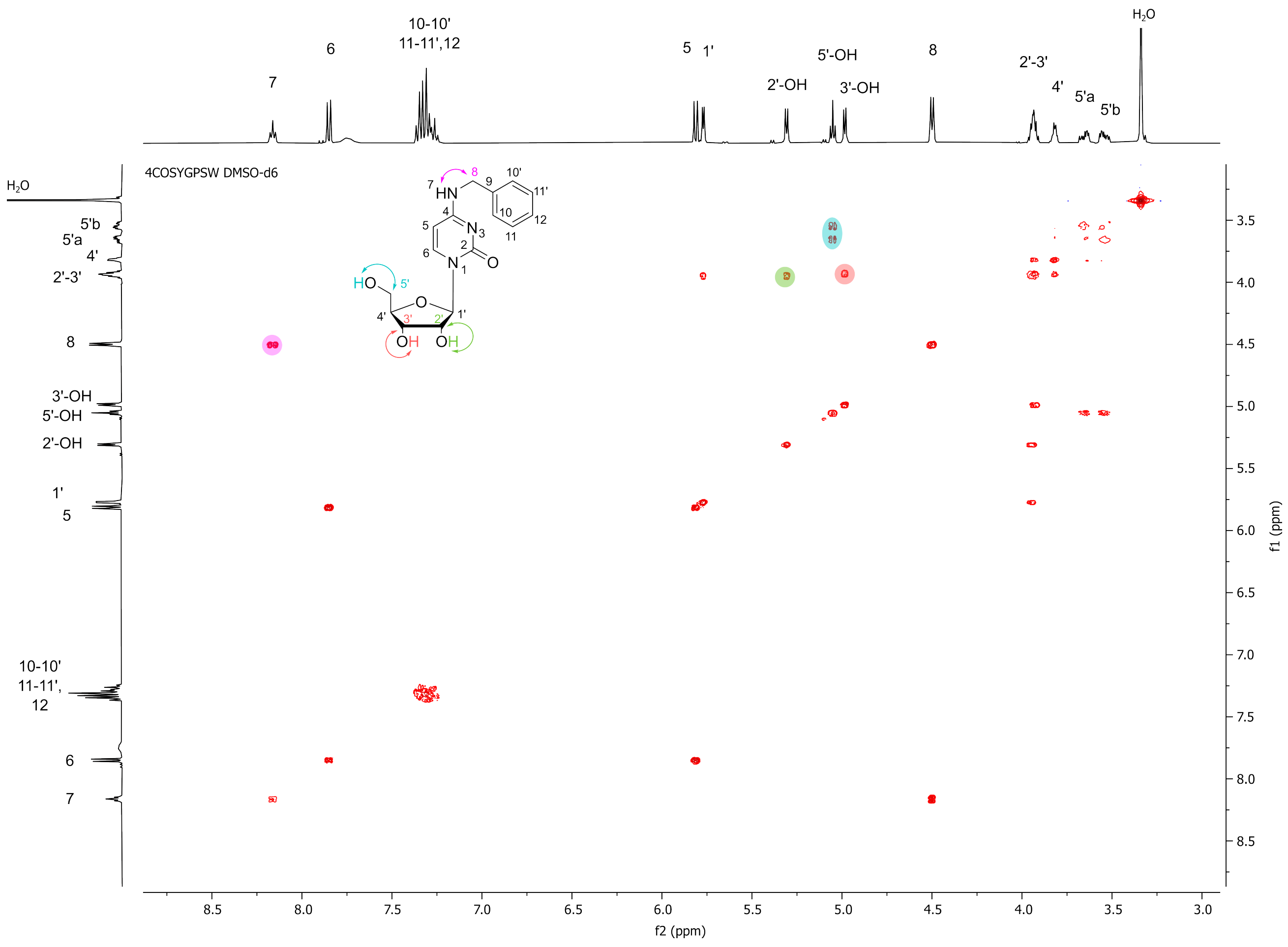

^{1}H-^{1}H COSY NMR (400 MHz, DMSO-*d6*) of compound ***N*^{4}BnC** (zoomed 3.0 – 9.0 ppm).

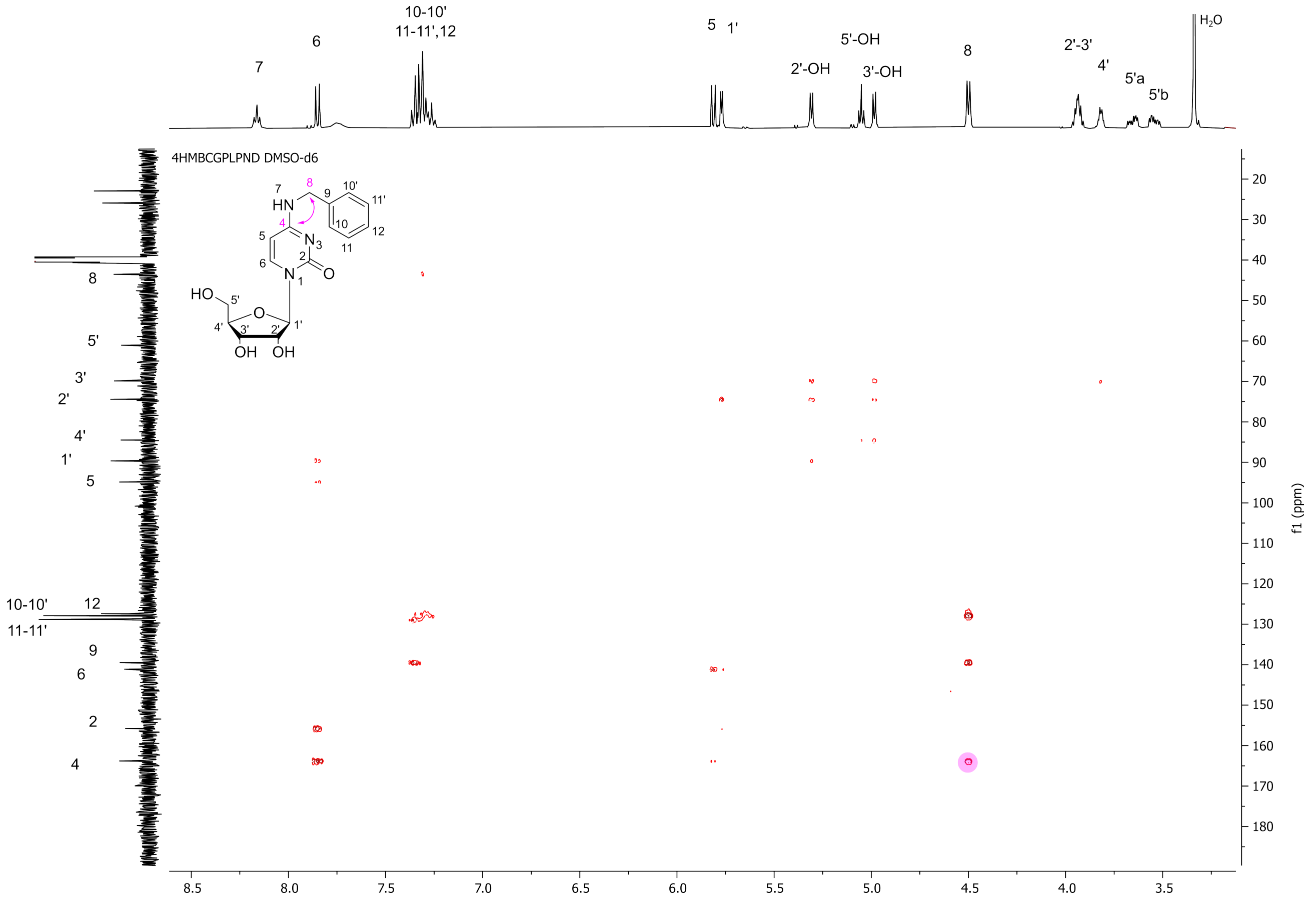

^{1}H-^{13}C HMBC NMR (400 MHz, DMSO-*d6*) of compound ***N*^{4}BnC** (zoomed 3.0 – 8.5; 15 – 185 ppm).

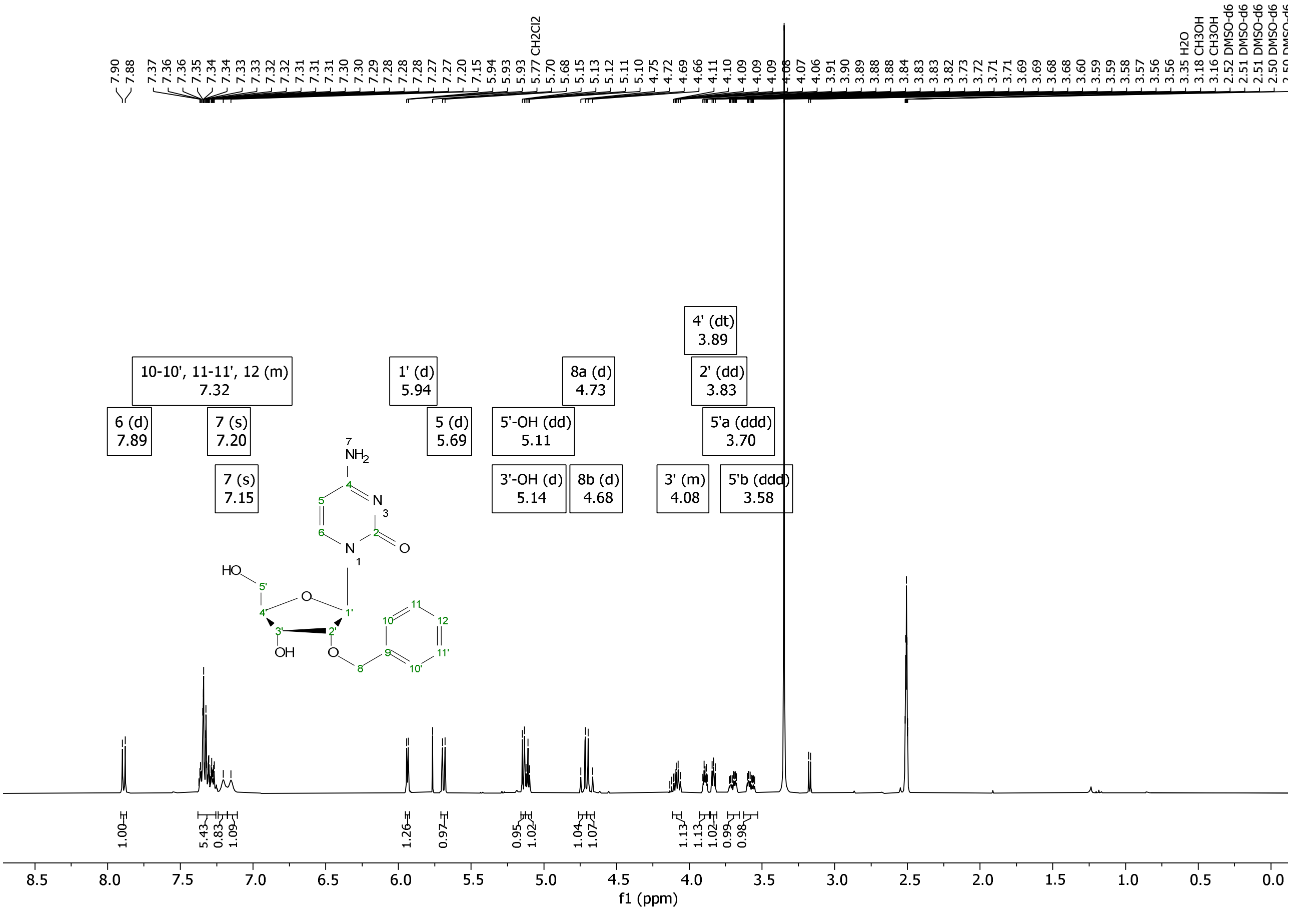

^{1}H NMR (400 MHz, DMSO-*d6*) of compound **2'*O*BnC**. The NMR signals at 5.77 ppm (s) and 3.17 ppm (d) correspond to residual dichloromethane and methanol respectively used for purification of **2'*O*BnC**. The signal of OH group from methanol is overlapped with the signal of H-3' **2'*O*BnC** (4.08 ppm (m)).

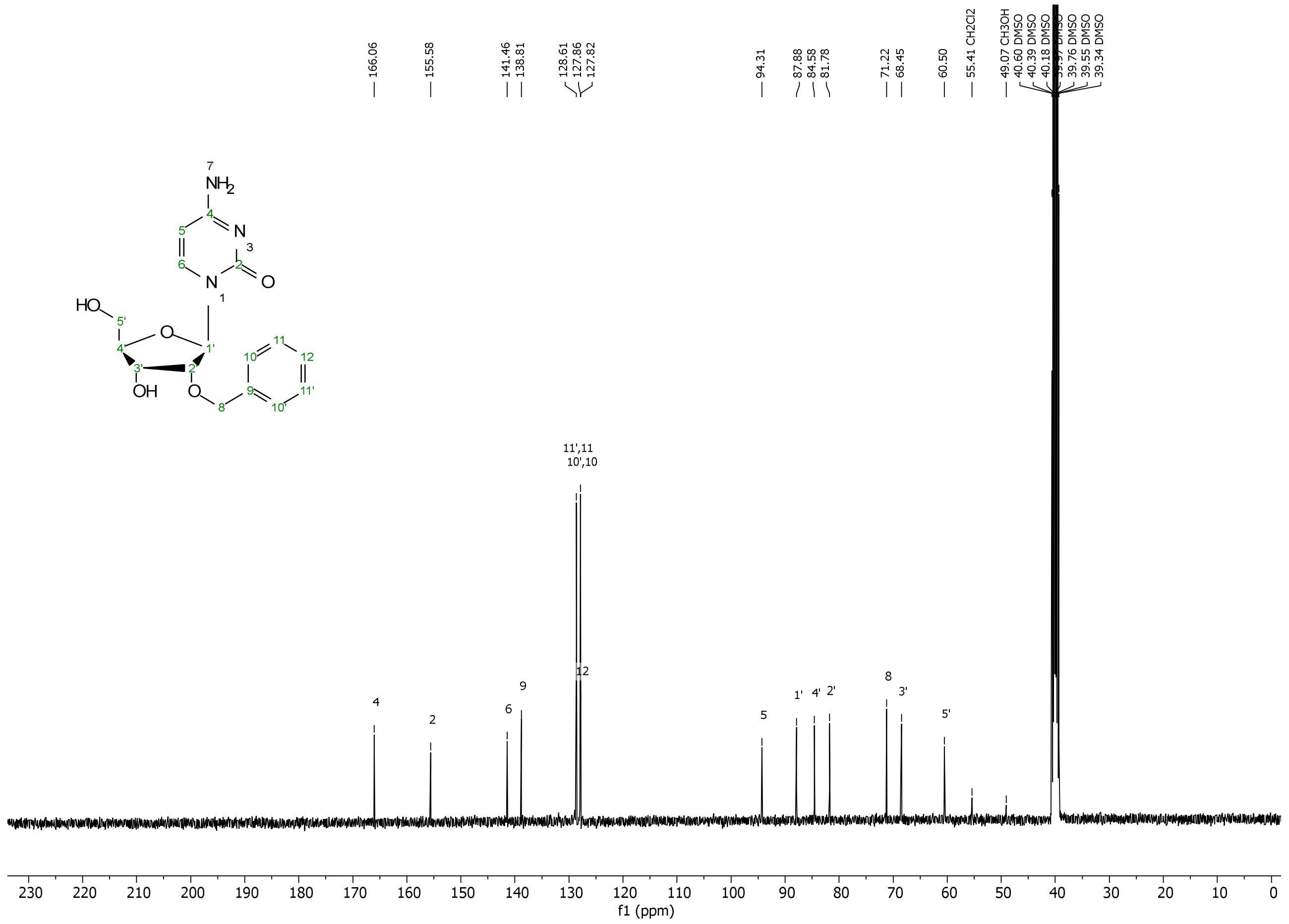

^{13}C {^{1}H} NMR (101 MHz, DMSO-*d6*) of compound **2'*O*BnC**. The NMR signals at 55.41 ppm and 49.07 ppm and correspond to residual dichloromethane and methanol respectively that were used for purification of **2'*O*BnC**.

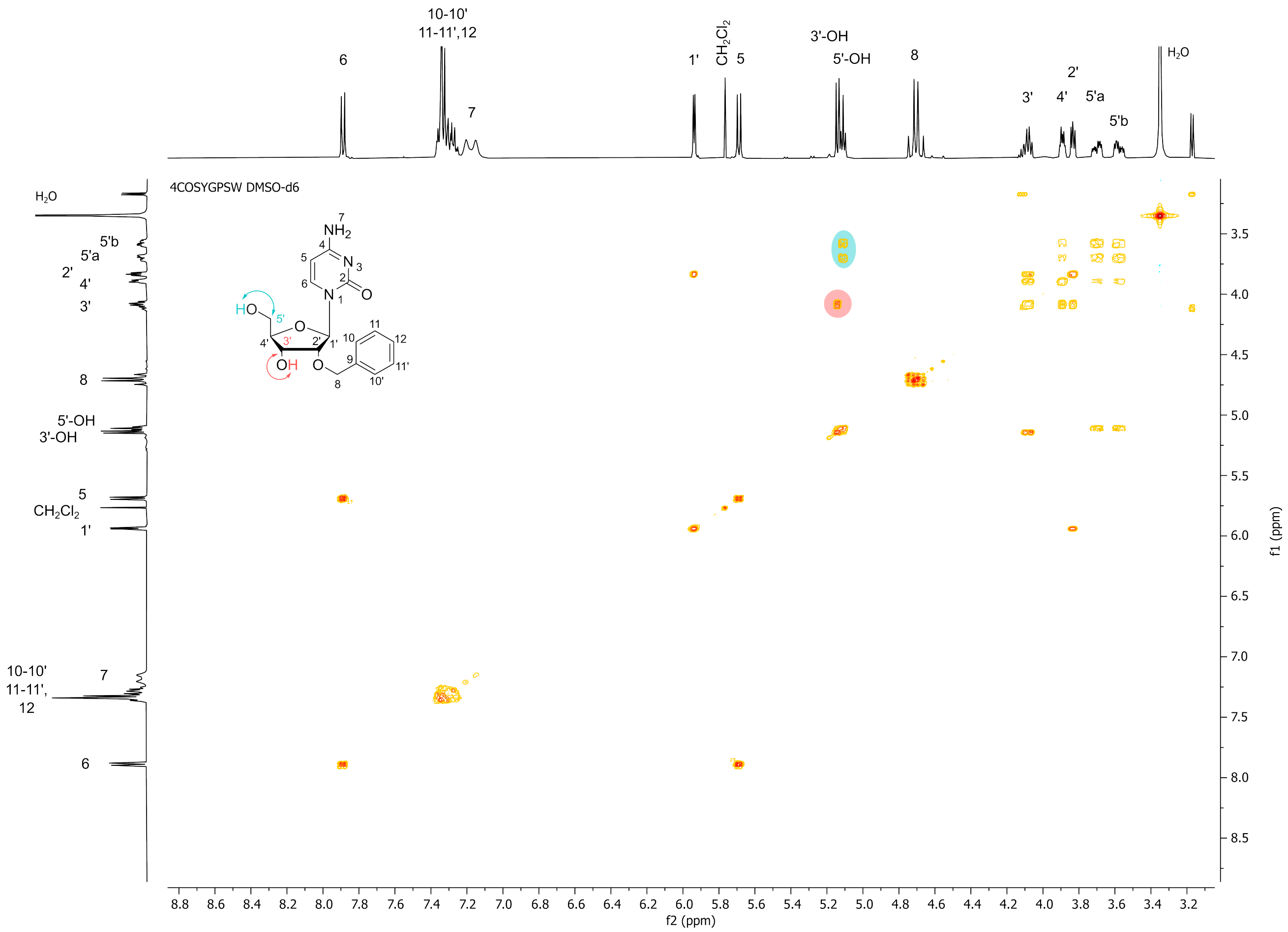

^{1}H-^{1}H COSY NMR (400 MHz, DMSO-*d6*) of compound **2'*O*BnC** (zoomed 3.2 – 8.8; 3.0 – 9.0 ppm).

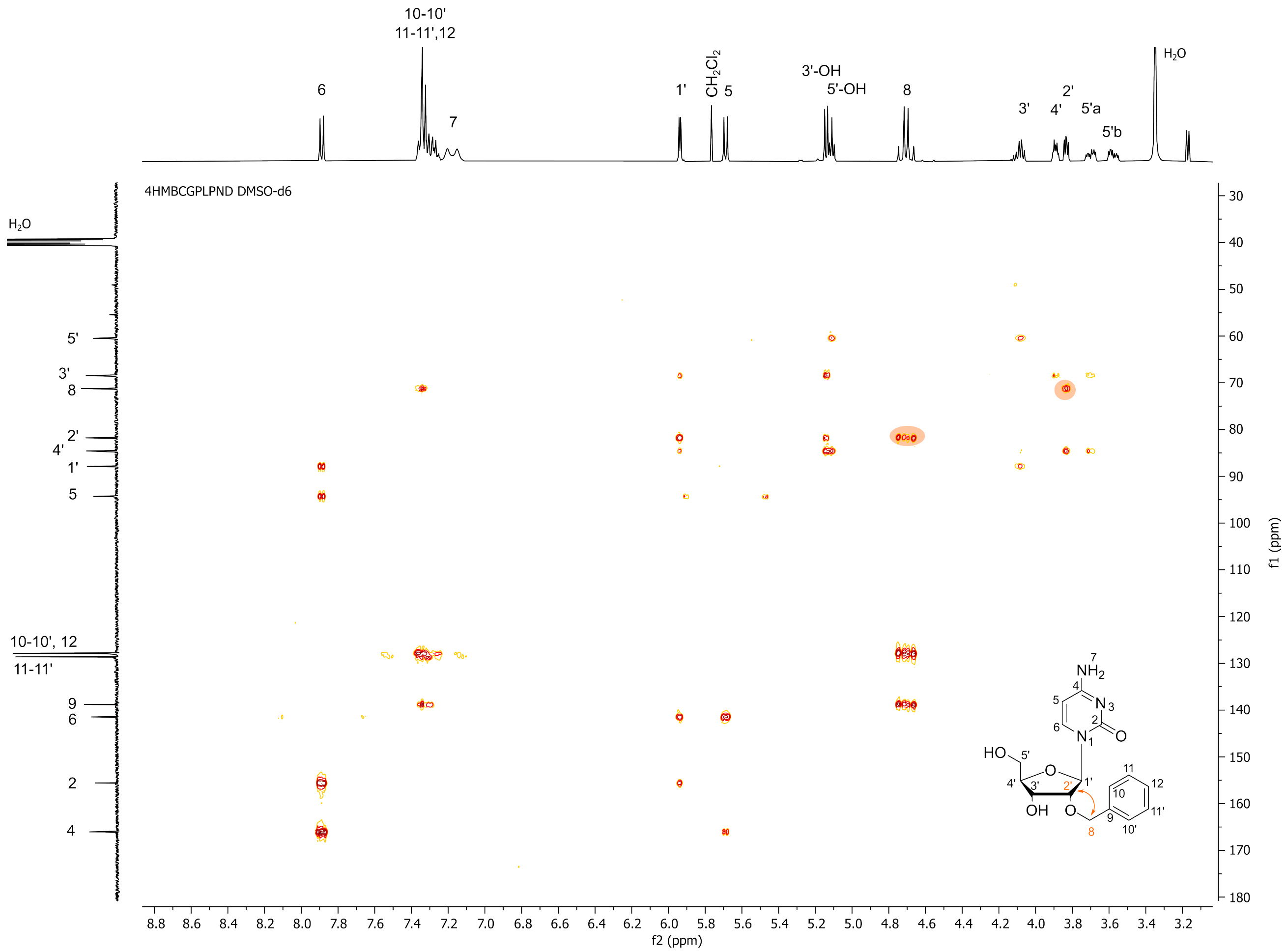

^{1}H-^{13}C HMBC NMR (400 MHz, DMSO-*d6*) of compound **2'*O*BnC** (zoomed 3.2 – 8.8; 30 – 180 ppm).

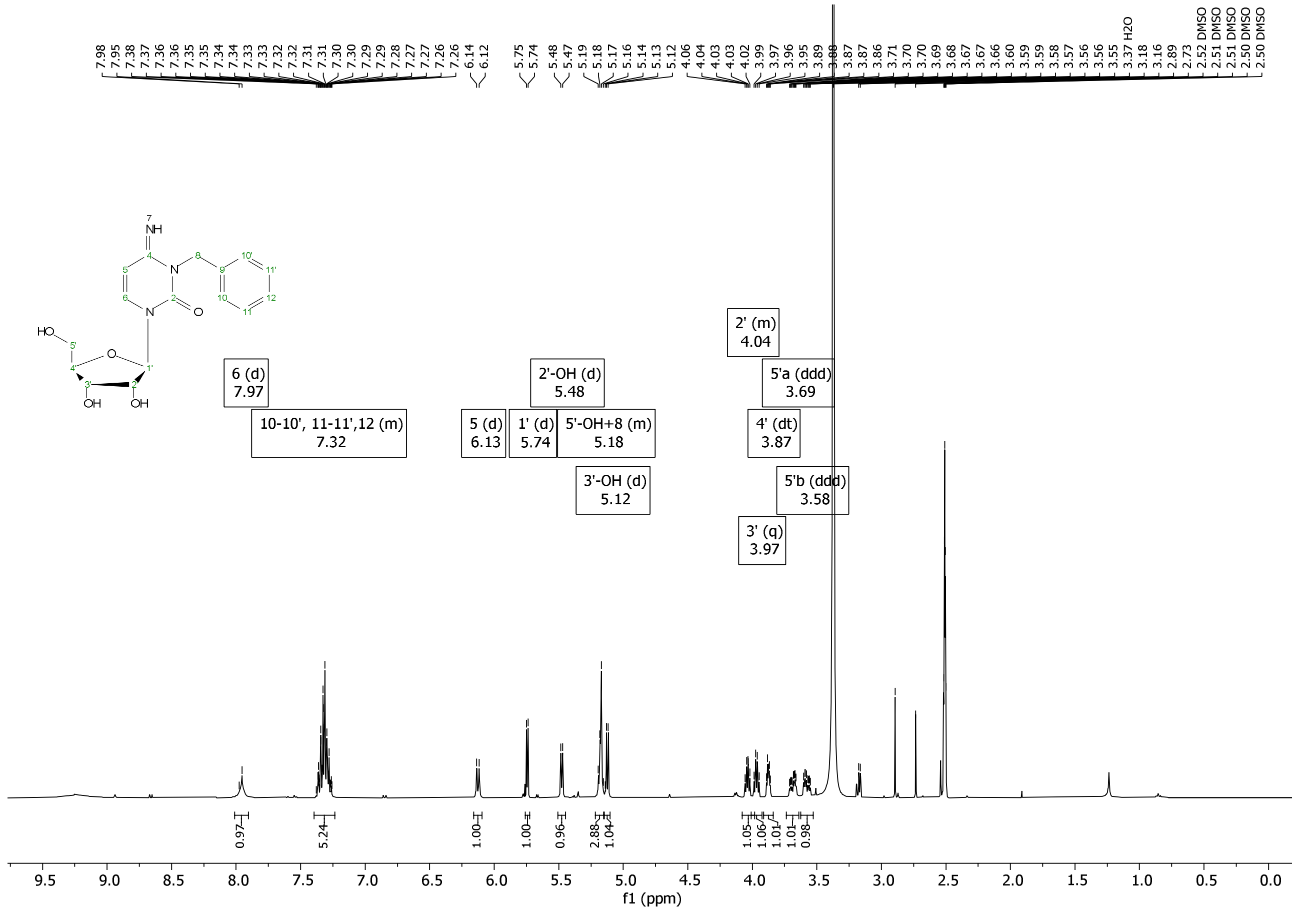

^{1}H NMR (400 MHz, DMSO-*d6*) of compound **N^3BnC**. The NMR signals at 7.95 ppm (s), 2.89 ppm (s), 2.73 ppm (s) correspond to residual *N,N*-dimethylformamide, and the signal at 3.17 (d) corresponds to residual methanol. The signal of the formyl proton from *N,N*-dimethylformamide is overlapped with H-6 proton of **N^3BnC**.

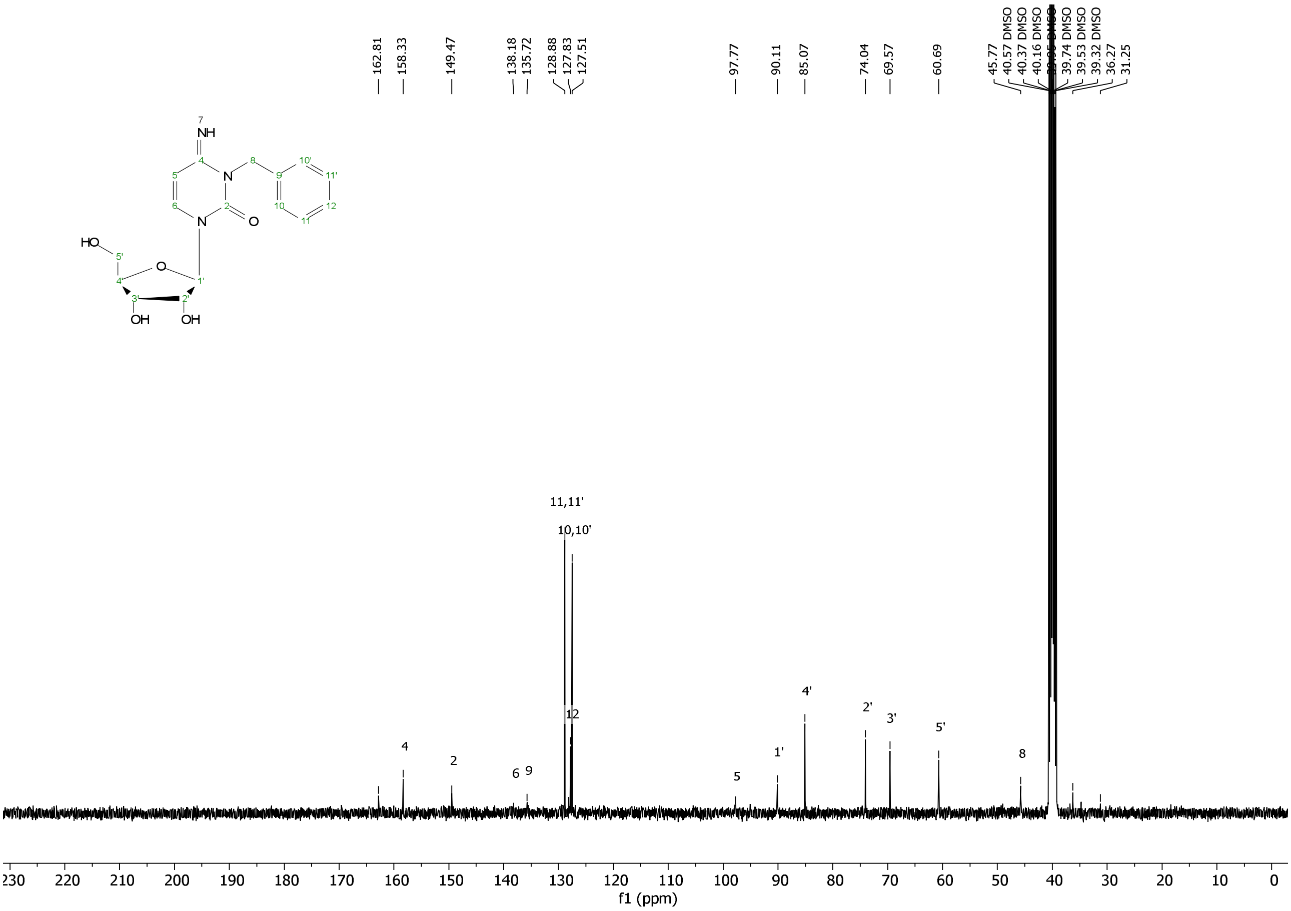

^{13}C {^{1}H} NMR (101 MHz, DMSO-*d6*) of compound ***N*^{3}BnC**. The NMR signals at 162.81 ppm, 36.27 ppm and 31.25 ppm correspond to residual *N,N*-dimethylformamide.

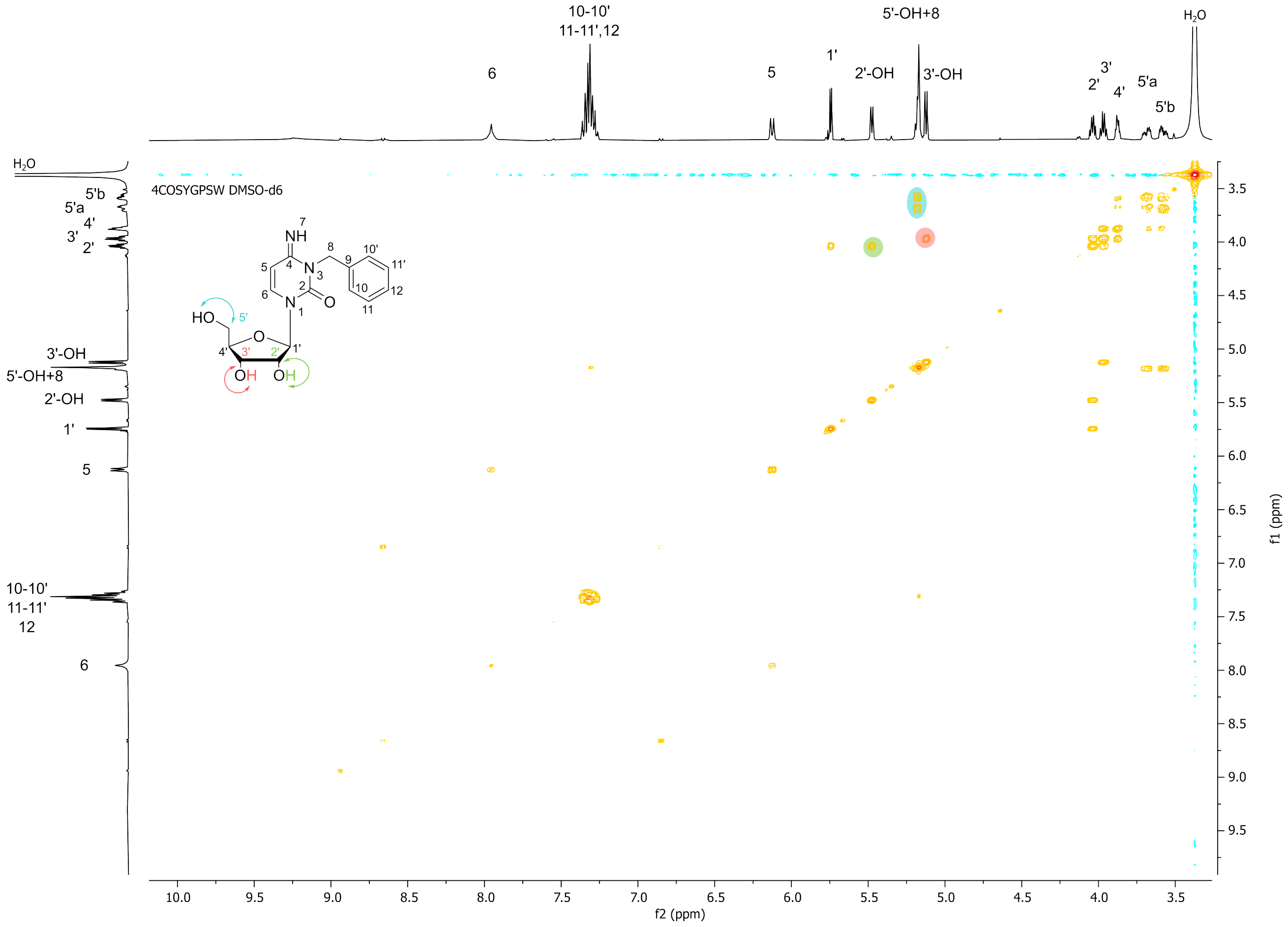

^1H-^1H COSY NMR (400 MHz, DMSO-*d6*) of compound ***N*^3^BnC** (zoomed 3.0 – 10.0 ppm).

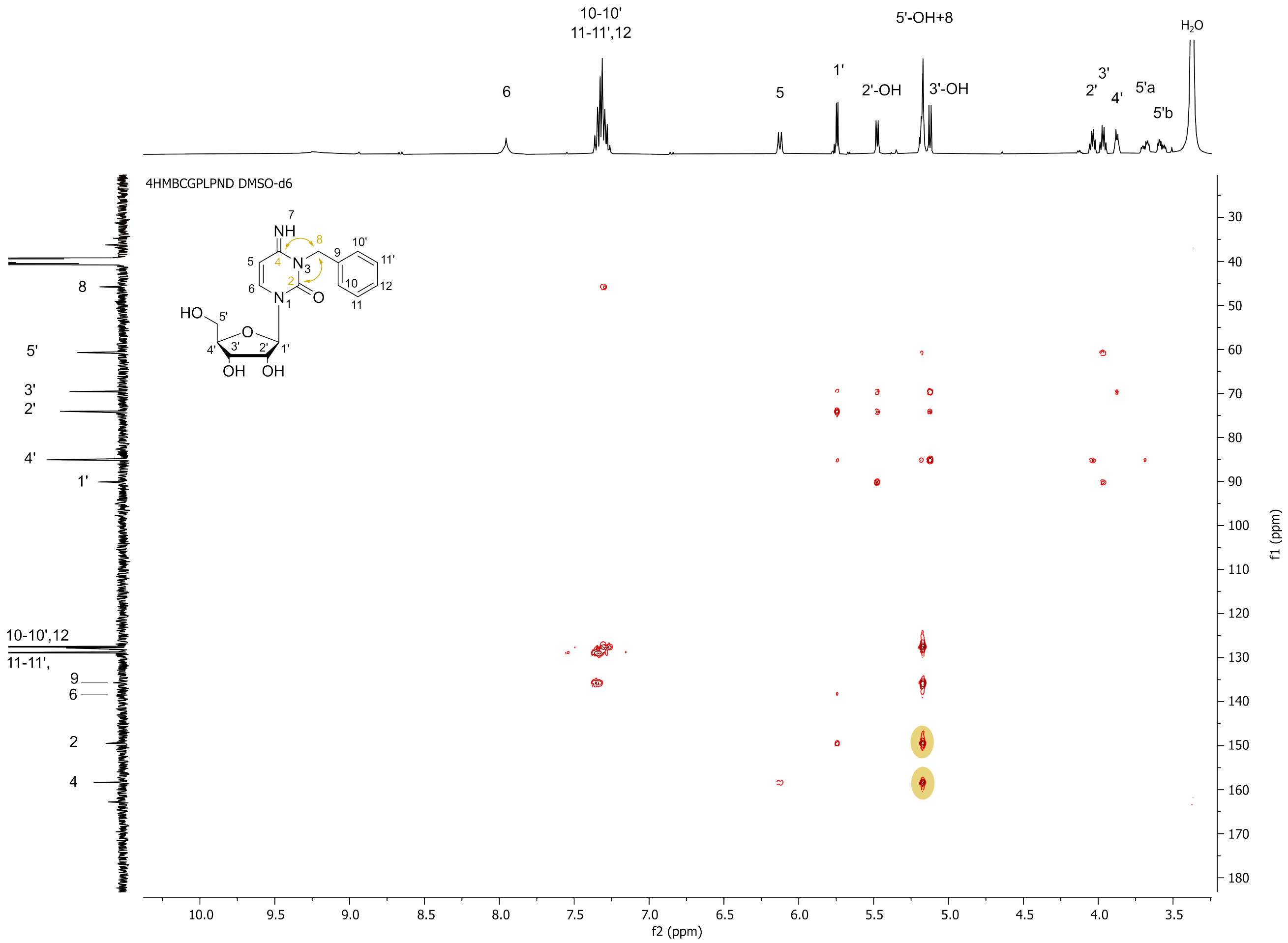

^{1}H-^{13}C HMBC NMR (400 MHz, DMSO-*d6*) of compound ***N*^{3}BnC** (zoomed 3.5 – 10.5; 25 – 180 ppm).

References

[1] D. C. Rio, “Expression and purification of active recombinant T7 RNA polymerase from E. coli” *Cold Spring Harb. Protoc.* **2013**, *2013*, DOI 10.1101/pdb.prot078527.

[2] a) M. B. Walunj, C. P. M. Scheitl, T. Jungnickel, C. Höbartner, “Ribozyme-Catalyzed Site-Specific Labeling of RNA Using O6-alkylguanine SNAP-Tag Substrates” *Angew. Chemie - Int. Ed.* **2025**, *202500257*, DOI 10.1002/anie.202500257.
b) C. P. M. Scheitl, E. Dorinova, S. Christopher, M. B. Walunj, T. Okuda, M. V. Sednev, C. Höbartner, An Unexpected Adenosine-Alkylating Ribozyme Emerged by Target Site Relocation during in Vitro Selection. *J Am Chem Soc* **2025**, https://doi.org/10.1021/jacs.5c13970

[3] C. P. M. Scheitl, M. Ghaem Maghami, A. K. Lenz, C. Höbartner, “Site-specific RNA methylation by a methyltransferase ribozyme” *Nature* **2020**, *587*, 663–667.

[4] M. D. Cole, V. H. Cowling, “Specific regulation of mRNA cap methylation by the c-Myc and E2F1 transcription factors” *Oncogene* **2009**, *28*, DOI 10.1038/onc.2008.463.

[5] M. G. Maghami, C. P. M. Scheitl, C. Höbartner, “Direct in vitro selection of trans-acting ribozymes for posttranscriptional, site-specific, and covalent fluorescent labeling of RNA” *J. Am. Chem. Soc.* **2019**, *141*, DOI 10.1021/jacs.9b10531.

[6] M. Ghaem Maghami, S. Dey, A. K. Lenz, C. Höbartner, “Repurposing Antiviral Drugs for Orthogonal RNA-Catalyzed Labeling of RNA” *Angew. Chemie - Int. Ed.* **2020**, *59*, DOI 10.1002/anie.202001300.

[7] K. Schlosser, J. Gu, L. Sule, Y. Li, “Sequence-function relationships provide new insight into the cleavage site selectivity of the 8-17 RNA-cleaving deoxyribozyme” *Nucleic Acids Res.* **2008**, *36*, DOI 10.1093/nar/gkm1175.

[8] T. Lv, J. Wu, F. Kang, T. Wang, B. Wan, J. J. Lu, Y. Zhang, Z. Huang, “Synthesis and Evaluation of O 2-Derived Diazeniumdiolates Activatable via Bioorthogonal Chemistry Reactions in Living Cells” *Org. Lett.* **2018**, *20*, DOI 10.1021/acs.orglett.8b00423.

[9] X. H. Zhang, Y. Z. Xu, “NMR studies on 4-thio-5-furan-modified and 4-thio-5-thiophene-modified nucleosides” *Magn. Reson. Chem.* **2016**, *54*, DOI 10.1002/mrc.4501.

[10] I. Nowak, M. J. Robins, “Hydrothermal deamidation of 4-N-acylcytosine nucleoside derivatives: Efficient synthesis of uracil nucleoside esters” *Org. Lett.* **2005**, *7*, DOI 10.1021/ol0518378.

[11] a) A. M. Jawalekar, M. O. de Beeck, F. L. V. Delft, A. Madder, “Synthesis and incorporation of a furan-modified adenosine building block for DNA interstrand crosslinking” *Chem. Commun.* **2011**, *47*, DOI 10.1039/c0cc04667a.
b) W. Hutzenlaub, W. Pfleiderer, “Nucleosides .8. Synthesis of 2'-O-, Benzylctidine 3'-O-Benzylcytidine and 5'-O-Benzylcytidine“ *Chem Ber-Recl.* **1973**, 106, 665-673.

[12] a) T. Fujii, T. Itaya, T. Saito, “Purines. XVII. Kinetic Studies of the Base-Catalyzed Conversion of 1-Alkyladenosines into N-Alkyladenosines: Effect of Substituents on the Rearrangement Rate” *Chem. Pharm. Bull.* **1975**, *23*, DOI 10.1248/cpb.23.54.
b) R. Shapiro, S. J. Shiuey, “Reactions of Cytidine with 7-Bromomethylbenz[a]Anthracene, Benzyl Bromide, and Para-Methoxybenzyl Bromide - Ratio of Amino to 3 Substitution“ *J Org Chem*. **1976**, 41, 1597-1600.

[13] A. R. Gruber, R. Lorenz, S. H. Bernhart, R. Neuböck, I. L. Hofacker, “The Vienna RNA websuite.” *Nucleic Acids Res.* **2008**, *36*, DOI 10.1093/nar/gkn188.
