# Supplementary material for: Alkyltransferase Ribozyme for Site‐Specific N 4‐Cytidine Alkylation
Source: Angew Chem Int Ed Engl. 2026 Apr 6;65(20):e6447137. doi: 10.1002/anie.6447137 (PMC13159434; doi:10.1002/anie.6447137)
Supplement: Supplementary file 2 — Supporting File 2: anie72108‐sup‐0002‐CSAR_Supplement_Uncropped images.pdf. [file ANIE-65-e6447137-s002.pdf]

Uncropped images (full scans) of gels used in Figures 2C, 2H, 3B, 4B and SI Figures S1C, S2B–C, S3A, S3C–D, S6D&G, S7A&D, S9B–D. The regions used are indicated by red boxes and labeled with the corresponding RNA substrates and ribozymes according to the numbers in Supplementary Tables S3 – S4.

Full-size images for Figure 2

Figure 2C

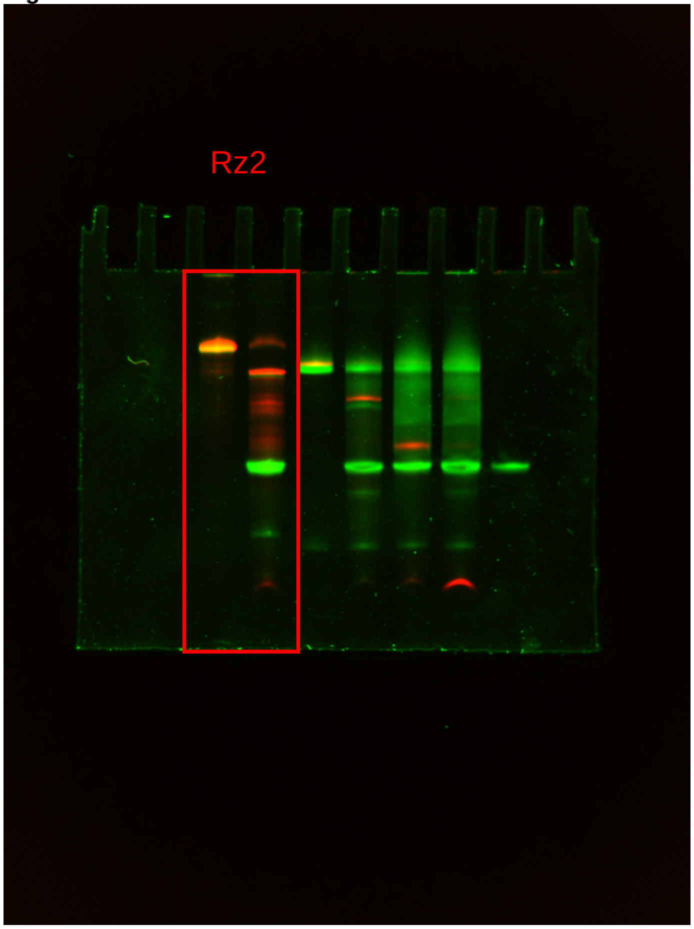

15% PAGE, 10 × 8 cm, 200 V, 70 min, stained with SYBR Gold, dual channel

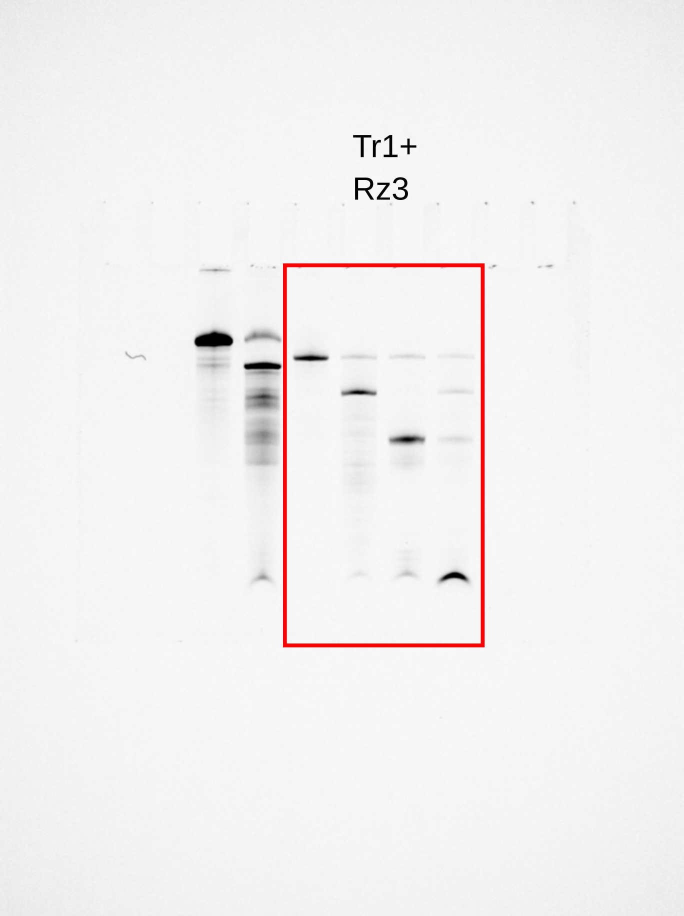

15% PAGE, 10 × 8 cm, 200 V, 70 min, stained with SYBR Gold, Cy5 channel

Figure 2H. 15% PAGE, 10 × 30 cm, 2 h 30 m, imaged by autoradiography

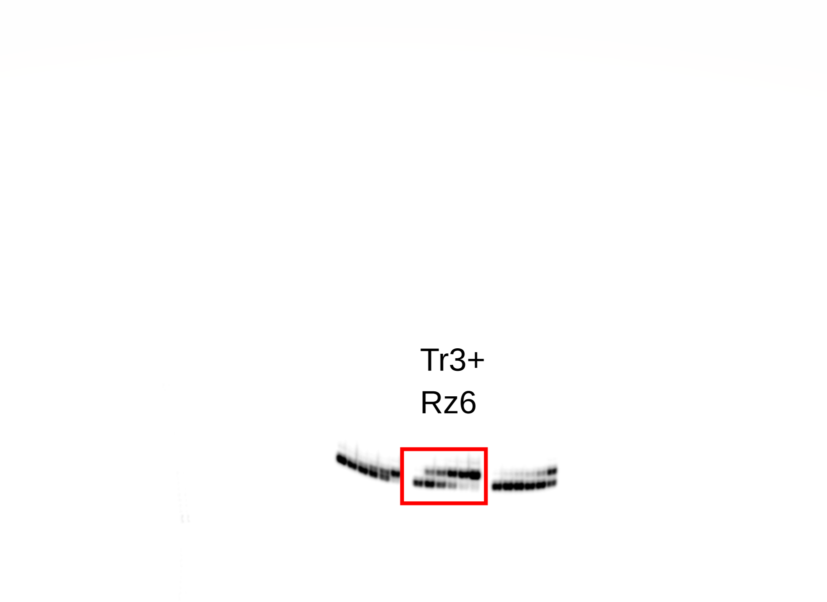

Full-size images

Figure 3B

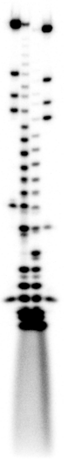

15% PAGE, 10 × 40 cm, 1 h 20 m, imaged by autoradiography

Figure 4B: 5'-^{32}P- labeled RNA + ribozyme with cofactor 5

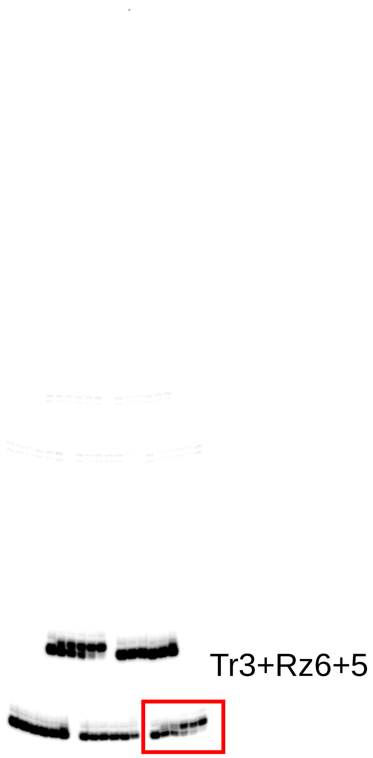

15% PAGE, 10 × 40 cm, 3 h, imaged by autoradiography

Figure 4B: 3'-fluorescently labeled RNA (Tr3) + ribozyme with cofactor 6 or 7

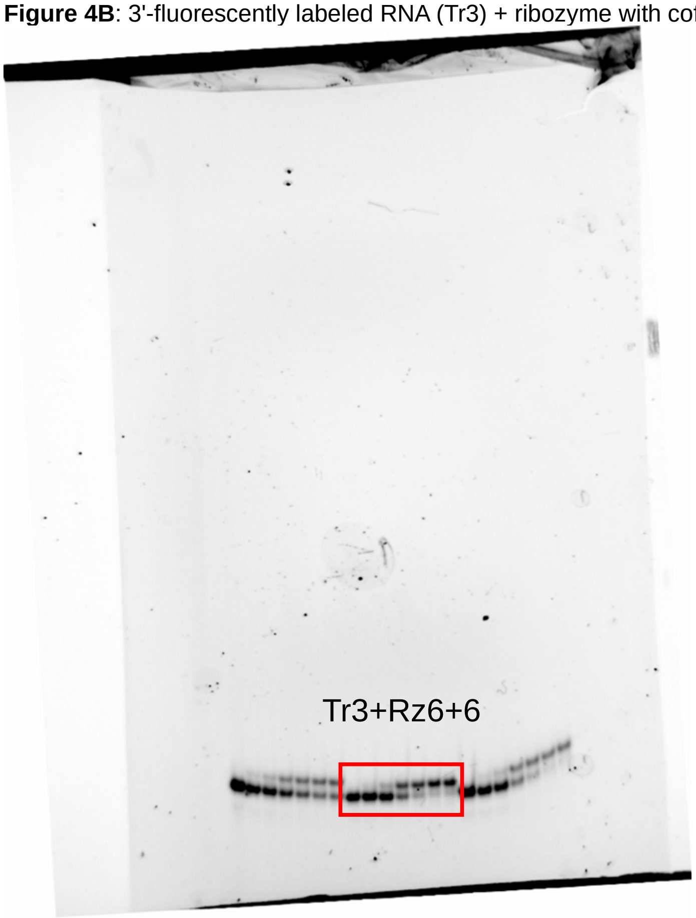

15% PAGE, 10 × 40 cm, 2 h 30 m, fluorescence imaging

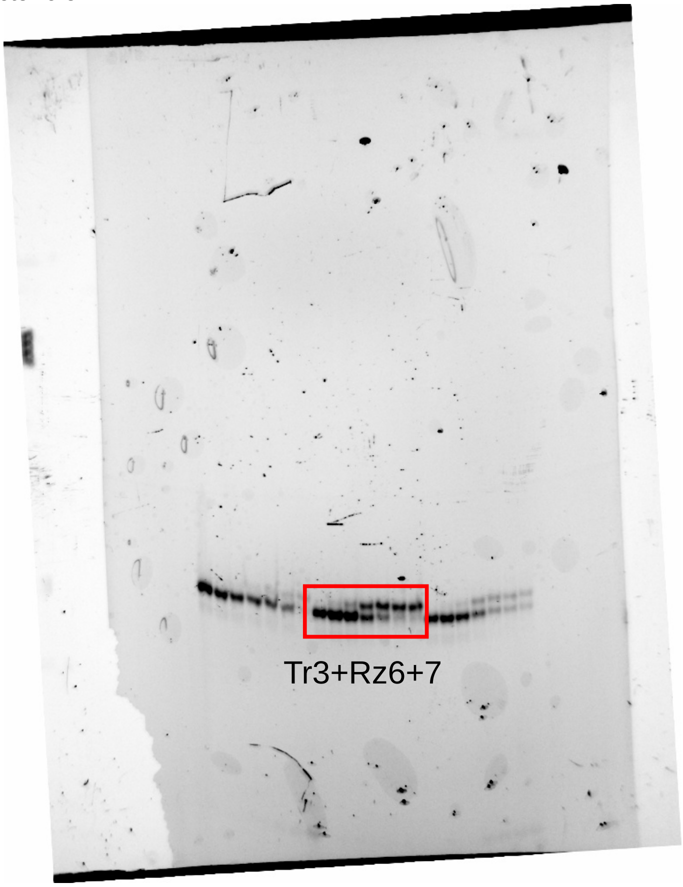

15% PAGE, 10 × 40 cm, 2 h 30 m, fluorescence imaging

Full-size images for Figure S1C

10% native PAGE, 10 × 8 cm, 200 V, 45 min, stained with SYBR Gold.

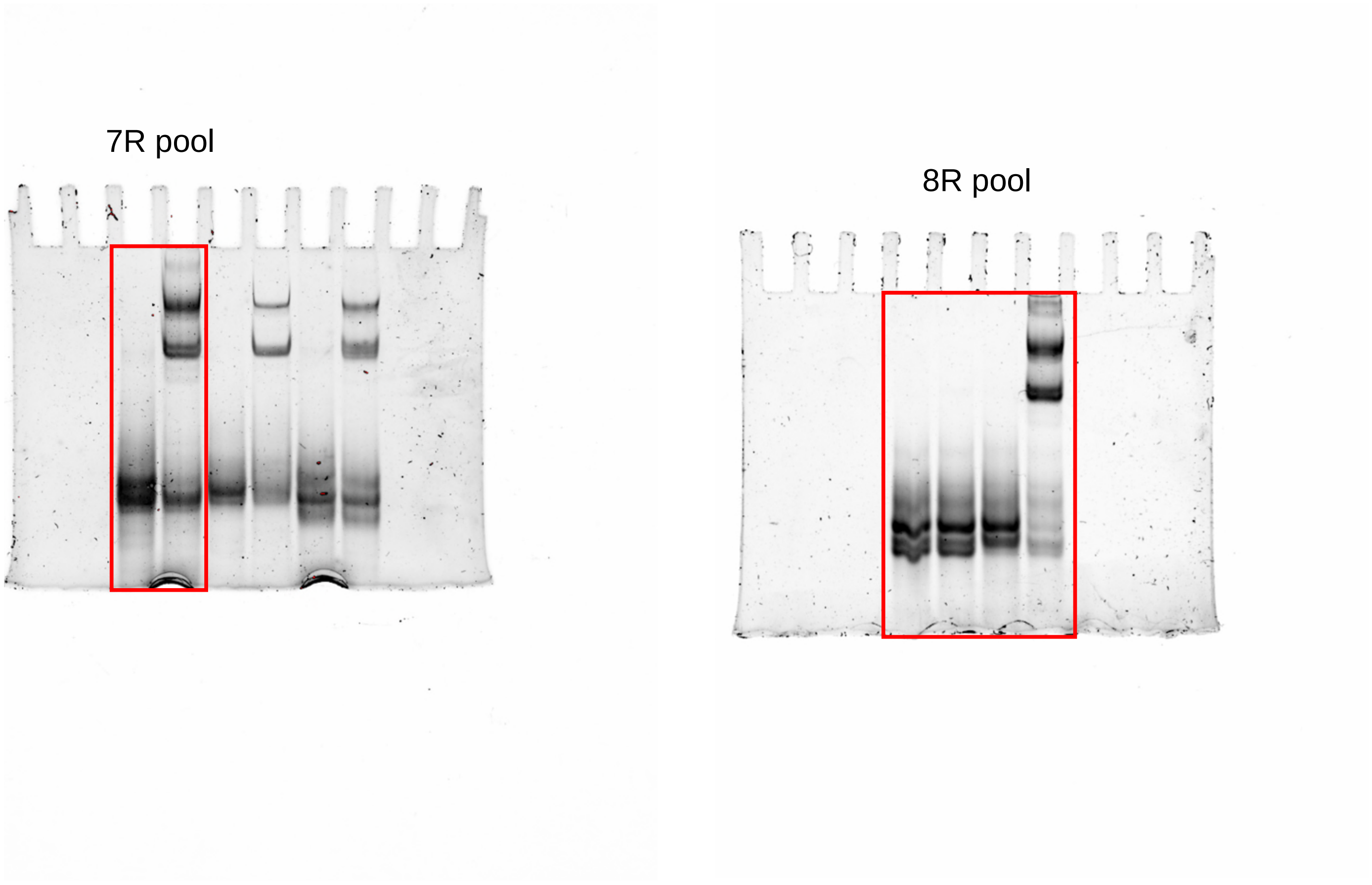

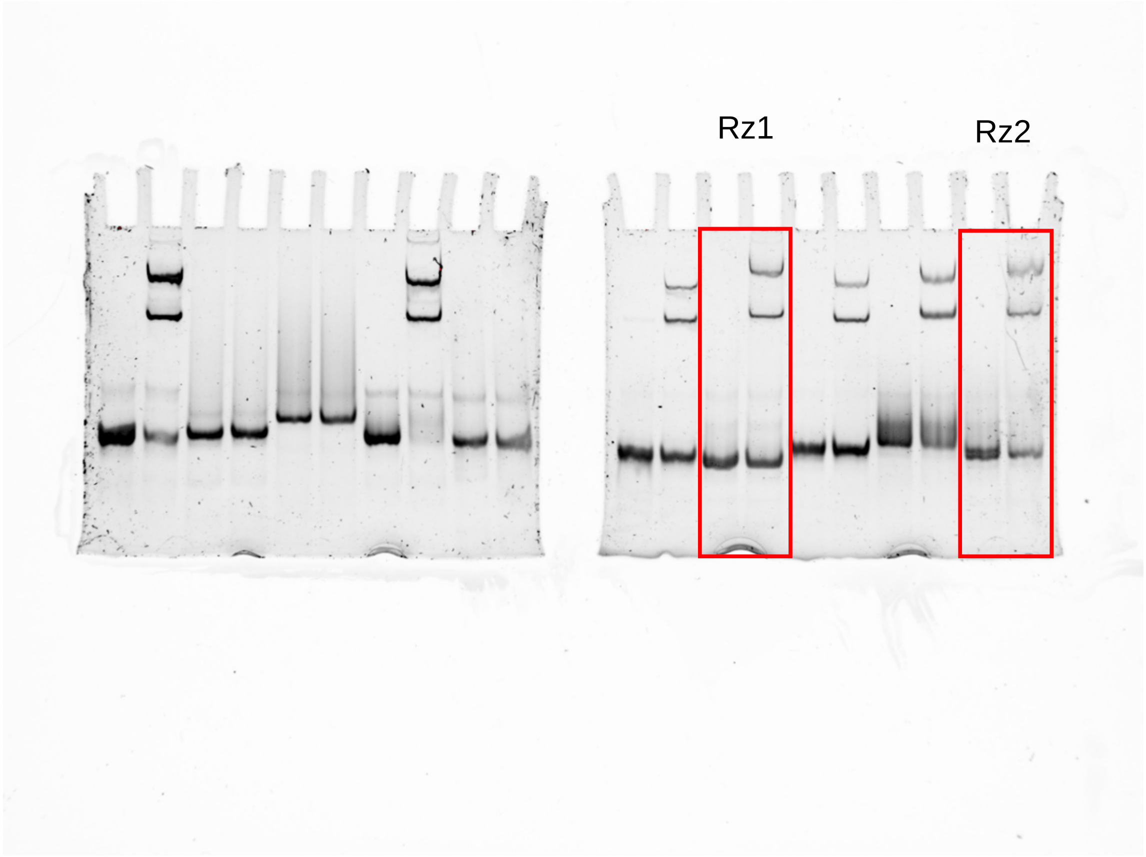

Full-size images for Figure S2: 5'-^{32}P-labeled DNA primers.

Figure S2B

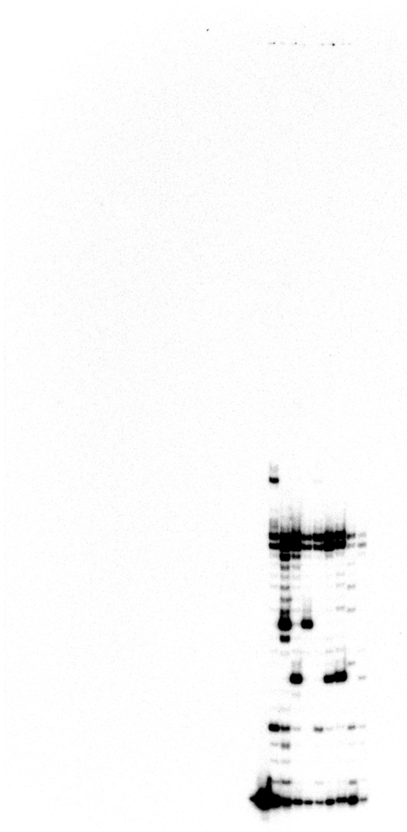

15% PAGE, 20 × 40 cm, 45 W, 2 h 40 min

Figure S2C

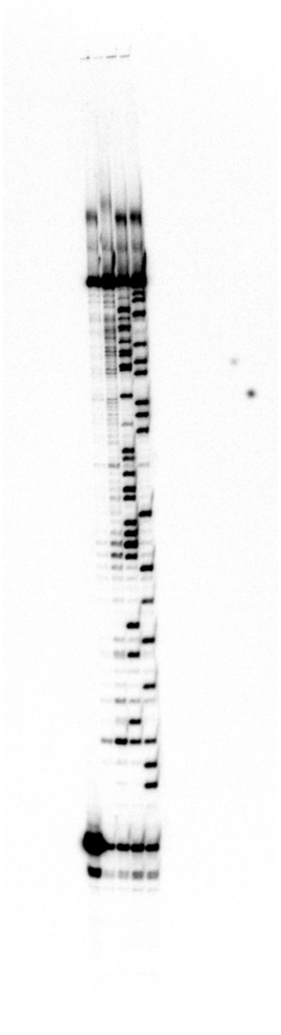

20% PAGE, 20 × 40 cm, 45 W, 3 h 30 min

Full-size images for Figure S3A: gels were stained with SYBR Gold

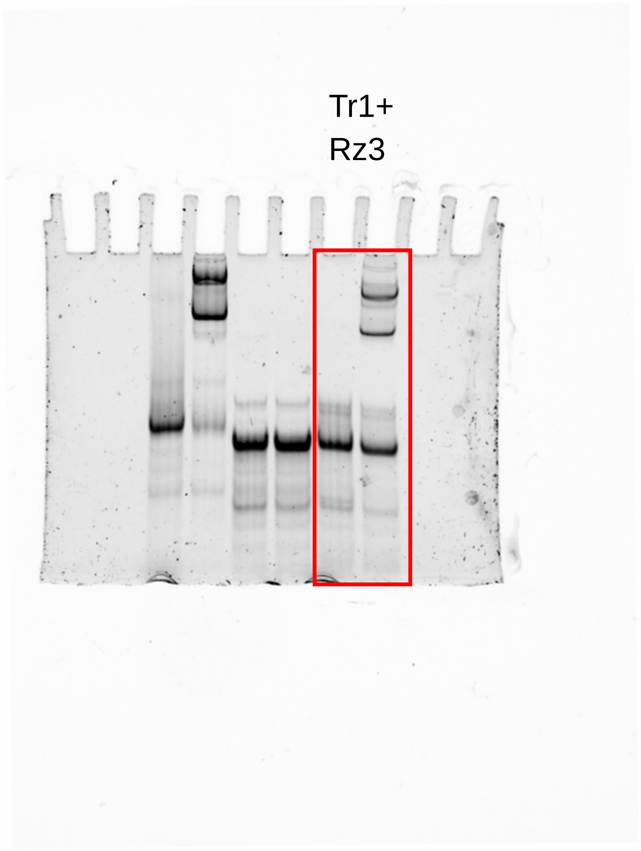

10% native PAGE, 10 × 8 cm, 200 V, 45 min

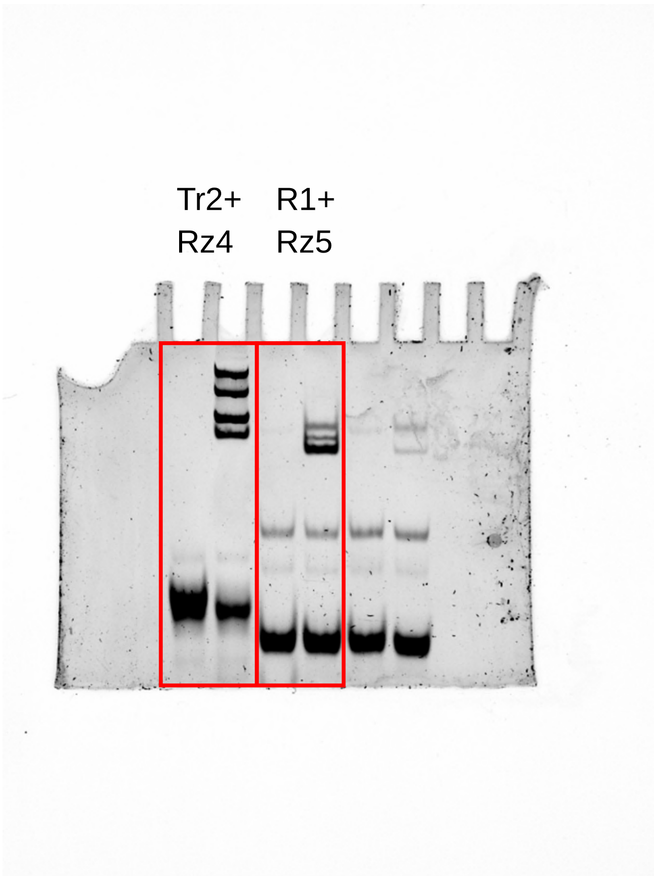

10% native PAGE, 10 × 8 cm, 200 V, 30 min

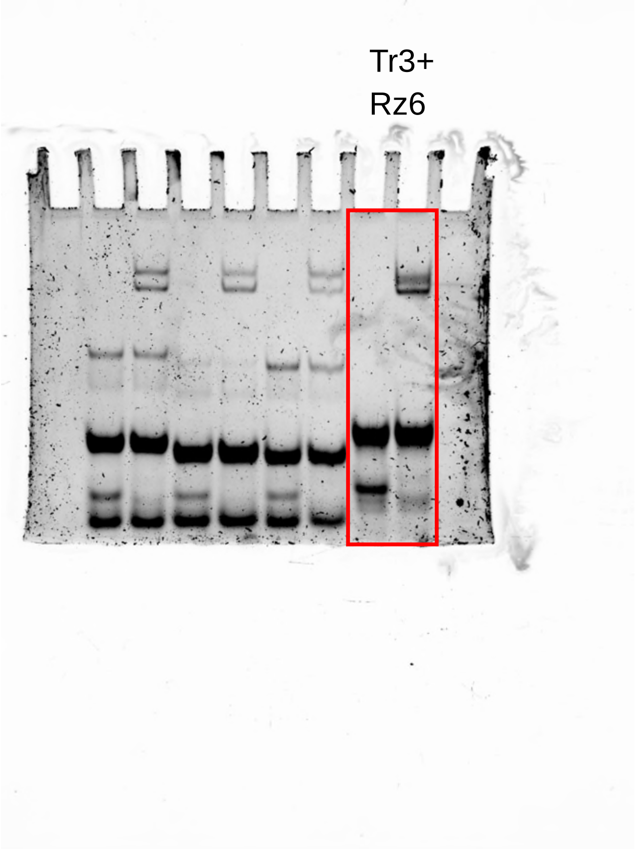

10% native PAGE, 10 × 8 cm, 200 V, 30 min

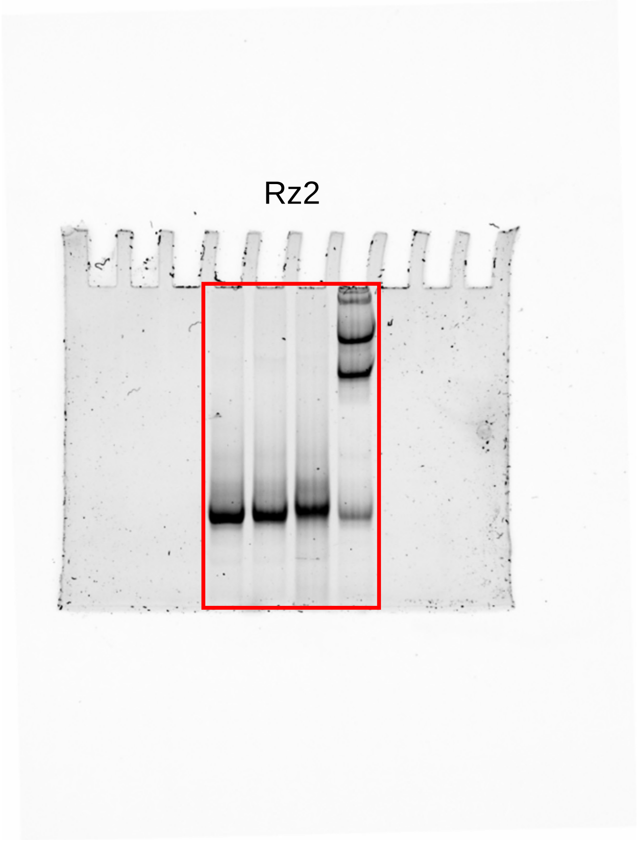

10% native PAGE, 10 × 8 cm, 200 V, 45 min

Full-size images for Figure S3: 5'-^{32}P-/3'-fluorescently labeled RNA

Figure S3C

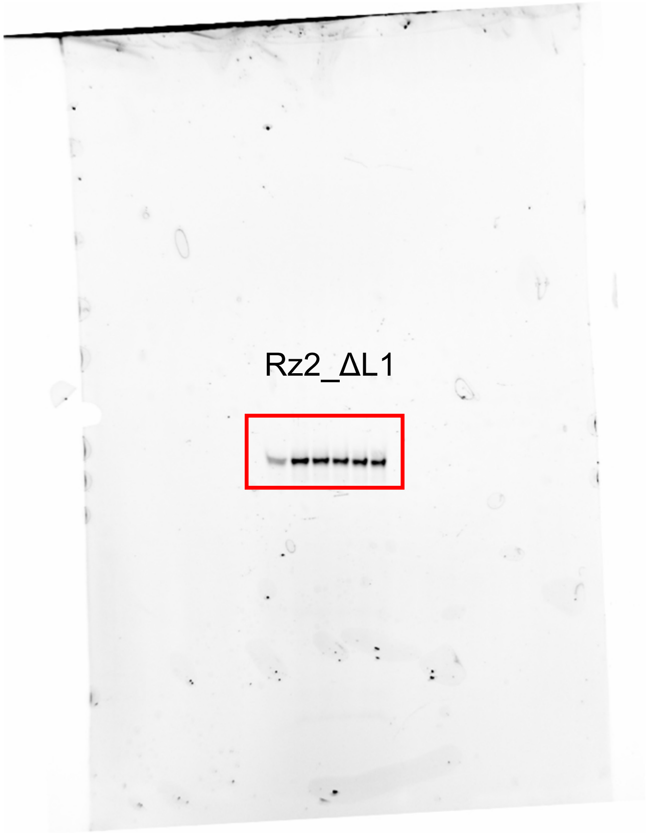

20% PAGE, 10 × 30 cm, 2 h 40 m, fluorescence imaging

Figure S3D

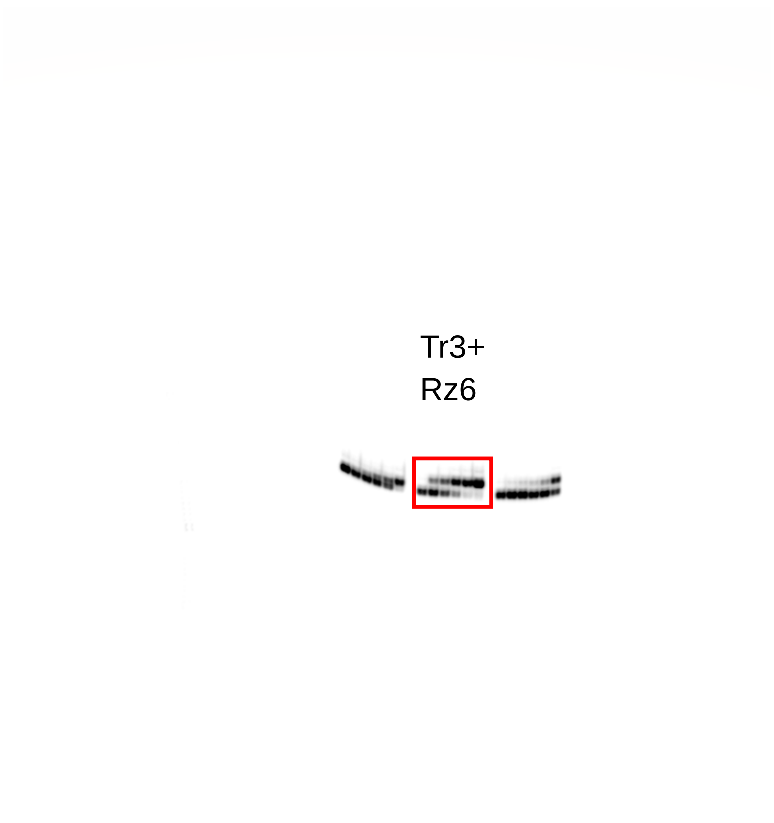

15% PAGE, 10 × 30 cm, 2 h 30 m, imaged by autoradiography

Figure S3D

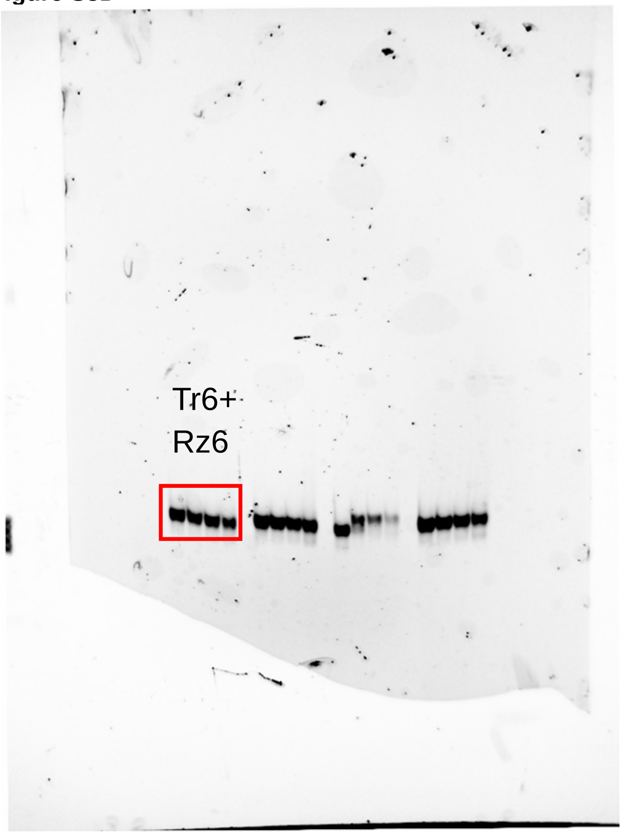

20% PAGE, 10 × 30 cm, 2 h 50 m, fluorescence imaging

Figure S3D

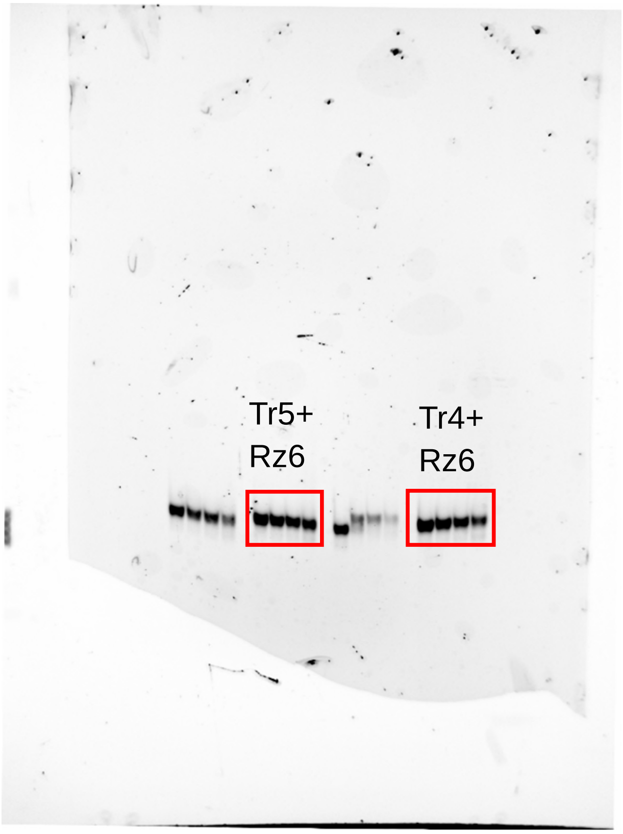

20% PAGE, 10 × 30 cm, 2 h 50 m, fluorescence imaging

Full-size images for Figure S6D: 5'-^{32}P-/3'-fluorescently labeled RNA (Tr3) + CSAR (Rz6) with cofactors (2 – 8).

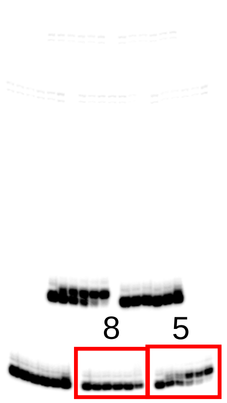

15% PAGE, 10 × 40 cm, 3 h, imaged by autoradiography

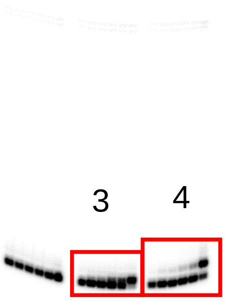

15% PAGE, 10 × 40 cm, 3 h, imaged by autoradiography

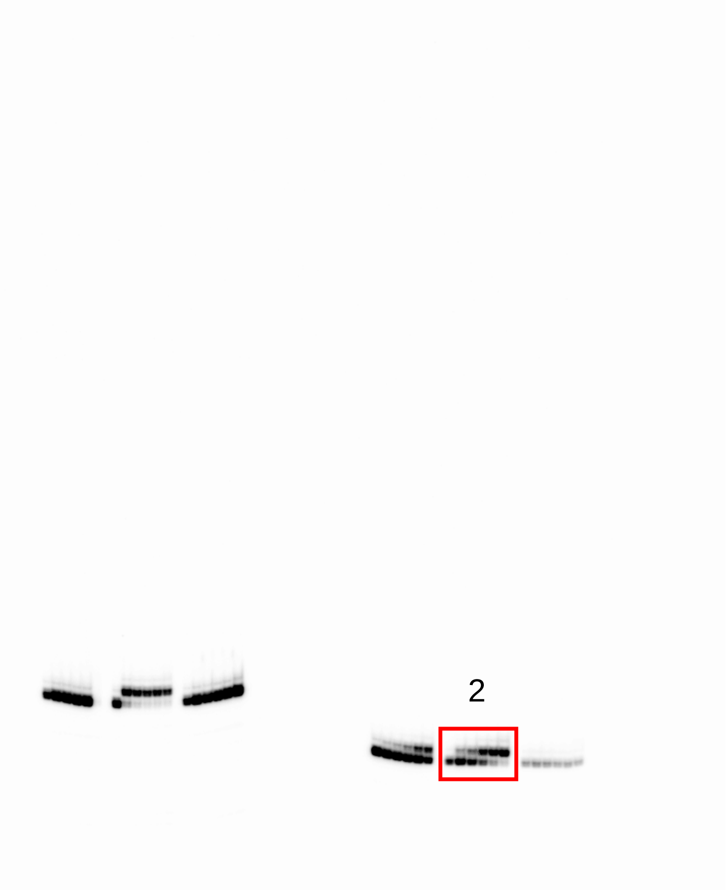

15% PAGE, 10 × 40 cm, 2 h 40 m, imaged by autoradiography

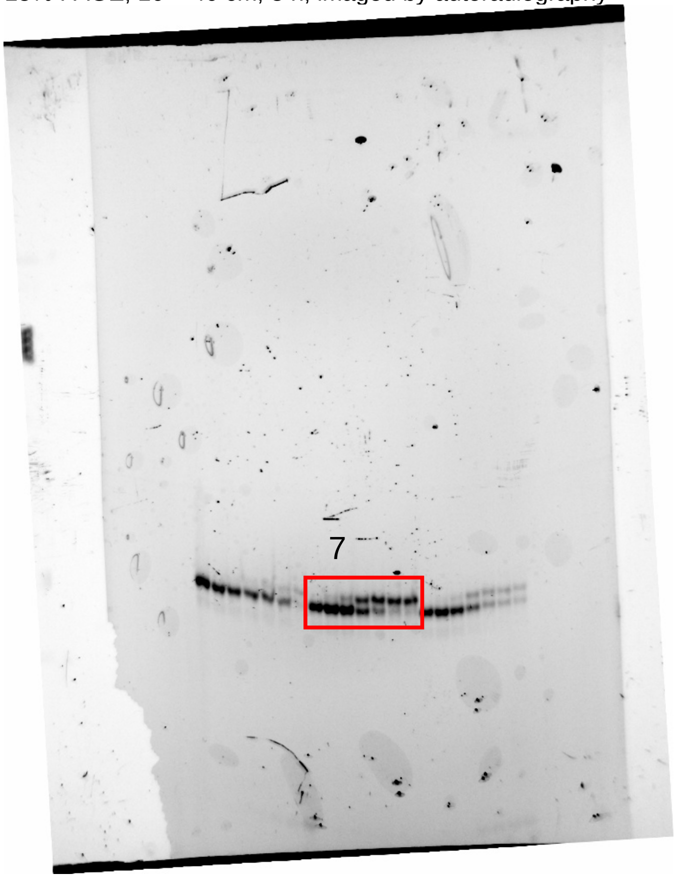

15% PAGE, 10 × 40 cm, 2 h 30 m, fluorescence imaging

Full-size images: 3'-fluorescently labeled RNA (Tr3) + CSAR (Rz6)

Figure S6D, cofactor 6

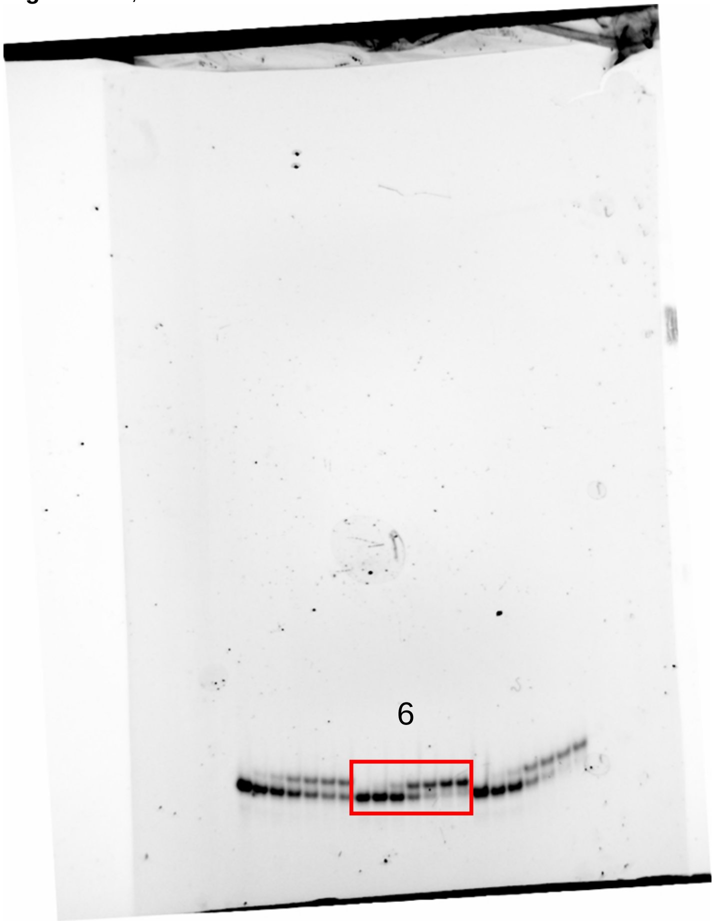

15% PAGE, 10 × 40 cm, 2 h 30 m, fluorescence imaging

Figure S6G, cofactor 2, 40 mM $MgCl_2$

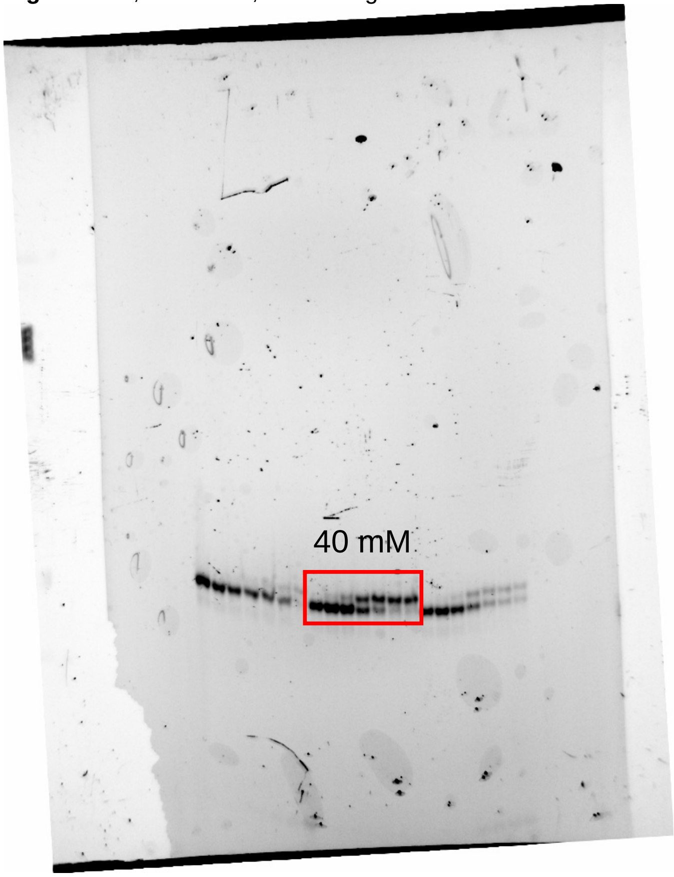

15% PAGE, 10 × 40 cm, 2 h 30 m, fluorescence imaging

Figure S6G: 3'-fluorescently labeled RNA (Tr3) + CSAR (Rz6) with cofactor 2 at $MgCl_2$ concentrations (5 mM, 10 mM, 20 mM)

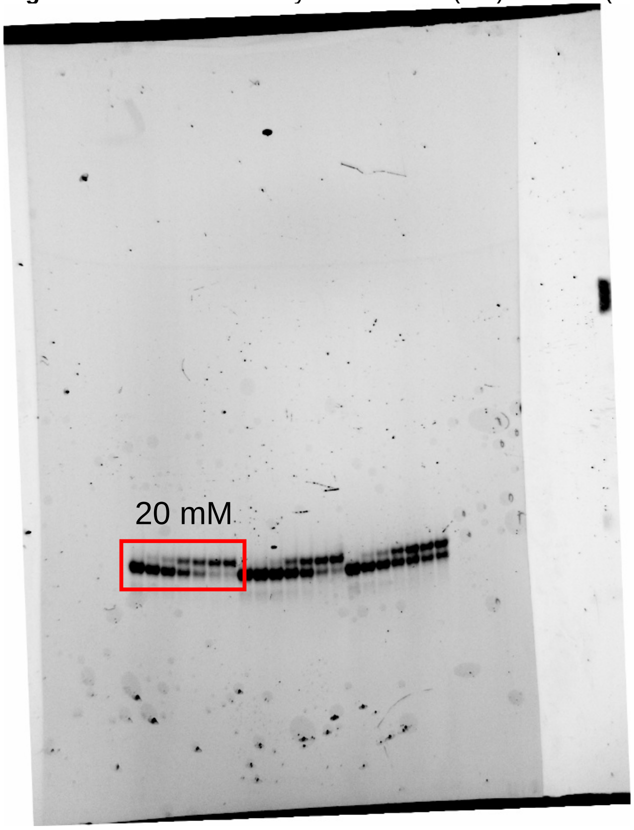

15% PAGE, 10 × 40 cm, 2 h 30 m, fluorescence imaging

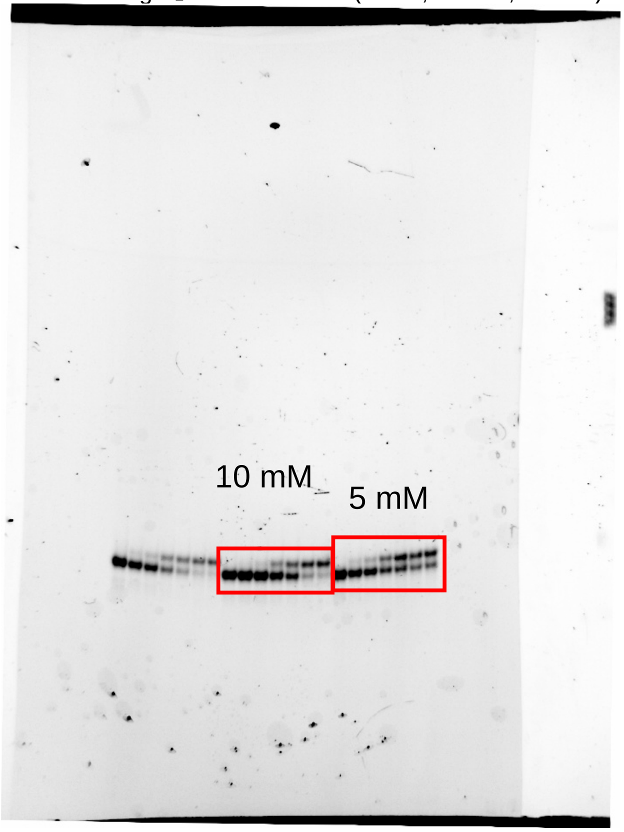

15% PAGE, 10 × 40 cm, 2 h 30 m, fluorescence imaging

Full-size images for Figure S7: 5'-^{32}P-labeled RNA (Tr3) + CSAR (Rz6)

Figure S7A: different ratio of cofactors 2 to 8

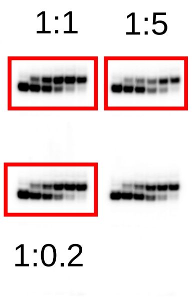

15% PAGE, 10 × 40 cm, 2 h 40 m, imaged by autoradiography

Figure S7D: cofactor 2 at concentrations (20 µM and 400 µM)

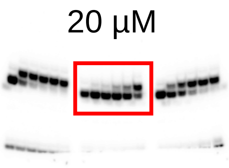

15% PAGE, 10 × 40 cm, 3 h, imaged by autoradiography

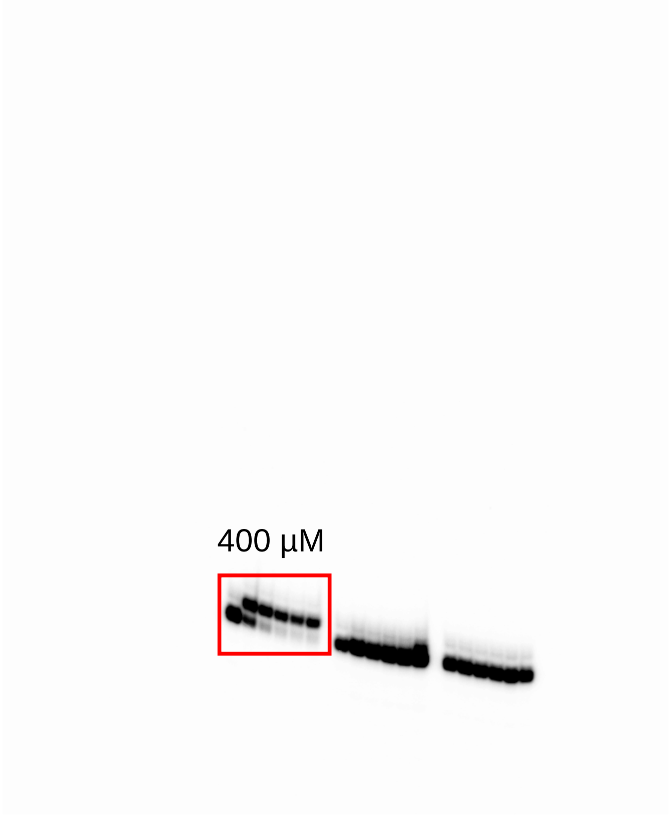

15% PAGE, 10 × 40 cm, 2 h 40 m, imaged by autoradiography

Full-size images for Figure S9

Figure S9B

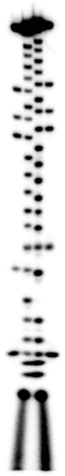

15% PAGE, 10 × 40 cm, 1 h 25 m, imaged by autoradiography

Figure S9C

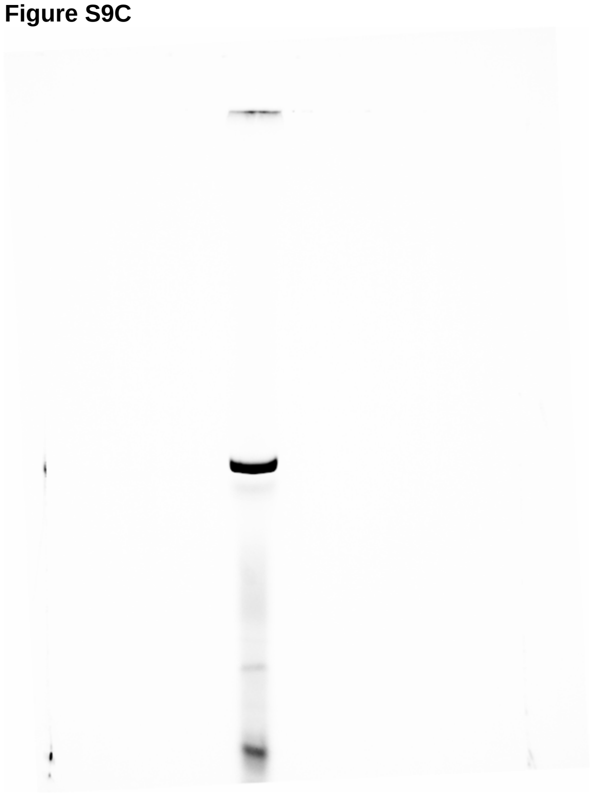

20% PAGE, 10 × 30 cm, 2 h 20 m, FAM channel

Figure S9C

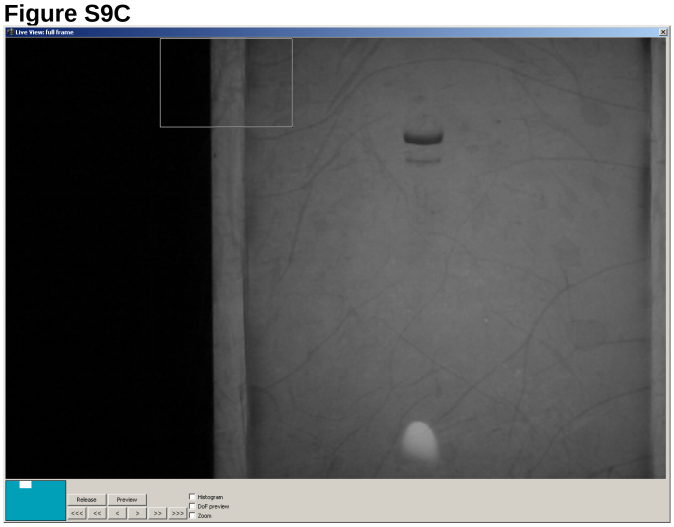

20% PAGE, 10 × 30 cm, 2 h 20 m, UV channel

Figure S9C

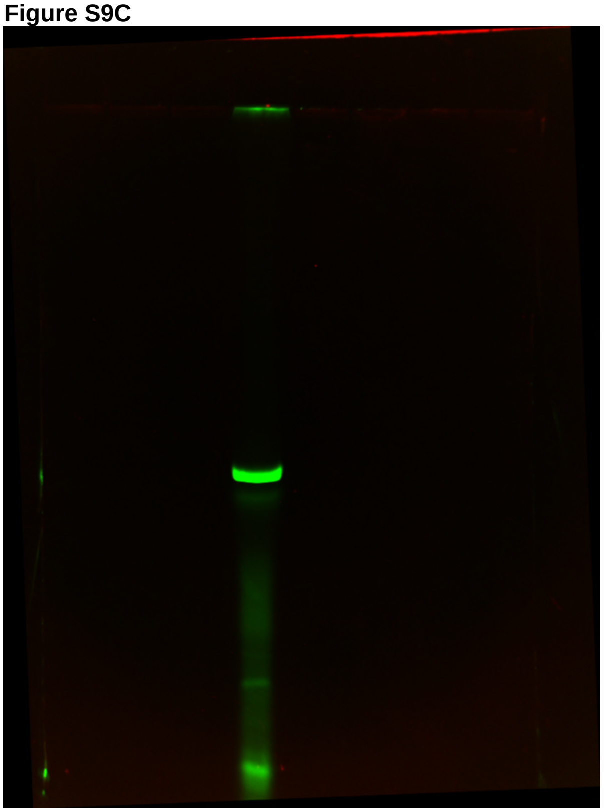

20% PAGE, 10 × 30 cm, 2 h 20 m, dual channel

Full-size images for Figure S9D: 3'-fluorescently labeled RNAs (R2)

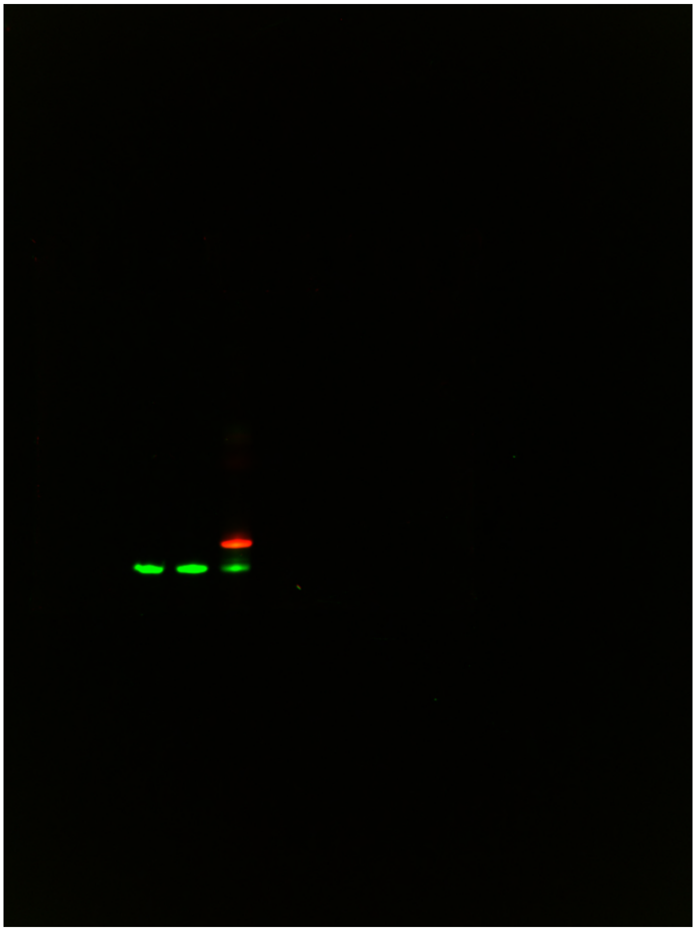

15% PAGE, 10 × 8 cm, 200 V, 80 min, dual channel

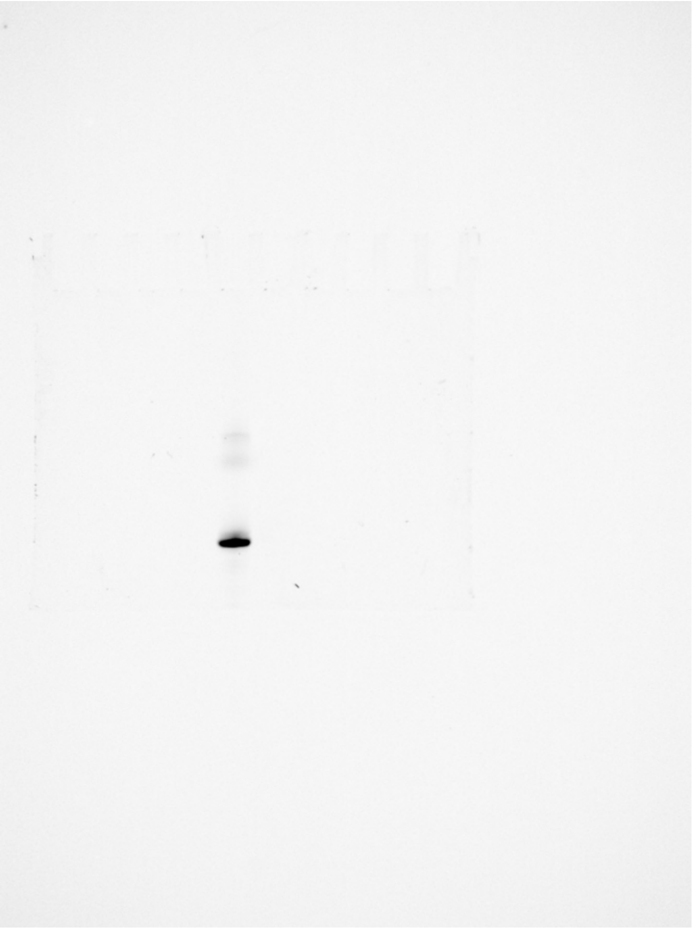

15% PAGE, 10 × 8 cm, 200 V, 80 min, Cy5 channel
